# Covalently Integrated Selenoviologen–*N*‐Heterocyclic Carbene–Pt Nanoparticles for Visible‐Light‐Driven Hydrogen Evolution

**DOI:** 10.1002/advs.76777

**Published:** 2026-07-21

**Authors:** Wenxi He, Guoping Li, Chenjing Liu, Yu Liu, Yawen Li, Wenxin Wei, Hairui Lei, Ni Yan, Gang He

**Affiliations:** ^1^ Frontier Institute of Science and Technology Interdisciplinary Research Center of Frontier Science and Technology State Key Laboratory For Strength and Vibration of Mechanical Structures Institute of New Concept Sensors and Molecular Materials Shaanxi Key Laboratory of New Conceptual Sensors and Molecular Materials Engineering Research Center of Key Materials For Efficient Utilization of Clean Energy of Shaanxi Province Xi'an Key Laboratory of Electronic Devices and Material Chemistry Xi'an Jiaotong University Xi'an Shaanxi P. R. China; ^2^ Shengzhou Yangtze River Delta Institute For the Integration of Industry and Education in New Energy Shengzhou Zhejiang P. R. China; ^3^ Shaanxi Qinzhou Nuclear and Radiation Safety Technology Co., Ltd. Xi'an Shaanxi P. R. China; ^4^ College of Energy Materials and Chemistry Inner Mongolia University Hohhot P. R. China; ^5^ School of Materials Science & Engineering Chang'an University Xi'an Shaanxi P. R. China

**Keywords:** *N*‐heterocyclic carbene, Pt nanoparticles, selenoviologen, solar H_2_ evolution

## Abstract

To overcome the kinetic charge‐transfer barriers imposed by traditional insulating protecting agents in nanoparticle photocatalysis, we synthesized a covalently integrated, single‐component photocatalyst via a thermally induced reduction method. In this architecture, a hydrophilic selenoviologen (SeV^2+^) moiety, which intrinsically provides multiple redox capabilities, a narrow energy gap, and strong visible light absorption, is directly tethered to Platinum nanoparticles (PtNPs) via Nitrogen heterocyclic carbene (NHC) ligands, yielding the SeV^2+^‐ NHC‐PtNPs. The resulting Pt─C covalent linkages serve a bifunctional role: acting as a structural scaffold for nanoparticle stabilization without introducing insulating layers and providing a direct electronic pathway. Consequently, this design not only preserves the photochemical advantages of the SeV^2+^ moiety but also drives efficient, directional intramolecular electron transfer (IET) to the catalytic Pt core, as confirmed by ultrafast spectroscopic and photoelectrochemical studies. By unifying the photosensitizer, electron mediator, and catalyst into a single entity, the system achieves a high hydrogen production rate (2706 µmol·h^−1^·g^−1^), turnover number (169), and apparent quantum yield (0.9%), demonstrating the potential of this covalent integration strategy for solar energy conversion.

## Graphical abstract

A covalently integrated single‐component photocatalyst is constructed by anchoring selenoviologen to platinum nanoparticles via robust *N*‐heterocyclic carbene ligands. The resulting Pt─C covalent bonds function dually as protective structural scaffolds and direct channels for ultrafast intramolecular electron transfer This precise interfacial engineering enables highly efficient and stable photo‐induced electron transport, driving exceptional solar hydrogen evolution.

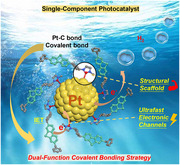

## 1 Introduction

Energy shortages and environmental problems are two significant challenges facing humanity today [1]. Solar energy, a clean and efficient renewable energy source, remains challenging to capture and convert efficiently [2]. The use of artificial photosynthesis systems can achieve the conversion of solar energy to chemical or electrical energy [3, 4]. An ideal photocatalytic system requires not only an efficient photosensitizer, a suitable electron transfer agent, and a catalyst, but also an efficient electron transfer process [5, 6]. Over the past decades, a variety of photosensitizers have shown good light absorption properties in photocatalytic systems [7, 8, 9, 10, 11]. Meanwhile, various advanced photocatalysts have been extensively developed for efficient solar energy conversion [12, 13, 14, 15]. However, the development of electron transfer agents has received less attention, with viologens (V^2+^) considered ideal electron transfer agents in photocatalytic reactions due to their multiple redox states and electron acceptor (A) properties. The first three‐component photocatalytic systems used methyl viologen (MV^2+^) as the electron transfer agent, [Ru(bpy)_3_]^2+^ as the photosensitizer, and a platinum(II) compound as the catalyst [16]. However, the multi‐step electron transfer greatly reduces the electron utilization and thus affects the photocatalytic efficiency. To improve the catalytic efficiency, researchers have developed two‐component catalytic systems based on porphyrinic 2D covalent organic frameworks (MPor‐DETH‐COFs)/8 wt.% H_2_PtCl_6_ or CbV^2+^‐based supramolecular polymers (FeSPs)/PVP‐Pt dimers, as well as one‐component systems with MV^2+^ derivatives covalently linked to Pt(II) complexes [17, 18, 19]. However, some of these simplified systems have weak visible light absorption and poor catalytic ability of tetravalent or divalent platinum, resulting in suboptimal visible light photocatalytic efficiencies.

In order to address these problems, our group previously developed a series of chalcogenoviologens (EV^2+^, E═S, Se, Te) with strong visible light absorption, narrow energy systems and multiple redox centers, particularly selenoviologen (SeV^2+^), which can be used as both photosensitizers and electron transfer agents. This served as a basis for developing a two‐component catalytic system consisting of SeV^2+^ and Pt nanoparticles (PtNPs), simplifying the electron transfer process [20, 21, 22]. While this design improved the overall efficiency, its photocatalytic efficiency is still limited by two main factors. On the one hand, the assembly of SeV^2+^ and PtNPs via electrostatic attraction results in a poorly defined interfacial configuration, leading to unclear intermolecular electron transfer pathways and low transfer rates. On the other hand, to prevent the PtNPs from agglomerating, PtNPs capped with protecting agents (e.g., polymer surfactants like PVP) are conventionally utilized. However, these insulating protective agents cover the catalytically active sites and act as kinetic barriers that hinder interfacial charge transfer [23, 24]. Therefore, we proposed designing SeV^2+^ as a functional ligand to directly connect with PtNPs through covalent bonds, simultaneously achieving efficient intramolecular electron transfer (IET) and the stabilization of PtNPs.

Recently, *N‐*heterocyclic carbene (NHC) has attracted increasing attention as alternative ligands for encapsulated transition metal nanoparticles [25, 26, 27, 28, 29], with their electron‐rich properties [30, 31] combined with the spatial effects [32, 33] forming strong metal‐carbene bonds on the surface of metal nanoparticles (Au─NHC bonds are approximately 90 kJ·mol^−1^ stronger than the corresponding Au‐phosphorus bonds, and twice as strong as the metal‐sulphide bonds in molecular complexes) [34], thus increasing the stability of metal nanoparticles. Furthermore, recent pioneering studies have demonstrated that such NHC‐stabilized noble metal nanoparticles exhibit exceptional stability and tailored surface electronic structures, making them highly promising candidates for advanced catalytic transformations and solar‐to‐chemical energy conversion [35, 36, 37]. From a synthetic perspective, modifying *N‐*heterocyclic is easy and feasible, facilitating the introduction of SeV^2+^ structures. Therefore, we hypothesized that integrating SeV^2+^ with platinum nanoparticles via NHC─Pt covalent bonds would serve a bifunctional role: acting as a structural scaffold to maintain nanoparticle stability without relying on insulating capping agents, while simultaneously providing a direct electronic pathway to facilitate directional IET, thereby improving the overall photocatalytic efficiency.

**FIGURE 1:**
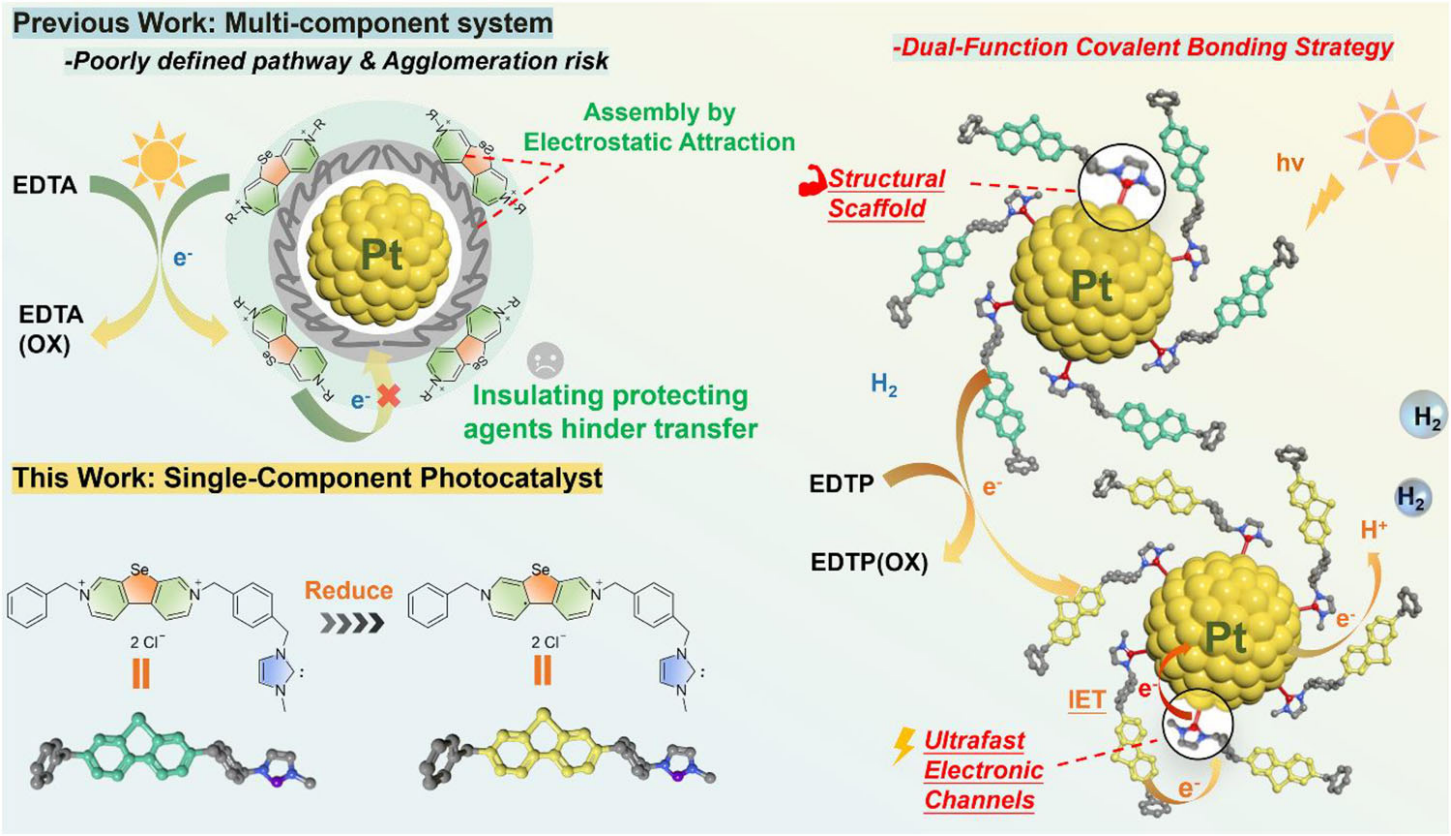
Schematic illustration of **SeV^2+^‐NHC‐PtNPs** in solar H_2_ production.

Based on this strategy, water‐soluble SeV^2+^‐tethered NHC‐stabilized PtNPs (SeV^2+^‐NHC‐PtNPs) were synthesized via a thermally induced reduction method. This covalently integrated architecture preserves the narrow HOMO‐LUMO energy gap, visible‐light absorption, and redox properties of the SeV^2+^ moiety. Furthermore, femtosecond transient absorption (fs‐TA) and photoelectrochemical measurements confirmed that the Pt─C covalent bonds create a stable electronic pathway, enabling efficient IET from the ligand to the Pt core. Consequently, by integrating the photosensitizer, electron mediator, and catalyst into a single entity, the SeV^2+^‐NHC‐PtNPs system demonstrates highly efficient visible‐light‐driven hydrogen evolution, achieving a production rate of 2706 µmol·h^−1^·g^−1^ and an apparent quantum yield (AQY) of 0.9% (Figure 1).

## 2 Results and Discussion

### 2.1 Synthesis and Structural Characterization

**SCHEME 1:**
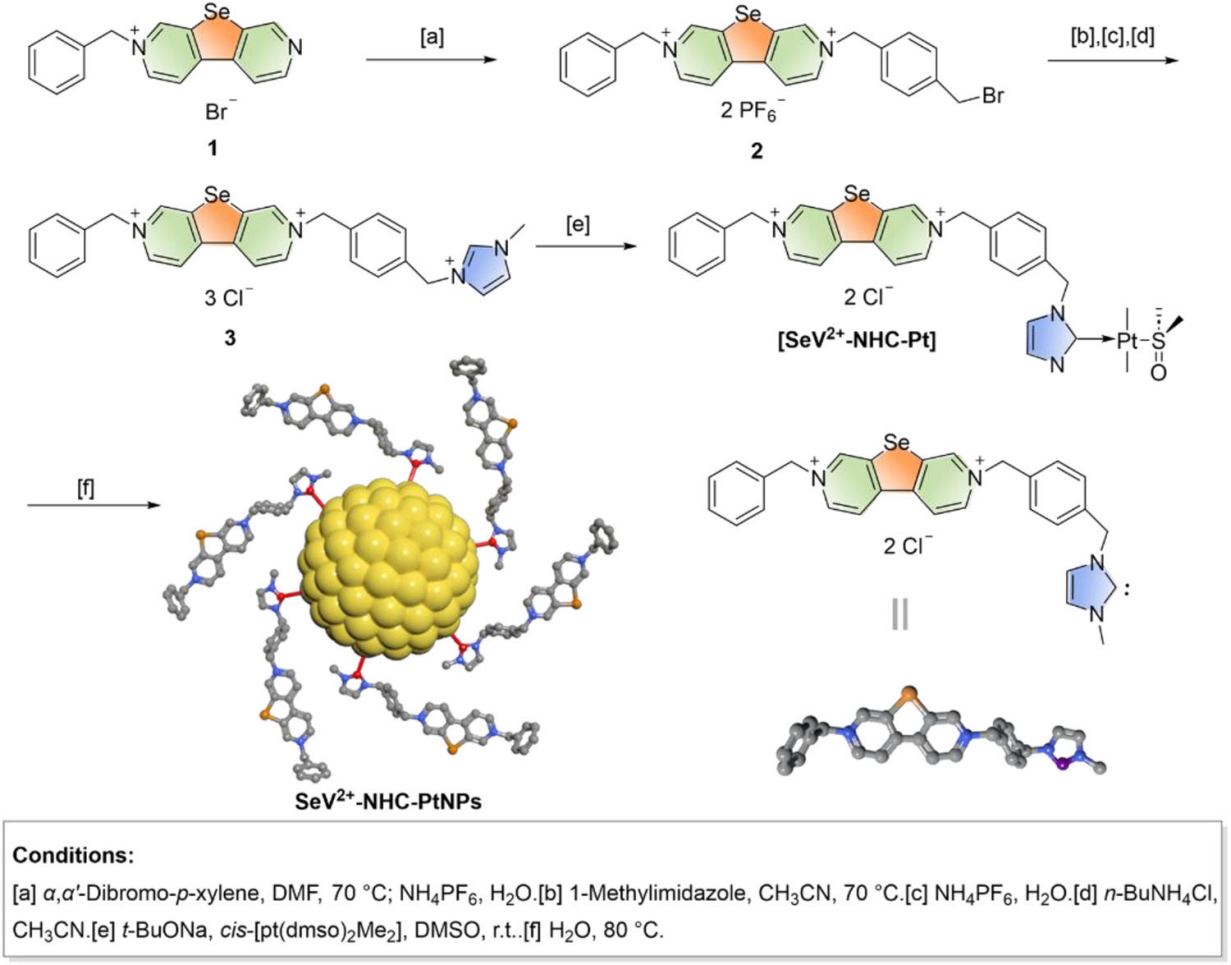
Strategies for the synthesis of water‐soluble **SeV^2+^‐NHC‐PtNPs**.

To obtain the designed platinum nanoparticles modified with SeV^2+^, the first compound **1** was synthesized as a yellow solid powder in 80% yield according to the literature. Next, the compound **2** with PF6^−^ as counterions was synthesized in 61% yield through the reaction between **1** and ɑ,ɑ'‐dibromo‐p‐xylene in a sealed tube at 70°C for 3 days, followed by ion exchange and silica gel chromatography. Compound **3** with Cl^−^ as counterions was obtained by reacting **2** with 1‐methylimidazole, followed by anion exchange with an excess of ammonium hexafluorophosphate (NH_4_PF_6_) and then with an excess of tetrabutylammonium chloride (TBACl); Next, *cis*‐[PtMe_2_(DMSO)_2_] was reacted with a certain amount of imidazolium salt **3** using sodium tert‐butoxide as a deprotonating agent for 1 h at room temperature. The solution was spun dry, and the solid obtained was subjected to a thermal decomposition reaction in water at 80°C. Finally, **SeV^2+^‐NHC‐PtNPs** were obtained by dialysis purification with a yield of 16% (Scheme 1 and Scheme S1). The target compounds were comprehensively verified by ^1^H and ^1^ ^3^C nuclear magnetic resonance (NMR), high‐resolution mass spectrometry (HRMS), Attenuated Total Internal Reflectance Fourier Transform Infrared Spectroscopy (ATR‐FTIR) and thermogravimetric analysis (TGA) (Figures S1 and S2 and S43–S51).

**FIGURE 2:**
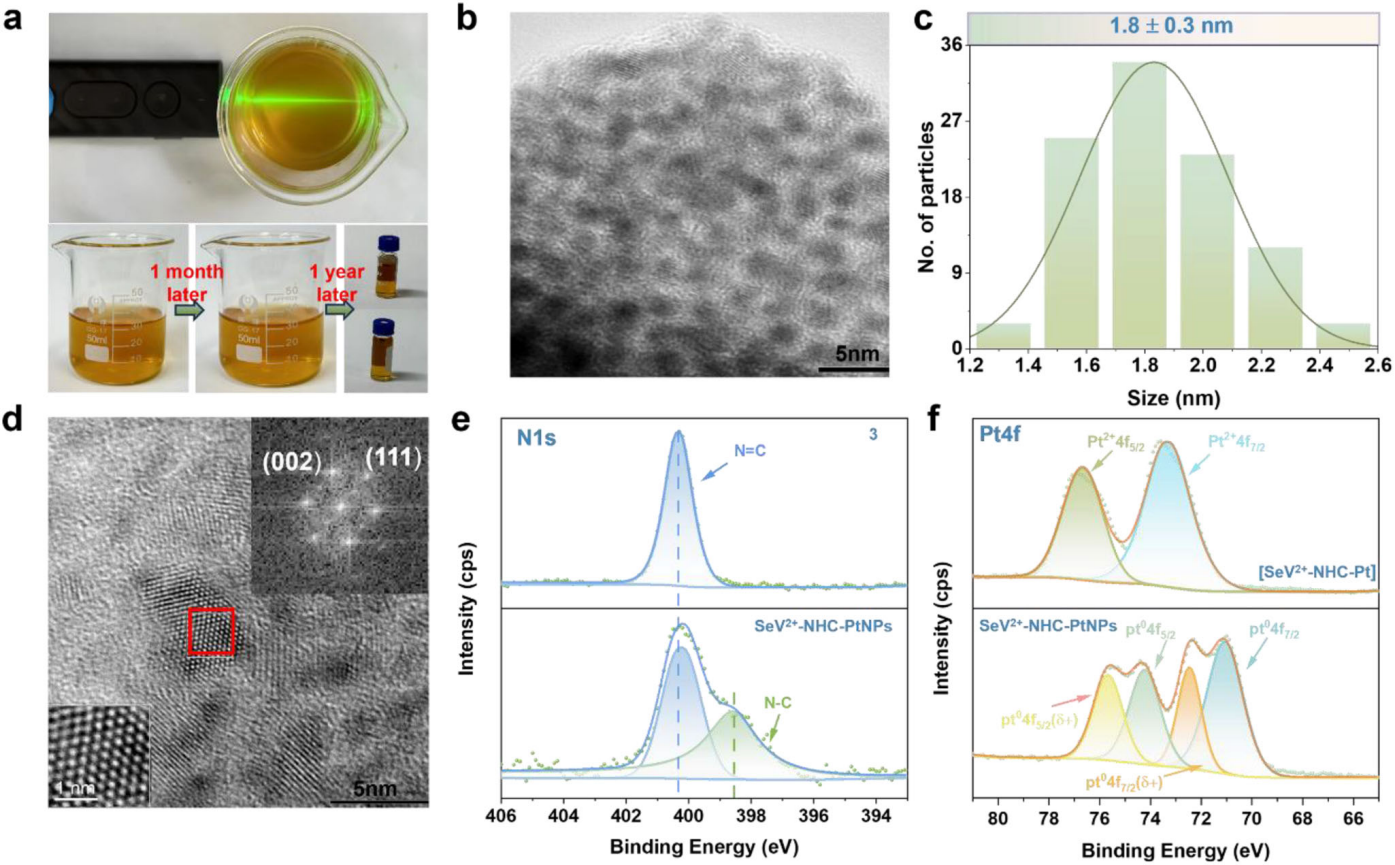
(a) Colloidal solution formed by **SeV^2+^‐NHC‐PtNPs** dissolved in water; (b) TEM image; (c) size distribution of **SeV^2+^‐NHC‐PtNPs** (measured by ImageJ software with n = 100 particles counted; data are presented as mean ± SD, calculated using Origin software); (d) HRTEM image with its corresponding FFT picture for **SeV^2+^‐NHC‐PtNPs**; (e) N 1s XPS spectra for **3** and **SeV^2+^‐NHC‐PtNPs**; (f) Pt 4f_7/2_ and 4f_5/2_ XPS spectra for **SeV^2+^‐NHC‐PtNPs** and intermediates **[SeV^2+^‐NHC‐Pt]**.

The colloidal suspension of **SeV^2+^‐NHC‐PtNPs** in water exhibited the Tyndall effect, indicating that **SeV^2+^‐NHC‐PtNPs** were uniformly distributed and highly stable in water. No agglomeration or Pt0 precipitation was observed in the aqueous solution after one month of standing. A portion of the liquid from the beaker was transferred to two small vials, and the solution remained clear even after one year (Figure 2a). Transmission electron microscopy (TEM) showed the formation of non‐agglomerated, spherical nanoparticles with fairly uniform size and diameters in the range of 1.8 ± 0.3 nm (Figure 2b,c). High‐resolution TEM (HRTEM) analyses unambiguously showed the highly crystalline character and face‐centered cubic structure of **SeV^2+^‐NHC‐PtNPs**. Fast Fourier transform (FFT) studies revealed reflections corresponding to the (111) and (002) atomic planes in all cases (Figure 2d). X‐ray photoelectron spectroscopy (XPS) has been recognized as an effective means to study the coordination of NHC ligands to the surface of metal nanoparticles (MNPs) recently [38]. Therefore, to demonstrate the coordination of NHC ligands to PtNPs, **3**, intermediates **[SeV^2+^‐NHC‐Pt]** and **SeV^2+^‐NHC‐PtNPs** were characterized using XPS (Figure 2e,f and Figures S3–S5).

The imidazolium salt **3** used as a ligand precursor shows N 1s signals at a higher binding energy (BE) of 400.2 eV due to the tight binding of electrons to these positively charged molecules. The BEs of the N 1s signals of **SeV^2+^‐NHC‐PtNPs** are 400.2 and 398.6 eV, respectively. The highest BE peak at 400.2 eV can be attributed to the imidazolium salts formed during decomposition of the organometallic precursors [39]. The BE value of the second peak (398.6 eV) is lower than that of the corresponding imidazolium salt (400.2 eV), suggesting that these water‐soluble NHC ligands can be directly coordinated to the PtNPs surface (Figure 2e) [40]. The BE of Pt 4f_7/2_ in the XPS spectra of intermediates **[SeV^2+^‐NHC‐Pt]** is 73.3 eV, corresponding to divalent platinum [41]. In the spectra of **SeV^2+^‐NHC‐PtNPs**, the same peak is significantly broadened and fitted with two peaks. The main contribution is located at 71.0 eV, characteristic of platinum in the zero‐valent state [42], but an additional contribution at 72.5 eV was necessary to fit the experimental Pt 4f_7/2_ peak correctly. This energy shift is much lower than that documented in the literature for potassium tetrachloroplatinate with platinum in the bivalent state [43] and lower than that experimentally measured for intermediates with platinum in the bivalent state (Figure 2f). It is also lower than the BE observed for the platinum oxides PtO and PtO_2_ on partially oxidized nanoparticles [44]. Previous studies have shown that organic ligands affect the Pt 4f BE of PtNPs due to the electron transfer with the PtNPs [45, 46]. This effect is observed in very fine particles with an average diameter of less than 2 nm, so that in **SeV^2+^‐NHC‐PtNPs** with an average particle size of 1.8 nm, the organic ligand affects the Pt 4f BE of PtNPs. The additional contribution of 72.5 eV can be attributed to the surface Pt atoms attached to the carbon atoms of the NHC ligand. For such ultrafine particles, **SeV^2+^‐NHC‐PtNPs** with an average size of 1.8 nm, it can be assumed that the depth of XPS detection involves the entire Pt particle from the core to the surface. A simple calculation shows that the area corresponding to the peak of platinum in the high valence state in **SeV^2+^‐NHC‐PtNPs** reaches 38% of the total area. For **SeV^2+^‐NHC‐PtNPs**, based on their diameter, it can be estimated that a particle contains 202 atoms, of which the proportion of surface atoms is about 75%. These values indicate that close to 51% of the platinum surface atoms are bound to the NHC ligand. The results of XPS show the coordination of NHC ligands to the surface of PtNPs with high coverage. (The calculation process is detailed in the Supporting Information).

The composition of **SeV^2+^‐NHC‐PtNPs** was further investigated by TGA and ATR‐FTIR (Figures S1 and S2 and Table S1). To comprehensively verify the structural integrity and the coordination of the functional ligands, the ATR‐FTIR spectra were carefully analyzed and assigned. For precursor **2**, the spectrum exhibits characteristic peaks at 1620 and 1290 cm^−1^, which are assigned to the C═N and C─N stretching vibrations of the pyridine ring, respectively, indicating the presence of the pyridine skeleton. Upon conversion to compound 3, the spectrum retains the core skeletal peaks while exhibiting new features indicative of the newly introduced imidazolium group. Specifically, a new peak emerges at 2960 cm^−1^, corresponding to the methyl C─H stretching vibration. Finally, in the spectrum of the **SeV^2+^‐NHC‐PtNPs**, the characteristic peaks representing the organic ligands (e.g., 2960, 1620, and 1440 cm^−1^) remain clearly discernible but undergo a noticeable peak broadening. This distinct peak‐broadening phenomenon is a well‐recognized hallmark of organic ligands tightly bound to the surface of metal nanoparticles via chemical coordination, which provides solid evidence for the successful construction of this hybrid single‐component nanocatalyst. The results of TGA showed that the metal content of **SeV^2+^‐NHC‐PtNPs** ranged from 26% to 29%. Based on the average metal content detected by TGA, the metal:ligand (M:Lig) ratio can be calculated to be approximately 1:1.03. However, these ratios are too high to correspond entirely to NHC ligands coordinated to the metal surface. Previous XPS analyses estimated that approximately 51% of the surface atoms in the PtNPs samples were successfully coordinated with the NHC ligand. Additionally, some of the imidazolium salt formed by hydrolysis of the NHC ligand was not removed by dialysis in the PtNPs samples. Some researchers have shown a weak interaction between this imidazolium salt and the nanoparticles, proposing a multilayer organization similar to that observed in zinc oxide NPs stabilized by alkylamine ligands [47, 48, 49]. Meanwhile, the absence of resonances of these organic residues in the ^1^H NMR spectra of **SeV^2+^‐NHC‐PtNPs** in solution confirms interactions between them and the nanoparticle surface (Figure S49). Therefore, it is believed that on the surface of PtNPs, in addition to the NHC ligands coordinated to Pt atoms, there are also some imidazole salts interacting with PtNPs, which may play a certain role in stabilizing the PtNPs.

### 2.2 Photophysical Properties

**FIGURE 3:**
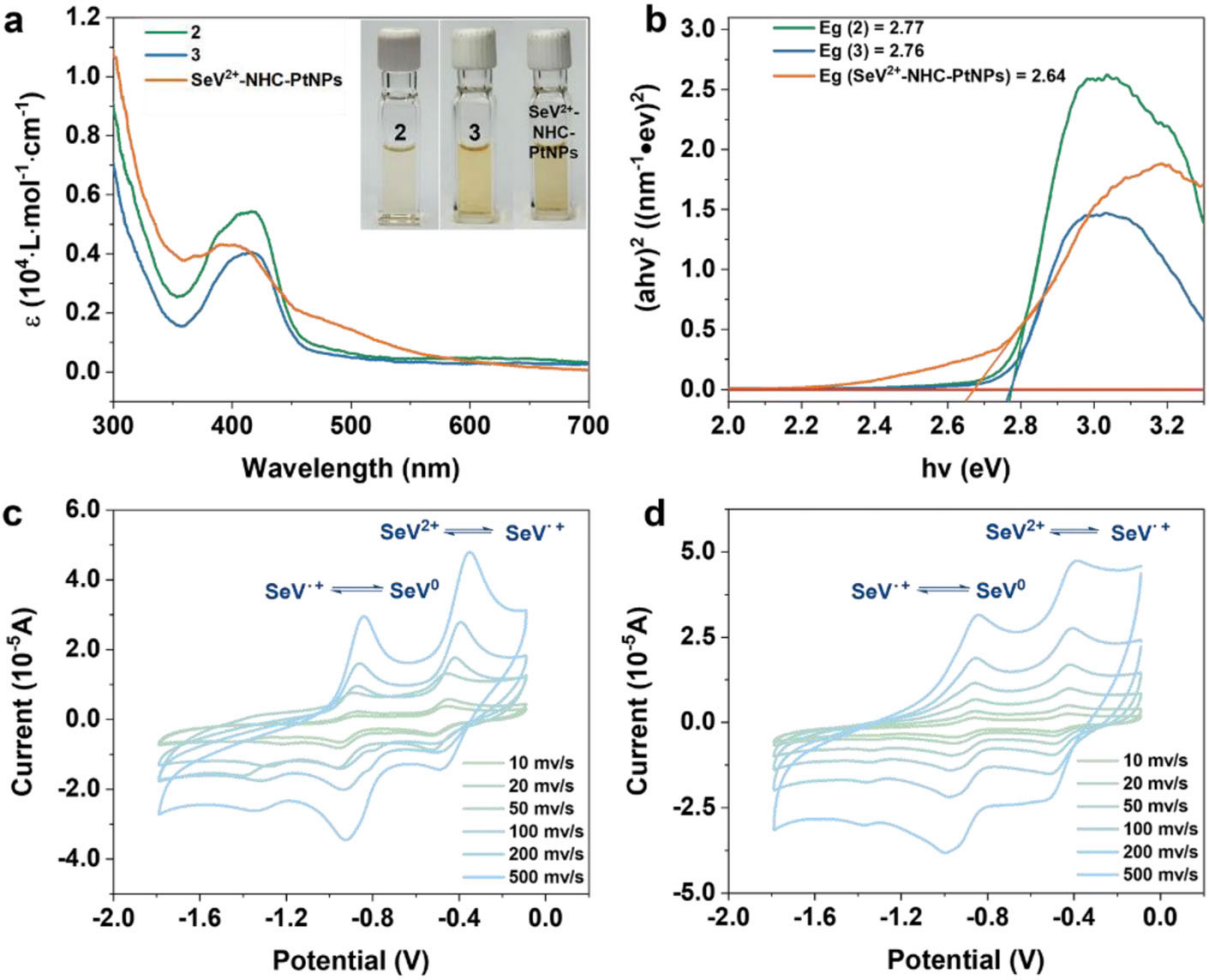
(a) UV–vis spectra of **2**, **3** and **SeV^2+^‐NHC‐PtNPs** in water, c = 10^−4^ M. The insets are photographs in aqueous solution; (b) The optical bandgap (Eg) of **2**, **3** and **SeV^2+^‐NHC‐PtNPs**; The CV of (c) **2** and (d) **3** in DMF with tetrabutylammonium hexafluorophosphate (0.1 M) as supporting electrolyte, potential E referenced to Fc/Fc^+^ (c = 10^−3^ M).

The UV–vis spectra of **2**, **3**, and **SeV^2+^‐NHC‐PtNPs** were studied to assess their light absorption properties (Figure 3a,b and Figure S6). The yellow **2** solution, the light yellow **3** solution, and the dark brown **SeV^2+^‐NHC‐PtNPs** solution all show one major absorption peak (the measured absorption maxima of **2**, **3**, and **SeV^2+^‐NHC‐PtNPs** in water solution are 416, 417, and 397 nm, respectively) (Figure 3a). Compared with **2** and **3**, **SeV^2+^‐NHC‐PtNPs** exhibit a broader absorption extending to approximately 600 nm. The optical bandgaps of **2**, **3**, and **SeV^2+^‐NHC‐PtNPs** are estimated to be 2.77, 2.76, and 2.64 eV, respectively (Figure 3b). Although the main optical bandgap of **SeV^2^^+^‐NHC‐PtNPs** remains moderate, its broad visible‐region absorption tail may arise from Pt‐related electronic transitions and interfacial charge‐transfer states mediated by Pt─C linkages. This extended absorption, together with its redox‐active SeV^2+^ moiety, suggests its potential for visible‐light‐driven photocatalysis.

### 2.3 Electrochemical Properties

The electrochemical characteristics of **2** and **3** were studied by cyclic voltammetry (CV) under different scanning speeds (Figure 3c,d and Figure S7). Both **2** and **3** showed two sets of redox peaks corresponding to two reversible one‐electron reductions, attributed to the presence of the pyridine skeleton. Since the reduction peaks of **2** (*E*_red1,1/2_ = −0.42 V, *E*_red2,1/2_ = −0.87 V, *E*_LUMO_ = −4.38 V) and **3** (*E*_red1,1/2_ = −0.42 V, *E*_red2,1/2_ = −0.86 V, *E*_LUMO_ = −4.38 V) are more positive than the reduction peaks of BnSeV^2+^(*E*_red1,1/2_ = −0.63 V, *E*_red2,1/2_ = −1.05 V, *E*_LUMO_ = −4.17 V), indicating that **2** and **3** have superior electron acceptor properties, favoring their participation in the reduction reaction (Table S2). Furthermore, the electron transfer rate constants (*k*_ET_) of **2** (*k*_ET1_ = 9.05 × 10^−3^ and *k*_ET2_ = 4.89 × 10^−3^) and **3** (*k*_ET1_ = 2.53 × 10^−3^ and *k*_ET2_ = 1.09 × 10^−2^) were calculated by CV (Figure S8 and Table S3). The results show that the electron transfer rate of compound **3** is significantly faster than that of **2** and BnSeV^2+^.

### 2.4 Density Functional Theory (DFT) Calculations

**FIGURE 4:**
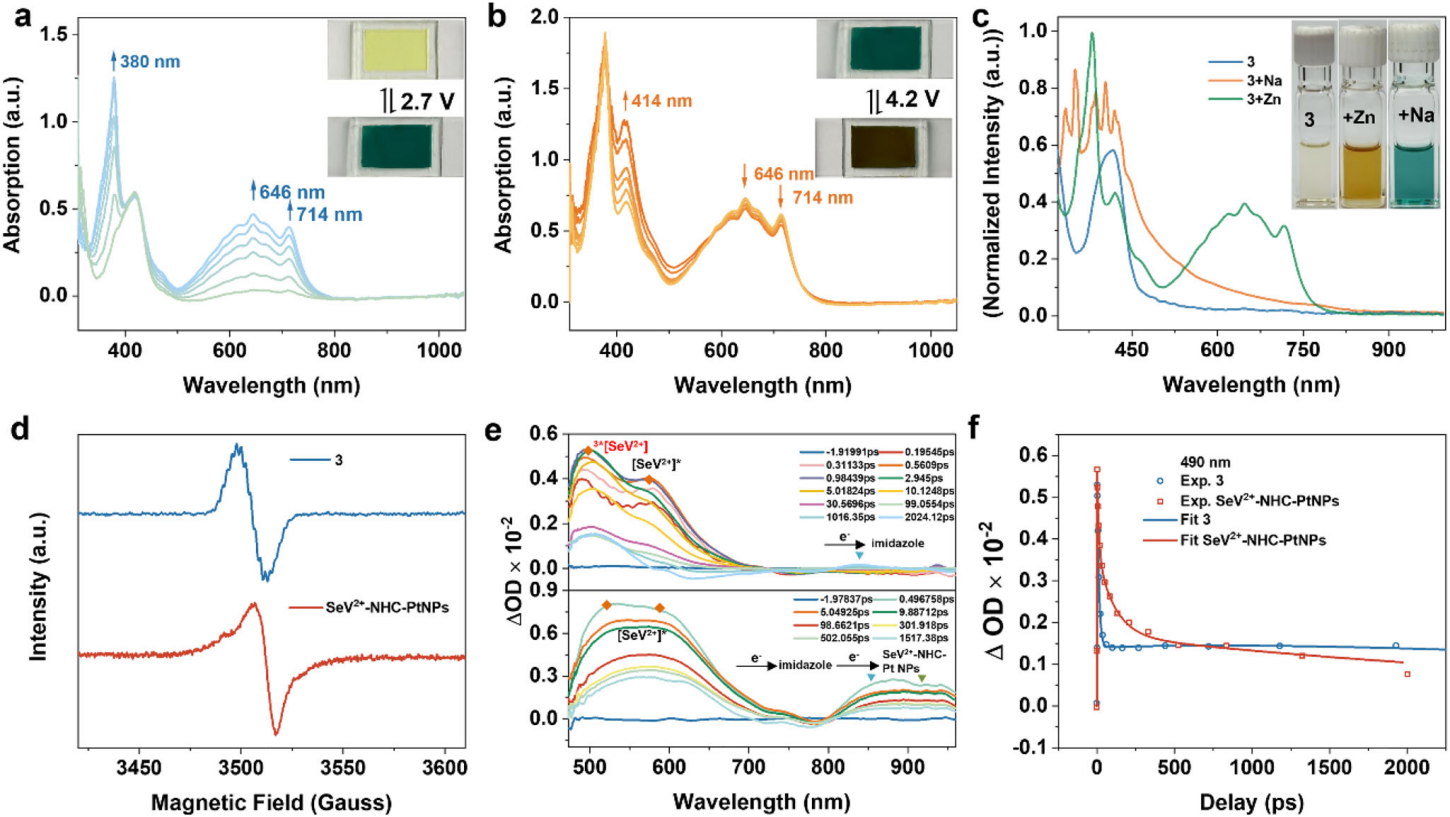
(a) Spectroelectrochemistry of **3** for the first reduction; (b) Spectroelectrochemistry of **3** for the second reduction; (c) UV–vis spectra of three redox states of **3** (c = 10^−4^ M). The inset photographs of **3** (**3**′ and **3**″) in DMF upon daylight; (d) EPR spectra of **3** and **SeV^2+^‐NHC‐PtNPs**; (e) Transient absorption spectra of **3** and **SeV^2+^‐NHC‐PtNPs** in DMSO; (f) Kinetic trajectories of **3** and **SeV^2+^‐NHC‐PtNPs** probed at 490 nm.

DFT calculations of orbital energy levels and UV‐relative absorption were performed for **3** and **SeV^2+^‐NHC‐PtNPs** to confirm the experimental results (Figures S9–S19 and Tables S4 and S5). The simulated UV spectra showed that the maximum absorption wavelengths of dication state **3**, radical cation state **3'** and neutral state **3''** were 411, 581, and 383 nm, respectively; dication state **SeV^2+^‐NHC‐PtNPs**, radical cation state **SeV^2+^‐NHC‐PtNPs'** and neutral state **SeV^2+^‐NHC‐PtNPs''** had maximum absorption wavelengths of 396, 578, and 390 nm, respectively. These three redox states were derived from the viologen skeleton, and the UV–vis absorption spectra were consistent with the calculated results. DFT calculations revealed that the introduction of PtNPs significantly affects the HOMO energy level, decreasing the energy of the HOMO energy level and reducing the energy gap. The local electrostatic potential values for **3** and **3''** are shown by color on the isosurfaces of their electron density (Figure S21). The electrostatic potential near the SeV^2+^ units drops, indicating that the SeV^2+^ units are the primary sites of the reduction. The electron spin density distributions of **3'** and **SeV^2+^‐NHC‐PtNPs'** obtained from DFT calculations are in agreement with the experimental results of electron paramagnetic resonance (EPR) (Figure 4d and Figure S20). The introduction of PtNPs does not greatly influence the stabilization of free radicals, which are stabilized away from the domain in the whole π system.

### 2.5 Electrochromism Properties

To investigate the electrochromic properties and the redox mechanisms, proof‐of‐concept electrochromic devices (ECDs) were fabricated using a DMF solution of **3** and a water solution of **SeV^2+^‐NHC‐PtNPs** as active ingredients to investigate their electrochromic properties (Figure 4a,b and Figure S22). The ECDs of **3** and **SeV^2+^‐NHC‐PtNPs** showed blue‐green and terrestrial yellow color and exhibited intense absorption across the visible region when external voltages were set at 2.7 and 4.0 V, respectively, due to the accumulation of radical states (**3'** or **SeV^2+^‐NHC‐ PtNPs'**). When the voltage was further increased to 4.2 and 4.5 V, respectively, the color of **3** and **SeV^2+^‐NHC‐PtNPs** changed to brown, with decreased absorption in the visible, indicating a transformation from radical states (**3'** or **SeV^2+^‐NHC‐PtNPs'**) to neutral species (**3''** or **SeV^2+^‐NHC‐PtNPs''**). To further understand the mechanism of electrochromism, the redox states of **3** and **SeV^2+^‐NHC‐PtNPs** in the chemical reduction reactions were investigated (Figure 4c and Figure S23). Upon addition of Zn powder to the DMF solution of **3**, the solution turns blue‐green due to the reduction of **3** to the free radical state **3'**; upon addition of Na block to the DMF solution of **3**, the solution turns brown due to the formation of the neutral species **3''** by further reduction. This agrees well with the spectroelectrochemistry and the CV experiments of **3**, indicating that the reduction process occurs in two steps. Similar experiments in an aqueous solution of **SeV^2+^‐NHC‐PtNPs** showed results consistent with the spectroelectrochemistry and CV experiments of **SeV^2+^‐NHC‐PtNPs**. EPR experiments at room temperature further verified the generation of radical states (**3'** or **SeV^2+^‐NHC‐PtNPs'**) and neutral species (**3''** or **SeV^2+^‐NHC‐PtNPs''**). As shown in Figure 4d, significant free radical signals were monitored in the **3’** and **SeV^2+^‐NHC‐PtNPs'** EPR spectra. Collectively, both the spectroelectrochemical and chemical reduction analyses confirm the excellent redox reversibility and the stability of the intermediate states in our system.

### 2.6 Transient Absorption Spectra

To study the photoexcitation and electron transfer processes of **3** and **SeV^2+^‐NHC‐PtNPs**, the fs‐TA measurements were conducted upon excitation at 410 nm (Figure 4e and Figure S24).

For the organic ligand **3**, changes in the absorbance signal were observed in the 470–900 nm range. The instantaneous increase of the characteristic peaks at 490 and 570 nm (within 0.6 ps) indicates the formation of the excited state [SeV^2+^]*, which aligns well with the electrochemical spectroscopy results. Meanwhile, at around 3 ps, alongside the rapid decay of the [SeV^2+^]* peak, increased peaks at 500–550 nm emerged, which can be assigned to the triplet charge transfer [50]. At approximately 100 ps, the isosbestic point is observed at around 800 nm, implying a change in state [51], while the increase around 850 nm is attributed to electron transfer from the SeV^2+^ to the imidazole. In stark contrast, the **SeV^2+^‐NHC‐PtNPs** exhibited ultrafast charge‐transfer dynamics across the 470–900 nm range. Within just 0.5 ps, a simultaneous and instantaneous increase in characteristic peaks at 470–700 nm and 800–960 nm was observed, with the former corresponding to the formation of the excited state [SeV^2+^]* and the latter attributed to the ultrafast IET from the excited state [SeV^2+^]* unit to the **PtNPs**.

To quantitatively evaluate the charge transfer kinetics, the transient decay traces were systematically analyzed using multi‐exponential fitting (Figure 4f and Figures S25 and S26, and Table S6). The extracted lifetimes correspond to distinct physical processes [52]: the first lifetime (τ_1_: 11.10 ps for **3** vs. 0.05 ps for **SeV^2+^‐NHC‐PtNPs**) reflects the initial excited‐state deactivation; the second lifetime (τ_2_: 1.73 ps for **3** vs. 11.49 ps for **SeV^2+^‐NHC‐PtNPs**) corresponds to the formation of spatially charge‐separated states; and the fourth lifetime (τ_4_: 6379 ps for 3 vs. 4111 ps for **SeV^2+^‐NHC‐PtNPs**) indicates the charge‐recombination process. The drastic reduction in τ to a mere 0.05 ps unambiguously demonstrates that the robust carbene‐Pt bridge opens a highly efficient new kinetic channel, enabling near‐instantaneous intramolecular electron injection from the **SeV^2+^** unit to the Pt core. Concurrently, the elongated τ_2_ signifies the successful long‐distance migration and stabilization of these electrons across the organic–inorganic interface. Furthermore, although τ_4_ slightly decreases due to the metallic nature of the Pt cocatalyst, it strictly remains within the nanosecond regime (4.1 ns). This robustly preserved, long‐lived charge‐separated state successfully retards rapid back‐electron transfer, ensuring ample time to drive the sluggish proton reduction. Collectively, this system achieves a flawless synergistic effect: ultrafast electron extraction coupled with sustained charge separation.

### 2.7 Photoelectrochemical Properties

**FIGURE 5:**
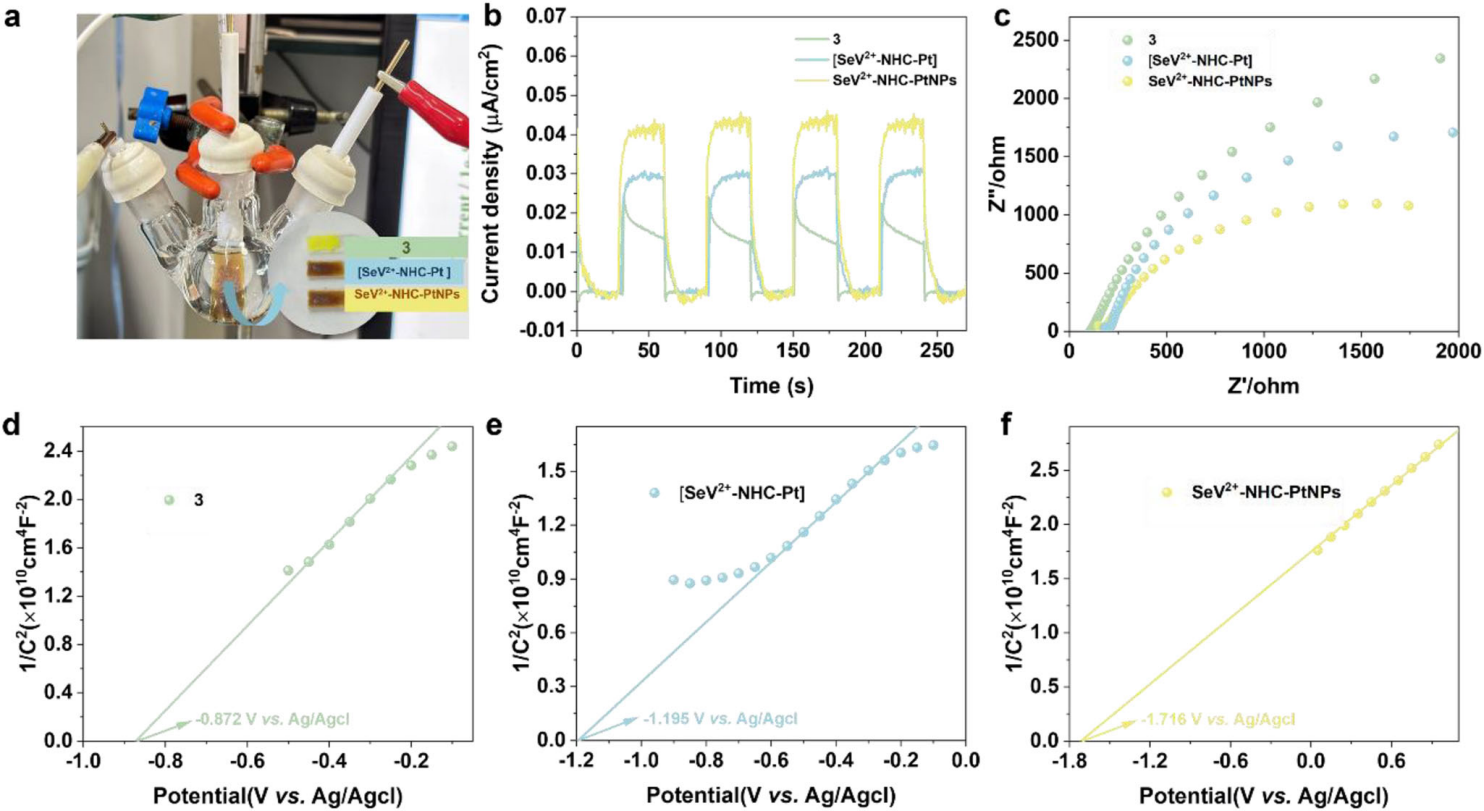
(a) Test procedure of transient photocurrent test; (b) Photocurrents and (c) EIS of **3**, **[SeV^2+^‐NHC‐Pt]** and **SeV^2+^‐NHC‐PtNPs**; Mott‐Schottky plots of (d) **3**, (e) **[SeV^2+^‐NHC‐Pt]** and (f) **SeV^2+^‐NHC‐PtNPs**.

To further investigate the charge transfer behavior, carrier excitation, and recombination dynamics of **3**, **[SeV^2+^‐NHC‐Pt]** and **SeV^2+^‐NHC‐PtNPs**, we performed transient photocurrent as well as electrochemical impedance spectroscopy (EIS) tests (Figure 5a–c). Transient photocurrent tests showed that the photocurrent increased rapidly under visible light irradiation and decreased in the absence of light, indicating a stable photocurrent response in all samples; notably, the initial anodic spike observed for SeV^2^^+^‐NHC‐PtNPs further reflects that the ultrafast initial electron injection briefly outpaces the diffusion‐limited hole‐scavenging process. Furthermore, the photocurrent densities of both sample **SeV^2+^‐NHC‐PtNPs** containing PtNPs and sample **[SeV^2+^‐NHC‐Pt]** containing divalent Pt were much greater than that of sample **3**, indicating that the introduction of the azacyclic carbene‐platinum complexes could enhance electrical conductivity and charge density, thus improving photocatalytic performance. More importantly, due to the shortened electron transfer pathway of sample **SeV^2+^‐NHC‐PtNPs**, its photocurrent density was as high as 95 µA/cm^2^, which was higher than that of sample **[SeV^2+^‐NHC‐Pt]** (45 µA/cm^2^). EIS confirmed the significant difference in conductivity among samples **3**, **[SeV^2+^‐NHC‐Pt]**, and **SeV^2+^‐NHC‐PtNPs**. Figure 5c shows the EIS for these three electrodes, with all Nyquist plot impedance spectra exhibiting similar characteristics at open circuit potential conditions. A non‐linear regression fit using the conventional Randles circuit (R (QR)) procedure gives an active charge transfer resistance of **SeV^2+^‐NHC‐PtNPs** < **[SeV^2+^‐NHC‐Pt]** < **3** (Figure S27). This indicates a lower charge transfer resistance and faster charge transfer rate due to the introduction of the superior electron transfer and electron acceptance characteristics of Pt. In addition, to clarify the energy band structure of the samples, their flat band potentials were measured using Mott‐Schottky curves, obtained by fitting the x‐intercept of the linear region at C^−2^ = 0 (Figure 5d–f). The slopes of the tangent lines are all positive, determining that samples **3**, **[SeV^2+^‐NHC‐Pt]**, **SeV^2+^‐NHC‐PtNPs** all exhibit n‐type semiconductor characteristics. According to the Mott–Schottky equation, the *E*_fb_ of vs. Ag/AgCl for samples **3**, **[SeV^2+^‐NHC‐Pt]**, **SeV^2+^‐NHC‐PtNPs** were −0.872, −1.195, and −1.716 V respectively, which were further converted to vs. normal hydrogen electrode (NHE) according to the equation *E*_NHE_ = *E*_Ag/AgCl_ + 0.197 V. For n‐type semiconductors, the flat‐band potential is typically positive 0.1–0.3 V compared to the conduction‐band potential. Therefore, the E_CB_ of samples **3**, **[SeV^2+^‐NHC‐Pt]**, and **SeV^2+^‐NHC‐PtNPs** were approximately −0.775, −1.098, and −1.619 V (vs. NHE), respectively. Notably, compared to **3** and **[SeV^2+^‐NHC‐Pt]**, **SeV^2+^‐NHC‐PtNPs** result in a significant negative shift of the E_CB_ due to coordination with Pt, providing a massive overpotential (>1.3 V at pH 5.0) that greatly benefits the thermodynamic driving force of photocatalytic proton reduction (e.g., hydrogen precipitation) [53].

### 2.8 Visible‐Light‐Driven Hydrogen Production

**FIGURE 6:**
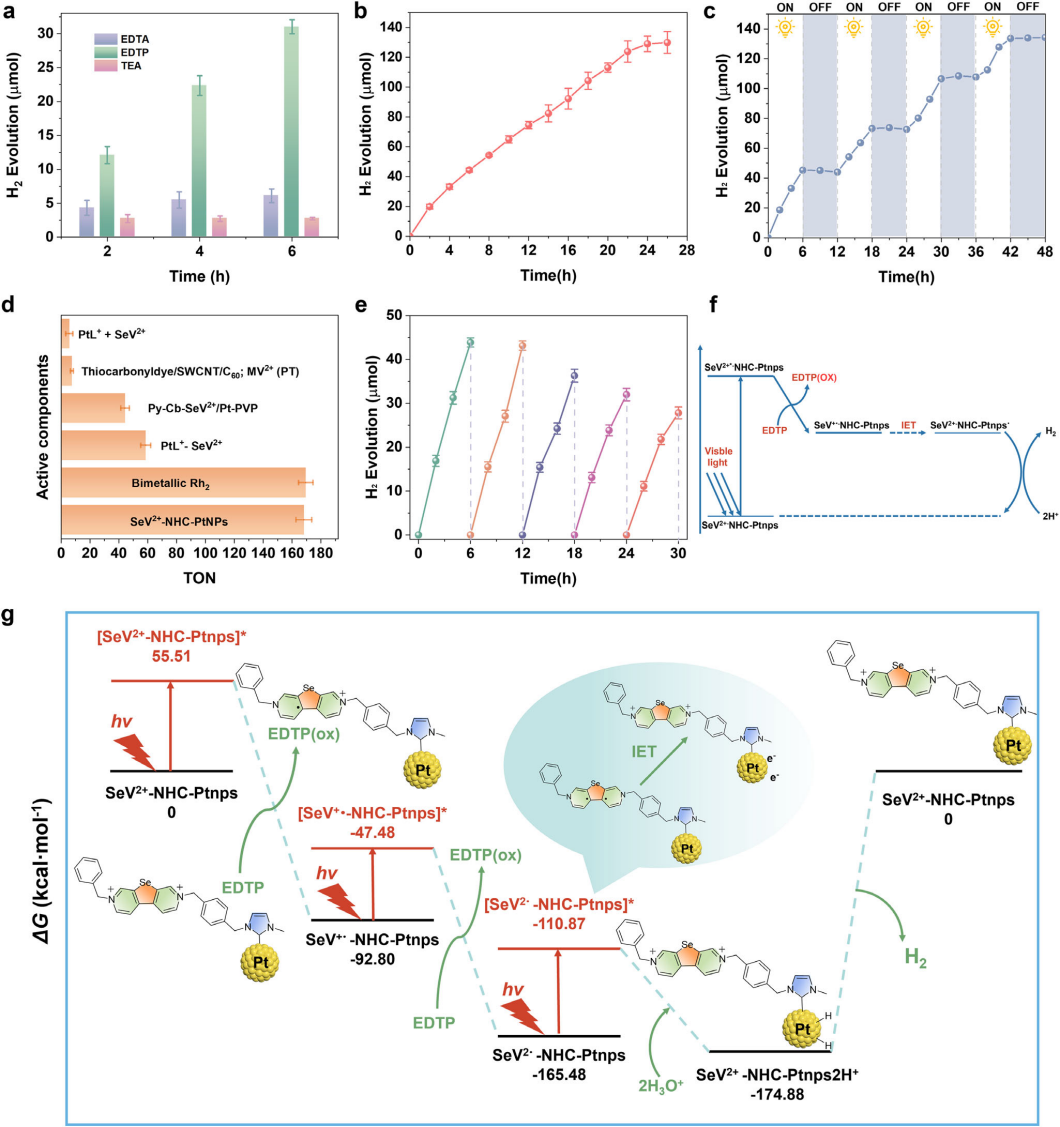
(a) The screening of the sacrificial agent (0.15 mM) used as catalyst **SeV^2+^‐NHC‐PtNPs**; (b) Time‐dependent H_2_ evolution using EDTP; (c) Photo‐responsive tests for **SeV^2+^‐NHC‐PtNPs**; (d) Comparison of hydrogen evolution volume of different composites materials; (e) Reusability tests; (For a, b, d, and e, independent experiments were performed in triplicate (n = 3). Data are presented as mean ± SD, and the error bars represent the standard deviation.) (f) Schematic of charge transfer processes and (g) Gibbs free energy calculation of visible‐light‐driven hydrogen evolution process.

A single‐component visible‐light‐driven hydrogen production system has been designed using **SeV^2+^‐NHC‐PtNPs** due to its strong absorption of visible light, easy formation of free radicals, excellent photoelectrochemical properties, and efficient IET process. In this system, **SeV^2+^‐NHC‐PtNPs**, which are highly soluble in water, serve as a photosensitizer, an electronic medium, and a catalyst. Pre‐experiments were conducted to screen the sacrificial agents; the photocatalytic hydrogen production of the system was compared when different sacrificial agents were used under the same conditions (Figure 6a). The results showed that the system with an excess of sacrificial agent *N,N,N’,N’*‐ethylenediamine tetrakis (methylene‐phosphonic acid) (EDTP) exhibited the highest hydrogen production, which could be attributed to the lower pH of the EDTP system favoring proton reduction [54, 55].

In the mechanism proposed in Figure 6f,g, the ligand portion of SeV^2+^‐NHC, which acted as a photosensitizer and an electron mediator, was first excited under visible light to produce the excited state SeV^2+^*‐NHC. In this state, SeV^2+^*‐NHC accepted an electron from the sacrificial agent donor EDTP and transformed into SeV^+^‐NHC. The electron then underwent IET through the NHC─Pt bond from the ligand portion to the PtNPs, and SeV^+^‐NHC was simultaneously oxidized to SeV^2+^‐NHC. Then, the photoelectrons gathered on the surface of the PtNPs to reduce H^+^ to H_2_, completing the catalytic cycle.

Then the system containing a mixture of **SeV^2+^‐NHC‐PtNPs** (0.02 µmol), EDTP (0.15 mmol), 0.1 M NaCl and 10 mL 0.1 M acetate buffer solution was sealed in a Pyrex bottle. After bubbling with argon for 30 min, the bottle was exposed to a PLS‐SXE300D xenon lamp with a filter (λ >400 nm) at 100 mW/cm^2^. H_2_ was incrementally released within 24 h, as calculated from the standard curve (Figures S28 and S29). In the system, the total hydrogen production of **SeV^2+^‐NHC‐PtNPs** was 129.89 µmol, an evolution rate of 2706 µmol·h^−1^·g^−1^, an apparent quantum yield (AQY) of 0.9% and a turnover number (TON) of 169 (Figure 6b). To place the catalytic performance into the right perspective, a comprehensive comparison of the TONs, hydrogen yield, and rate of hydrogen production was conducted against representative state‐of‐the‐art photocatalytic architectures (Figure 6d and Table S7). First, our single‐component system significantly outperforms many multi‐component systems (such as Thiocarbonyldye/SWCNT/C_60_/MV^2+^, g‐C_3_N_4_/pTA‐o‐TPV^2+^/1 wt.% Pt, MPor‐DETH‐COFs/8 wt.% H_2_PtCl_6_ and Py‐Cb‐SeV^2+^/Pt‐PVP) that rely on electrostatic attraction. Second, compared to previous single‐component works, our system exhibits highly competitive performance, vastly surpassing the precursor PtL‐SeV^2+^. Although a reported single‐molecule catalyst achieved a similar TON under harsh conditions like strong acids and organic media [56], our **SeV^2+^‐NHC‐PtNPs** operate efficiently in mild aqueous buffers. Figure 6c illustrates the photo‐response characteristics of **SeV^2+^‐NHC‐PtNPs** when catalyzing hydrogen production in the system under the same conditions. It can be seen that when the light irradiation is switched on, the hydrogen production in the system increases rapidly and steadily; when the light irradiation is switched off, the hydrogen production remains more or less constant or decreases slightly (the total amount of gas decreases due to the constant extraction of gas). This indicates that **SeV^2+^‐NHC‐PtNPs** can only catalyze the production of hydrogen under light irradiation conditions. In addition to good hydrogen production properties, the catalyst should also have good durability for practical use in production. This system was therefore repeated five times under the same experimental conditions for hydrogen production from photolytic water (Figure 6e). The results showed that the hydrogen production of the system after the fifth cycle was 63.4% of that of the first cycle. Considering that no additional sacrificial agent was replenished during the 30 h test and the system exhibited no macroscopic catalyst agglomeration (Figure S30), this apparent activity decline is primarily attributed to the depletion of EDTP and the reduction of the active surface area of the nanoparticles caused by the accumulation of their oxidation by‐products.

## 3 Conclusion

In summary, hydrophilic SeV^2^^+^‐NHC‐PtNPs were successfully synthesized via a thermally induced reduction method. Quantitative surface analyses revealed a high coordination coverage (51%) of NHC ligands on the Pt surface, which, alongside weak interactions from residual imidazolium salts, synergistically contributed to the exceptional structural stabilization of the nanoparticles. The covalently integrated architecture exhibited broadened visible‐light absorptivity (370–600 nm) while well‐preserving its intrinsic electron acceptance properties. Importantly, comprehensive spectroscopic and photoelectrochemical investigations demonstrated that the robust NHC‐Pt linkages not only lower the interfacial charge‐transfer resistance but also facilitate highly efficient and directional intramolecular electron transfer (IET). Consequently, this system seamlessly integrates the roles of a photosensitizer, electron mediator, and catalyst, achieving a remarkable visible‐light‐driven H_2_ evolution rate of 2706 µmol·h^−1^·g^−1^, a TON of 169, and an AQY of 0.9% in aqueous solution. These results underscore the potential of SeV^2^^+^‐NHC‐PtNPs as a simplified and highly efficient system for solar energy conversion.

## 4 Experimental Section

### 4.1 Materials

All operations were carried out using standard Schlenk tube and glovebox (Vigor) techniques under an argon atmosphere. Tetrabutylammonium chloride (n‐Bu_4_NCl, 98%) was purchased from TCI Chemical Inc. *N*‐methylimidazole (99%), 1,4‐bis(bromomethyl)benzene (97%), potassium tetrachloroplatinate(II) (K_2_PtCl_4_, 99%), ammonium hexafluorophosphate (NH_4_PF_6_, 98%), sodium tert‐butoxide (t‐BuONa, 98%), dimethyl sulfoxide (DMSO, 99.7%, extra dry), acetonitrile (CH_3_CN, 99.9%, extra dry) and N,*N‐*dimethylformamide (DMF, 99.8%, Extra Dry) were purchased from Energy Chemical Inc. Monosubstituted benzyl selenoviologen [57] and cis‐dimethylbis(dimethyl sulfoxide)platinum(II) [58] were synthesized as described in the literature. Other reagents and solvents were used as commercially available without further purification. Deionized water (type II quality) was obtained using a Millipore Elix 10 UV Water Purification System. Dialysis tubing cellulose membrane (average flat width 44 mm, molecular weight cut‐off = 14000 Dalton) was purchased from Beyotime Biotech. Inc.

### 4.2 Materials Characterization

NMR spectra were measured on a Bruker Avance‐400 and Avance‐III HD 600 MHz spectrometer in the solvents indicated; chemical shifts are reported in units (ppm) by assigning the TMS resonance in the ^1^H spectrum as 0.00 ppm, the DMSO‐*d*_6_ resonance in the ^13^C spectrum as 39.50 ppm. Coupling constants are reported in Hz with multiplicities denoted as *s* (singlet), *d* (doublet), *t* (triplet), *q* (quartet), and *m* (multiplet). TEM and HRTEM observations were carried out with a JEOL JEM‐2100Plus electron microscope, working at 200 kV with a resolution of 1.94 Å. UV–vis measurements were performed using a DH‐2000‐BAL Scan spectrophotometer. The CV in solution was measured using CHI660E B157216, with a polished gold electrode as the working electrode, a Pt‐net as the counter electrode, and an Ag wire as the reference electrode, using ferrocene/ferrocenium (Fc/Fc^+^) as the internal standard. EPR was measured using a Bruker A300‐9.5/12 instrument at room temperature in dry degassed DMF and dry degassed DMSO. The EPR parameters for the experiments are as follows: modulation frequency = 100 kHz, modulation amplitude = 8.0 G, time constant = 82 ms, conversion time = 80 ms, center field = 3518 G, sweep width = 400 G, microwave attenuation = 30 dB, microwave power = 0.2 mW. TGA measurements were carried out in the temperature range of 35°C–800°C by using a METTLER TOLEDO TGA/DSC3 thermal analyzer in air, at a heating rate of 10 K min^−1^. HRMS data were collected on a WATERS I‐Class VION IMS QT mass spectrometer in ESI positive mode. Analytical gas chromatography (GC) for gas samples was carried out on a SHIMDZU GC‐2014ATF/SPL (TDX‐01 60/80 mesh, 2.0 mm × 3.2 mm × 2.1 mm‐FID, TCD permanent gases, N_2_ carrier gas). Nitrogen is the carrier gas. The 300 W xenon lamp (PLS‐SXE300D) used for irradiation was supplied by Beijing Perfectlight Technology Co., Ltd. The illumination intensity was measured using an FZ‐A irradiatometer from Beijing Normal University Optoelectronic Technology Co., Ltd. XPS was collected by a Thermo Fisher ESCALAB Xi^+^ type X‐ray photoelectron spectrometer. FTIR was measured using a Nicolet iS10. Photographs were taken using a Nikon D5100 digital camera.

### 4.3 Synthesis of *cis*‐[PtMe_2_(DMSO)_2_]

Dissolve potassium chloroplatinate (415 mg, 1.0 mmol) in water (5 mL), then add DMSO (88 mg, 1.1 mmol), stir for 2 min, stand until crystals are separated, and wash with water, EtOH and Et_2_O (5 mL × 3). After drying, cis‐PtCl_2_(DMSO)_2_ is obtained (126 mg, 30%). A solution of cis‐PtCl_2_(DMSO)_2_ (447 mg) in DMSO (4 cm^3^) was treated with SnMe_4_ (0.3 cm^3^) and the solution was maintained at 70°C for 24 h. Evaporation of solvent at 70°C gave a white solid which was washed with diethyl ether and dissolved in CH_2_Cl_2_. The solution was then treated with charcoal and filtered. Dry the filtrate to obtain a white solid, which is the product (267 mg, 66%).

### 4.4 Synthesis of 2

A solution of **1** in dried and degassed DMF (15 mL) was added dropwise to a 50 mL pressure pipe containing another solution of 1,4‐bis(bromomethyl)benzene (762 mg, 2.885 mmol) in the same solvent (5 mL) under an argon atmosphere. The resulting yellow solution was stirred at 70°C for 3 days. After this period of time, the precipitate was filtered, washed with DMF (3 × 20 mL), and dried under vacuum (5 h, 70°C, 10 mbar). The solid obtained was dispersed in H_2_O (10 mL), and saturated NH_4_PF_6_ solution (5 mL) was dropped into the dispersion while stirring. After 8 h of reaction at room temperature, the precipitate was isolated via vacuum filtration and washed with H_2_O (3 × 10 mL). The light yellow solid **2** was obtained by column chromatography after vacuum drying (1260 mg, 61%).

### 4.5 Synthesis of 3

A solution of **2** (1260 mg, 1.578 mmol) in dried and degassed acetonitrile (10 mL) was added dropwise to a 25 mL pressure‐resistant tube containing another solution of *N‐*methylimidazole (189 µL, 2.368 mmol) in the same solvent (2 mL) under an argon atmosphere. The resulting yellow solution was stirred at 70°C for 3 days. After this period of time, the precipitate was filtered, washed with acetonitrile (3 × 20 mL), and dried under vacuum (5 h, 70°C, 10 mbar). The solid obtained was dispersed in H_2_O (10 mL), and saturated NH_4_PF_6_ solution (5 mL) was dropped into the dispersion while stirring. After reacting for 12 h at room temperature, the precipitate was isolated via vacuum filtration, washed with H_2_O (3 × 10 mL), and dried under vacuum (24 h, 70°C, 10 mbar). The resulting light‐yellow solid was then dispersed in acetonitrile (10 mL), and a saturated acetonitrile solution of tetrabutylammonium chloride (5 mL) was added dropwise to the dispersion under stirring. After 6 h of reaction at room temperature, the precipitate was isolated by vacuum filtration, washed with acetonitrile (3 × 10 mL) and dried under vacuum to obtain **3** (260 mg, 18%).

### 4.6 Synthesis of SeV^2+^‐NHC‐PtNPs

To a solution of *cis*‐[PtMe_2_(DMSO)_2_] (161 mg, 0.42 mmol) and **3** (260 mg, 0.42 mmol) in DMSO (10 mL) was added sodium tert‐butoxide (40.36 mg, 0.42 mmol). The mixture was stirred at room temperature for 1 h and filtered through a diatomaceous earth plug. After complete evaporation of the solvent (70°C, 4 mbar), the resulting reddish‐brown solid was introduced into a 10 mL pressure‐resistant tube and dissolved in deionized water (5 mL). The resulting reddish‐brown liquid was stirred at 1000 rpm and heated at 80°C for 18 h. After this time, the black solution was allowed to slowly reach room temperature and then filtered through a PTFE 0.2 µm filter to remove any particles from the suspension. The obtained solution was dialyzed using a cellulose membrane (MWCT = 14 000 Dalton) for 36 h. The solvent was removed under vacuum (3 h, 50°C, 100 mbar) to obtain a black solid, which was dried under vacuum (25°C, 10 mbar) overnight. The black powder obtained was platinum nanoparticles **SeV^2+^‐NHC‐PtNPs** (62 mg, 18%).

### 4.7 EDTP‐Triggered H_2_ Production

The EDTP‐triggered H_2_ production process is shown in Figure 6b. The system containing a mixture of **SeV^2+^‐NHC‐PtNPs** (0.02 µmol), EDTP (0.15 mmol), and 10 mL 0.1 M acetate buffer solution (0.03 M CH_3_COOH and 0.07 M CH_3_COONa, pH = 5.0) was sealed in a 20 mL Pyrex bottle. After bubbling with argon for 30 min away from light, 200 µL CH_4_ was injected, and the bottle was exposed to the PLS‐SXE300D xenon lamp with a filter (λ >400 nm, purchased from Beijing Perfectlight Technology Co., Ltd) at 100 mW. Then 200 µL of the upper gas of the reactor was injected into the gas chromatography per 2 h to measure H_2_ evolution. The production of the hydrogen was calculated according to the H_2_ normalized curve.

### 4.8 The Repeatability of H_2_ Production

The repeatability of H_2_ production process is shown in Figure 6e. The mixture of **SeV^2+^‐NHC‐PtNPs** (0.02 µmol), EDTP (0.15 mmol), and 10 mL 0.1 M acetate buffer solution (0.03 M CH_3_COOH and 0.07 M CH_3_COONa, pH = 5.0) was bubbled with argon for 30 min away from light, and irradiated under 100 mW xenon lamp, 200 µL CH_4_ was injected. Then, 200 µL of gas was measured by GC every 2 h. To avoid mass‐loss errors during traditional solid recovery of the fully water‐soluble catalyst, an in situ cycling protocol was employed. After 6 h, without separating the nanoparticles, the system was directly bubbled with argon for 30 min to completely evacuate the generated H_2_ and CH_4_. Once restored to a hydrogen‐free state, the next cycle was immediately initiated.

### 4.9 Photoresponsiveness of H_2_ Production

The photoresponsiveness of the H_2_ production process is shown in Figure 6c. The mixture of **SeV^2+^‐NHC‐PtNPs** (0.02 µmol), EDTP (0.15 mmol), and 10 mL 0.1 M acetate buffer solution (0.03 M CH_3_COOH and 0.07 M CH_3_COONa, pH = 5.0) was bubbled with argon for 30 min away from light, and irradiated under 100 mW xenon lamp, 200 µL CH_4_ was injected. Then 200 µL of gas was measured by GC per 2 h. After 6 h, the reaction flask was protected from light for 6 h and then tested for H_2_ production under dark conditions, repeating the procedure four times.

### 4.10 Statistical Analysis

Standard data pre‐processing was conducted by verifying raw data integrity; no specific mathematical transformations or normalizations were applied to the original datasets. Unless otherwise stated, data are presented as the mean ± standard deviation (SD). For the quantitative evaluation of nanoparticle size distribution (Figure 2), the sample size was n = 100 particles, measured from TEM images using ImageJ software. For the photocatalytic performance tests (Figure 6), all experiments were performed in independent triplicates (n = 3). Descriptive statistics, including the calculation of mean values and standard deviations for error bars, were performed using the Statistics tool (Descriptive Statistics function) in Origin software.

## Author Contributions

**Wenxi He**: conceptualization, data curation, formal analysis, investigation, software, visualization, writing – original draft, writing – review and editing. **Chenjing Liu**: software, writing – review and editing. **Gang He**: conceptualization, funding acquisition, project administration, writing – review and editing, resources, supervision, validation. **Wenxin Wei**: writing – review and editing. **Hairui Lei**: formal analysis, writing – review and editing. **Ni Yan**: writing – review and editing, resources. **Guoping Li**: conceptualization, formal analysis, funding acquisition, project administration, resources, supervision, writing – review and editing, methodology. **Yawen Li**: writing – review and editing. **Yu Liu**: software, resources.

## Funding

This research was supported by the National Natural Science Foundation of China (22205172, 22175138, 22201228, and 52203240), the Young Talent Fund of Association for Science and Technology in Shaanxi (20220604 and 20230624), the China National Postdoctoral Program for Innovative Talents (BX2021231), the China Postdoctoral Science Foundation (2022M712497 and 2022M712530), Shaanxi Province Technological Innovation Guidance Special (2024ZC‐YYDY‐96) and the Key Research and Development Program of Shaanxi Province (2025CY‐JJQ‐125 and Critical Core Technology Project 2024CY‐GJHX‐02).

## Conflicts of Interest

The authors declare no conflicts of interest.

## Supporting information




**Supporting File**: advs76777‐sup‐0001‐SuppMat.pdf.

## Data Availability

The data that support the findings of this study are available from the corresponding author upon reasonable request.
