# Supplementary material for: Covalently Integrated Selenoviologen–N‐Heterocyclic Carbene–Pt Nanoparticles for Visible‐Light‐Driven Hydrogen Evolution
Source: Adv Sci (Weinh). 2026 Jul 21:e76777. Online ahead of print. doi: 10.1002/advs.76777 (PMC13387029; doi:10.1002/advs.76777)
Supplement: Supplementary file 1 — Supporting File: advs76777‐sup‐0001‐SuppMat.pdf. [file ADVS-9999-e76777-s001.pdf]

Supporting Information

Covalently Integrated Selenoviologen–N-Heterocyclic Carbene–Pt Nanoparticles for Visible-Light-Driven Hydrogen Evolution

Wenxi He,[1] Guoping Li,[1,2] Chenjing Liu,[1] Yu Liu,[3] Yawen Li,[1] Wenxin Wei,[1] Hairui Lei,[4] Ni Yan,[5] and Gang He[1,2*]*

1. W. He, G. Li, C. Liu, Y. Li, W. Wei, G. He
Frontier Institute of Science and Technology, Interdisciplinary Research Center of Frontier Science and Technology, State Key Laboratory for Strength and Vibration of Mechanical Structures, Institute of New Concept Sensors and Molecular Materials, Shaanxi Key Laboratory of New Conceptual Sensors and Molecular Materials, Engineering Research Center of Key Materials for Efficient Utilization of Clean Energy of Shaanxi Province, Xi'an Key Laboratory of Electronic Devices and Material Chemistry, Xi'an Jiaotong University, Xi'an, Shaanxi Province, 710054, P. R. China
E-mail: ligp16@xjtu.edu.cn, ganghe@mail.xjtu.edu.cn

2. G. Li, G. He
Shengzhou Yangtze River Delta Institute for the Integration of Industry and Education in New Energy, Shengzhou, Zhejiang Province, 312400, P. R. China

3. Y. Liu
Shaanxi Qinzhou Nuclear and Radiation Safety Technology Co., Ltd, Xi'an, Shaanxi Province, 710000, P. R. China

4. H. Lei
College of Energy Materials and Chemistry, Inner Mongolia University, Hohhot, China

5. N. Yan
School of Materials Science & Engineering, Chang'an University, Xi'an, Shaanxi Province, 710064, P. R. China

Contents

1. Materials and instrumentation 3
2. Experimental conditions 4
   2.1 HRTEM and TEM 4
   2.2 The fabrication of electrochromic devices (ECD) and the electrochemical spectra 5
   2.3 Computational calculations using the Gaussian 09 code 5
   2.4 Femtosecond transient absorption (fs-TA) measurements 5
   2.5 EDTP-triggered H_2 production 6
   2.6 The repeatability of H_2 production 7
   2.7 Photoresponsiveness of H_2 production 7
3. Synthetic procedures 7
   3.1 Synthesis of ***cis*-[PtMe$_2$(DMSO)$_2$]** 8
   3.2 Synthesis of **2** 8
   3.3 Synthesis of **3** 9
   3.4 Synthesis of **SeV^{2+}-NHC-PtNPs** 10
4. Theoretical Calculations 11
   4.1 Calculation of the total number of platinum atoms in the **SeV^{2+}-NHC-PtNP** 11
   4.2 Calculation of the coverage of organic ligands on the surface of **SeV^{2+}-NHC-PtNPs** 11
   4.3 Calculation of the Metal : Ligand (M:Lig) Ratio on the Surface of the **SeV^{2+}-NHC-PtNP** 13
   4.4 Calculation of the relative molecular mass of the **SeV^{2+}-NHC-PtNP** 14
5. ATR-FTIR spectra of the imidazolium salts and Pt nanoparticles 15
6. TGA analysis 16
7. The XPS spectra 17
8. Optical characterization data 20
9. The cyclic voltammogram 21
10. Evaluation of electron-transfer constant k_{ET} 22
11. Computed UV/vis spectra 23
12. Calculated spin density plots for the radical species 31
13. Electrostatic potential surfaces 31
14. Electrochromism of **SeV^{2+}-NHC-PtNPs** 32
15. UV-vis spectra of two redox states of **SeV^{2+}-NHC-PtNPs** 32
16. Femtosecond transient absorption measurements 33
17. H_2 normalized curve 36
18. Hydrogen evolution under Xenon lamp 38
19. Cartesian coordinates of optimized structures studied in this work 53
20. ^{1}H, ^{13}C, and high-resolution mass spectrum (HRMS) 68
21. Reference 74

1. Materials and instrumentation

All operations were carried out using standard Schlenk tube and glovebox (Vigor) techniques under argon atmosphere. Tetrabutyl ammonium chloride (n-Bu_4NCl, 98%) was purchased from TCI Chemical Inc. *N*-methylimidazole (99%), 1,4-bis(bromomethyl)benzene (97%), potassium tetrachloroplatinate(II) (K_2PtCl_4, 99%), ammonium hexafluorophosphate (NH_4PF_6, 98%), sodium tert-butoxide (t-BuONa, 98%), dimethyl sulfoxide (DMSO, 99.7%, extra dry), acetonitrile (CH_3CN, 99.9%, extra dry) and N,N-dimethylformamide (DMF, 99.8%, Extra Dry) were purchased from Energy Chemical Inc. Monosubstituted benzyl selenoviologen[1] and cis-dimethylbis(dimethyl sulfoxide)platinum(II)[2] were synthesized as described in the literature. Other reagents and solvents were used as commercially available without further purification. Deionized water (type II quality) was obtained using a Millipore Elix 10 UV Water Purification System. Dialysis tubing cellulose membrane (average flat width 44 mm, molecular weight cut-off = 14000 Dalton) was purchased from Beyotime Biotech. Inc. NMR spectra were measured on a Bruker Avance-400 and Avance-III HD 600 MHz spectrometer in the solvents indicated; chemical shifts are reported in units (ppm) by assigning TMS resonance in the ^{1}H spectrum as 0.00 ppm, DMSO-d_6 resonance in the ^{13}C spectrum as 39.50 ppm. Coupling constants are reported in Hz with multiplicities denoted as s (singlet), d (doublet), t (triplet), q (quartet) and m (multiplet). TEM and HRTEM observations were carried out with a JEOL JEM-2100Plus electron microscope, working at 200 kV with a resolution point of 1.94 Å. UV-vis measurements were performed using DH-2000-BAL Scan spectrophotometer. The cyclic voltammetry (CV) in solution were measured using CHI660E B157216, with a polished gold electrode as the working electrode, a Pt-net as

counter electrode, and an Ag wire as reference electrode, using ferrocene/ferrocenium (Fc/Fc^+) as internal standard. EPR was measured using a Bruker A300-9.5/12 instrument at room temperature in dry degassed DMF and dry degassed DMSO. The EPR parameters for the experiments are as follows: modulation frequency = 100 kHz, modulation amplitude = 8.0 G, time constant = 82 ms, conversion time = 80 ms, center field = 3518 G, sweep width = 400 G, microwave attenuation = 30dB, microwave power = 0.2 mW. TGA measurements were carried out in the temperature range of 35-800 °C by using of a METTLER TOLEDO TGA/DSC3 thermal analyzer in air, at a heating rate of 10 K•min^{-1}. HRMS were collected on a WATERS I-Class VION IMS QT of mass spectrometer in an ESI positive mode. Analytical gas chromatography (GC) for gas sample were carried out on a SHIMDZU GC-2014ATF/SPL (TDX-01 60/80 mesh, 2.0 mm × 3.2 mm × 2.1 mm-FID, TCD permanent gases, N_2 carrier gas). Nitrogen as the carrier gas. The 300 W xenon lamp (PLS-SXE300D) used for irradiation was supplied by Beijing Perfectlight Technology Co., Ltd.. The illumination intensity was probed by FZ-A irradiatometer from Beijing Normal University Optoelectronic Technology Co., Ltd.. XPS was collected by thermo Fisher ESCALAB Xi^+ type X-ray photoelectron spectrometer. FTIR were measured using Nicolet iS10. Photographs were taken using a Nikon D5100 digital camera.

2. Experimental conditions

2.1 HRTEM and TEM

Samples for TEM and HRTEM were prepared by deposition after evaporation on a covered holey copper grid of three drops of the nanoparticles in water (0.5 mL) dispersed in EtOH (2 mL),or a drop of the crude aqueous colloidal solution. The approximation of the particles mean size was made through a manual analysis of enlarged micrographs by

measuring a large number of particles (≥ 100) using ImageJ software to analyse the images. FFT treatments have been carried out with Digital Micrograph Version 1.80.70.

2.2 The fabrication of electrochromic devices (ECD) and the electrochemical spectra

In the solution-based ECD, Indium tin oxide (ITO)-coated glass (~15 Ω/sq) was utilized as the electrodes and **3** and **SeV^{2+}-NHC-PtNPs** were used as active component. The two pieces of ITO glass were sealed together with a UV-cured gasket with 50 μm-thick intervals. After injection of **3** and **SeV^{2+}-NHC-PtNPs** solution into the intervals, the injection port was sealed by UV-cured gasket.[3] The electrochemical spectra were *in-situ* measured by the DH-2000-BAL Scan spectrophotometer when applying different voltages via CHI660E B157216.

2.3 Computational calculations using the Gaussian 09 code

All the computational calculations reported in this work were performed using the Gaussian 09 code. To simulate the experimental UV-vis in DMF and water solution, the Polarizable Continuum Model (PCM)[4] as a self-consistent reaction field (SCRF) was used for the calculation of equilibrium geometries, vibrational frequencies and excited state calculations. The geometries for the ground state of these compounds in the DMF solution were optimized at the B3LYP level[5] with the GenECP basis set.[6] And the keyword "opt=tight" was used.

2.4 Femtosecond transient absorption (fs-TA) measurements

Femtosecond time-resolved measurements were done by means of transient absorption, and were performed using a commercial TA system (Time-Tech Spectra, LLC). Briefly, the output from a Light Conversion soid-state diode pump regeneration amplifier [100 kHz, λ = 1030 nm, femtosecond full width at half maximum (fwhm) 290 fs] was split into a

pump and a probe part. Desired 410 nm pump wavelengths were obtained via a second harmonic generator (SHG) ORPHEUS-twins OPA (Light Conversion), and with neutral density filters the energy of each pulse was kept between ~500 nJ over ca. 3 mm^2. The white light continuum probe was obtained by focusing part of the 1030 nm light on a CaF_2 plate. Polarization of the pump was set at magic angle, 54.7°, relative to the probe. Instrumental response time depends on pump and probe wavelengths, but is typically about 300 fs. All experiments were carried out at room temperature (i.e., T = 300 K).

Data analysis are done in TAS Analyzer (Time-Tech Spectra, Co., Ltd.), and graphed on Origin 2021, a global fit to the time trajectory was performed in carpetview. Traces (ΔA vs. t) are fitted by multiple exponentials convolved with a Gaussian shaped response. All spectra are corrected for chirp in the white light probe, time zero is set at maximum pump-probe temporal overlap. Femtosecond transient absorption (fs-TA) measurements of 0.1 M acetate buffer solution using 0.05 mM **3** and **SeV^{2+}-NHC-PtNPs** were carried out respectively to analyze the photoexcitation and electron transfer processes.

2.5 EDTP-triggered H_2 production

The system containing a mixture of **SeV^{2+}-NHC-PtNPs** (0.02 μmol), EDTP (0.15mmol), and 10 mL 0.1 M acetate buffer solution (0.03 M CH_3COOH and 0.07 M CH_3COONa, pH = 5.0) were sealed in a 20 mL Pyrex bottle. After bubbling with argon for 30 min away from light, 200 μL CH_4 was injected and the bottle was exposed to the PLS-SXE300D xenon lamp with a filter (λ > 400 nm, purchasing from Beijing Perfectlight Technology Co., Ltd) at 100 mW. Then 200 μL upper gas of the reactor was injected to gas chromatography per 2 hours to measure H_2 evolution. The production of the hydrogen was calculated according to the H_2 normalized curve.

2.6 The repeatability of H_2 production

The mixture of **SeV^{2+}-NHC-PtNPs** (0.02 μmol), EDTP (0.15mmol), and 10 mL 0.1 M acetate buffer solution (0.03 M CH_3COOH and 0.07 M CH_3COONa, pH = 5.0) was bubbled with argon for 30 min away from light, and irradiated under 100 mW xenon lamp, 200 μL CH_4 was injected. Then 200 μL gas was measured by GC per 2 hours. To avoid mass-loss errors during traditional solid recovery of the fully water-soluble catalyst, an in-situ cycling protocol was employed. After 6 hours, without separating the nanoparticles, the system was directly bubbled with argon for 30 min to completely evacuate the generated H_2 and CH_4. Once restored to a hydrogen-free state, the next cycle was immediately initiated.

2.7 Photoresponsiveness of H_2 production

The mixture of **SeV^{2+}-NHC-PtNPs** (0.02 μmol), EDTP (0.15mmol), and 10 mL 0.1 M acetate buffer solution (0.03 M CH_3COOH and 0.07 M CH_3COONa, pH = 5.0) was bubbled with argon for 30 min away from light, and irradiated under 100 mW xenon lamp, 200 μL CH_4 was injected. Then 200 μL gas was measured by GC per 2 hours. After 6 h, the reaction flask was protected from light for six hours and then tested for H_2 production under dark conditions, repeating the procedure four times.

3. Synthetic procedures

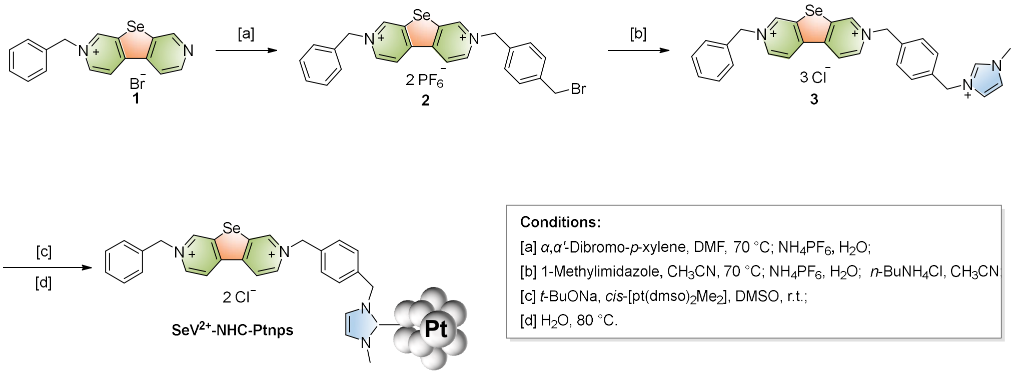

Scheme S1: Synthesis of water-soluble **SeV^{2+}-NHC-PtNPs**.

3.1 Synthesis of cis-[$PtMe_2(DMSO)_2$]

Dissolve potassium chloroplatinate (415 mg, 1.0 mmol) in water (5 mL), then add DMSO (88 mg, 1.1 mmol), stir for 2 min, stand until crystals are separated, and wash with water, EtOH and Et_2O (5 mL × 3) After drying, *cis*-$PtCl_2(DMSO)_2$ is obtained(126 mg, 30%). A solution of *cis*-$PtCl_2(DMSO)_2$ (447 mg) in DMSO (4 cm^3) was treated with $SnMe_4$ (0.3 cm^3) and the solution maintained at 70 °C for 24h. Evaporation of solvent at 70 °C gave a white solid which was washed with diethyl ether and dissolved in CH_2Cl_2.The solution was then treated with charcoal and filtered. Dry the filtrate to obtain white solid, which is the product (267 mg, 66%).

3.2 Synthesis of 2

A solution of **1** in dried and degassed DMF (15 mL) was added dropwise to a 50 mL pressure pipe containing another solution of 1,4-bis(bromomethyl)-benzene (762 mg, 2.885 mmol) in the same solvent (5 mL)under argon atmosphere. The resulting yellow solution was stirred at 70 °C for 3 days.After this period of time, the precipitate was filtered, washed with DMF (3 × 20 mL), and dried under vacuum (5 h, 70 °C, 10 mbar). The solid obtained was dispersed in H_2O (10 mL) and saturated NH_4PF_6 solution(5 mL) was dropped into the dispersion while stirring. After 8 h of reaction at room temperature, the precipitate was isolated via vacuum filtration and washed with H_2O (3 × 10 mL).The light yellow solid **2** was obtained by column chromatography after vacuum drying (1260 mg, 61%).^{1}H NMR (DMSO-d_6, 400MHz): δ 10.03-10.00 (m, 2H, Py*H*), 9.51-9.45 (m, 2H, Py*H*), 9.40 (d, *J* = 6.6Hz, 2H, Py*H*), 7.70 (s, 1H, Ph*H*), 7.61-7.57 (m, 5H, Ph*H*), 7.53-7.48 (m, 3H, Ph*H*), 6.07 (s, 4H, C*H*$_2$), 4.73(s, 2H, C*H*$_2$); ^{13}C NMR (100 MHz, DMSO): δ 146.59, 146.54, 146.45, 145.81, 144.47, 144.44, 144.40, 140.91, 140.05, 136.29, 134.28, 134.25, 133.17, 130.81,

130.66, 130.16, 129.87, 125.42, 124.84, 124.80, 64.80, 64.36, 64.15, 56.77, 34.00; HRMS (ESI^+) m/z: [**2**-2PF_6]$^{2+}$ calcd for $C_{25}H_{20}BrN_2Se$ 254.0021,found 254.0440; [**2**-PF_6]$^{1+}$ calcd for $C_{25}H_{20}BrN_2SePF_6$ 652.9690, found 652.9586; UV-vis (in DMF): λ_{max} (ε) = 416 nm (5.4 × 10^3 M^{-1} cm^{-1}).

3.3 Synthesis of 3

A solution of **2** (1260 mg, 1.578 mmol) in dried and degassed acetonitrile (10 mL) was added dropwise to a 25 mL pressure-resistant tube containing another solution of *N*-methylimidazole (189 µl, 2.368 mmol) in the same solvent (2 mL) under argon atmosphere. The resulting yellow solution was stirred at 70 °C for 3 days. After this period of time, the precipitate was filtered, washed with acetonitrile (3 × 20 mL), and dried under vacuum (5 h, 70 °C, 10 mbar). The solid obtained was dispersed in H_2O (10 mL) and saturated NH_4PF_6 solution (5 mL) was dropped into the dispersion while stirring. After reacted for 12 h at room temperature, the precipitate was isolated via vacuum filtration, washed with H_2O (3 × 10 mL), and dried under vacuum (24 h, 70 °C, 10 mbar). The resulting light yellow solid was then dispersed in acetonitrile (10 ml) and a saturated acetonitrile solution of tetrabutylammonium chloride (5 ml) was added dropwise to the dispersion under stirring. After 6 h of reaction at room temperature, the precipitate was isolated by vacuum filtration, washed with acetonitrile (3 × 10 ml) and dried under vacuum to obtain **3** (260 mg, 18%). ^{1}H NMR (DMSO-d_6, 400 MHz): δ 10.01 (s, 2H, Py*H*), 9.49 (d, *J* = 6.3 Hz, 1H, Py*H*), 9.44 (d, *J* = 6.4Hz, 1H, Py*H*), 9.39 (d, *J* = 6.2Hz, 2H, Py*H*), 9.19 (s, 1H, Imz*H*), 7.74 (s, 1H, Imz*H*), 7.70 (s, 1H, Imz*H*), 7.64-7.59 (m, 4H, Ph*H*), 7.53-7.50 (m, 5H, Ph*H*), 6.07 (s, 4H, CH_2), 5.44 (s, 2H, CH_2), 3.84 (s, 3H, CH_3); ^{13}C NMR (100 MHz, DMSO): δ 146.55, 146.47, 146.38, 146.26, 146.21, 145.20, 145.06, 140.62, 140.56, 137.33, 136.72, 134.82, 134.51,

130.54, 130.05, 129.84, 129.78, 124.91, 124.84, 124.45, 122.85, 64.59, 64.01, 51.72, 36.33; HRMS (ESI^+) m/z: [**3**-3Cl]$^{3+}$ calcd for : $C_{29}H_{27}N_4Se$ 170.3795,found 170.3716; [**3**-2Cl]$^{2+}$ calcd for $C_{29}H_{27}N_4SeCl$ 273.0539, found 273.0535; [**3**-Cl]$^{+}$ calcd for $C_{29}H_{27}N_4SeCl_2$ 581.0773, found 581.0745; UV-vis (in DMF): λ_{max} (ε) = 417 nm (4×10^3 M^{-1} cm^{-1}).

3.4 Synthesis of SeV^{2+}-NHC-PtNPs

To a solution of *cis*-[$PtMe_2(DMSO)_2$] (161 mg, 0.42 mmol) and **3** (260 mg, 0.42 mmol) in DMSO (10 mL) was added sodium tert-butoxide (40.36 mg, 0.42 mmol). The mixture was stirred at room temperature for 1 h and filtered through a diatomaceous earth plug. After complete evaporation of the solvent (70 °C, 4 mbar), the resulting reddish brown solid was introduced into a 10 ml pressure-resistant tube and dissolved in deionized water (5 ml) The resulting reddish brown liquid was stirred at 1000 rpm and heated at 80 °C for 18 h. After this time, the black solution was allowed to slowly reach room temperature and then filtered through a PTFE 0.2 μm filter to remove any particles from the suspension. The obtained solution was dialyzed using a cellulose membrane (MWCT = 14000 Dalton) for 36 h. The solvent was removed under vacuum (3 h, 50 °C, 100 mbar) to obtain a black solid, which was dried under vacuum (25 °C, 10 mbar) overnight. The black powder obtained was platinum nanoparticles **SeV^{2+}-NHC-PtNPs** (62 mg, 18%). UV-vis (in H_2O): λ_{max} (ε) = 400 nm (4.3×10^3 M^{-1} cm^{-1}).

4. Theoretical Calculations

4.1 Calculation of the total number of platinum atoms in the SeV^{2+}-NHC-PtNP

Assuming the **SeV^{2+}-NHC-PtNP** is ideal spheres, its volume(v_{np}):

$$v_{np} = \frac{4}{3}\pi R^3 \tag{1}$$

According to TEM analysis, the average radius of **SeV^{2+}-NHC-PtNPs** $R = 0.9$nm.

The molar atomic volume of platinum(v_m):

$$v_m = \frac{M}{\rho} \tag{2}$$

Where the molar mass of platinum atoms M = 195.084 g/mol ,the solid-state density of platinum atoms ρ = 21.45 g/cm^3.

The atomic volume of platinum(v_a):

$$v_a = \frac{v_m}{N_A} \tag{3}$$

where Avogadro constant $N_A = 6.02 \times 10^{23}$mol^{-1}.

The total number of platinum atoms contained in the **SeV^{2+}-NHC-PtNP**(N_T):

$$N_T = \frac{v_{np}}{v_a} \tag{4}$$

According to Equations (1) to (4), the calculation yields $N_T = 202$.

4.2 Calculation of the coverage of organic ligands on the surface of SeV^{2+}-NHC-PtNPs

According to HRTEM analysis, **SeV^{2+}-NHC-PtNPs** exhibit highly crystalline characteristics with a fcc cubic structure, and reflections corresponding to the (111) crystal plane are present in all cases. Therefore, assuming **SeV^{2+}-NHC-PtNPs** are ideal spheres, their surface atomic arrangement follows the (111) crystal plane.

The surface area of the **SeV^{2+}-NHC-PtNP**(S_{np}):

$$S_{np} = 4\pi R^2 \tag{5}$$

where the average radius of **SeV^{2+}-NHC-PtNPs** $R = 0.9$nm.

The area of the (111) crystal plane within the unit cell($S_{(111)}$):

$$S_{(111)} = \frac{\sqrt{3}}{4}\left(\sqrt{2}a\right)^2 \quad (6)$$

where the lattice constant $a = 0.3923$nm.

The effective number of atoms on the (111) crystal plane within the unit cell($N_{(111)}$):

$$N_{(111)} = 3 \times \frac{1}{6} + 3 \times \frac{1}{2} = 2 \quad (7)$$

The area occupied by each surface atom on the (111) crystal plane(S_{atom}):

$$S_{atom} = \frac{S_{(111)}}{N_{(111)}} \#\# \quad (8)$$

The number of surface atoms in the **SeV^{2+}-NHC-PtNP**(N_S):

$$N_S = \frac{S_{np}}{S_{atom}} \quad (7)$$

According to Equations (5) to (9) ,the calculation yields $N_S = 152$.

Since the particle size of **SeV^{2+}-NHC-PtNPs** is less than 2 nm, it is considered that the XPS detection depth encompasses the entire SeV^{2+}-NHC-PtNP from surface to core. Therefore, based on the ratio of the high-valent platinum peak area to the total peak area in the XPS Pt4f spectrum, the proportion of high-valent platinum atoms among all platinum atoms in the nanoparticles can be estimated. At the same time, based on previous analysis, high-valent platinum atoms are precisely those atoms on the SeV^{2+}-NHC-PtNP surface that have bonded with organic ligands.

The proportion of surface atoms bonded to organic ligands among all atoms in the **SeV^{2+}-NHC-PtNP**($\varepsilon_{s\text{-}NHC}$):

$$\varepsilon_{s\text{-}NHC} = \frac{S_{pt^{\delta+}4f_{7/2}} + S_{pt^{\delta+}4f_{5/2}}}{S_{pt^{\delta+}4f_{7/2}} + S_{pt^{\delta+}4f_{5/2}} + S_{pt^{0}4f_{7/2}} + S_{pt^{0}4f_{5/2}}} \quad (8)$$

where $S_{pt^0 4f_{5/2}}$ = 4416.68(74.2ev), $S_{pt^0 4f_{7/2}}$ = 7043.34(71.0ev), $S_{pt^{\delta+} 4f_{5/2}}$ = 3635.53(75.7ev), $S_{pt^{\delta+} 4f_{7/2}}$ = 3515.42(72.5ev).

The number of surface atoms bonded to organic ligands in the **SeV²⁺-NHC-PtNP**($N_{S\text{-}NHC}$):

$$N_{S\text{-}NHC} = N_T \cdot \varepsilon_{s\text{-}NHC} \tag{9}$$

Among all surface atoms in the **SeV²⁺-NHC-PtNP**, the proportion of surface atoms forming bonds with organic ligands(*cov%*):

$$cov\% = \frac{N_{S\text{-}NHC}}{N_S} \times 100\% \tag{10}$$

According to Equations, we obtain *cov*% = 51%.

The proportion of surface atoms relative to all atoms in the SeV^{2+}-NHC-PtNP (ε_s):

$$\varepsilon_s = \frac{N_S}{N_T} \times 100\% \tag{11}$$

According to Equations (5) to (13), the calculation yields ε_s = 75%.

4.3 Calculation of the Metal : Ligand (M:Lig) Ratio on the Surface of the SeV²⁺-NHC-PtNP

After conducting TGA testing on ligand molecule **3** (Figure S2), it was observed that ligand molecule **3** decomposes almost completely when heated above 600°C in air (residual mass fraction = 0.49%, negligible). Therefore, it can be concluded that the residual portion of the **SeV²⁺-NHC-PtNP** after heating under identical conditions consists entirely of the non-decomposable metallic component (platinum). Following multiple TGA tests on the **SeV²⁺-NHC-PtNP** (Figure S2), the following curve was plotted based on the average values. Within the 200°C range, mass loss primarily originates from intermolecularly bound water. This indicates that the **SeV²⁺-NHC-PtNP** sample contains 7.73%(ε_1) intermolecularly bound water, 27.52%(ε_2) platinum, and 64.75%(ε_3) organic components.

The number of the PtNP core contained in the sample $\left(N_{np}\right)$:

$$N_{np}=\frac{M_{pt}}{m_{np}}=\frac{M_{Sample}\cdot\varepsilon_2}{\rho\cdot v_{np}} \tag{12}$$

where sample mass M_{Sample} = 1.8210 mg, M_{pt} represents the platinum content in the sample, m_{np} represents the mass of a PtNP core.

The number of organic ligands contained in the sample (N_{NHC}):

$$N_{NHC} = N_A \cdot n_{NHC} = N_A \cdot \frac{M_{org}}{M_{NHC}} = N_A \cdot \frac{M_{Sample}\cdot\varepsilon_3}{M_{NHC}} \tag{13}$$

where the relative molecular mass of the organic ligand M_{NHC} = 582.5, n_{NHC} represents the molar quantity of organic ligands contained in the sample, M_{org} represents the organic content in the sample.

Assuming an equal number of ligands per PtNP core, the number of ligands per PtNP core $N_{\frac{NHC}{np}}$ is:

$$N_{\frac{NHC}{np}} = \frac{N_{NHC}}{N_{np}} \tag{14}$$

According to Equations(1)(14)(15)(16), the (M : Lig) Ratio =N_S ： $N_{\frac{NHC}{np}}$ = 1:1.03

4.4 Calculation of the relative molecular mass of the SeV^{2+}-NHC-PtNP

Based on the preceding calculation results, the composition of the **SeV^{2+}-NHC-PtNP** includes 202 platinum atoms and 78 organic ligands.

Therefore, the relative molecular mass of the **SeV^{2+}-NHC-PtNP** $\left(M_{np}\right)$:

$$M_{np} = M\cdot N_T + M_{NHC} \cdot N_{S\text{-}NHC} \tag{15}$$

According to Equations(4)(11), the calculation yields M_{np}=84259.47.

5. ATR-FTIR spectra of the imidazolium salts and Pt nanoparticles

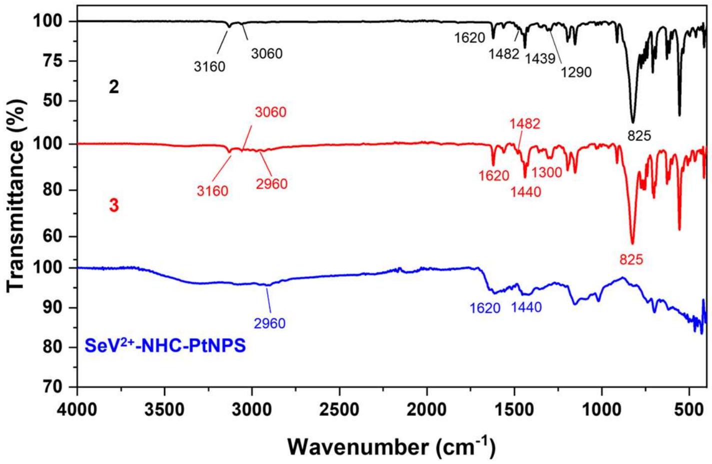

Figure S1: ATR-FTIR spectra of **2**, **3** and **SeV^{2+}-NHC-PtNPs**.

Table S1: Detailed ATR-FTIR peak assignments for **2**, **3**, and **SeV^{2+}-NHC-PtNPs**

Compound	Wavenumber (cm^{-1})	Vibrational Mode & Functional Group Assignment
**2**	3130	N-H stretching vibration (pyridine ring)
	3060	C-H stretching vibration (pyridine ring)
	1620	C=N stretching vibration (pyridine ring)
	1482, 1439	C-H deformation vibration (pyridine skeleton)
	1290	C-N stretching vibration (pyridine skeleton)
	825	Aromatic C-H out-of-plane bending (benzyl ring)
**3**	3130	N-H stretching vibration (pyridine ring)
	3060	C-H stretching vibration (pyridine ring)
	2960	C-H stretching vibration (methyl group)
	1620	Overlapping C=N stretching vibrations (pyridine ring & imidazole ring)
	1482, 1440	C-H deformation vibration (pyridine skeleton)
	1440	Deformation vibration (imidazole ring)
	1300	Overlapping C-N stretching vibrations (pyridine skeleton & imidazole ring)
	825	Aromatic C-H out-of-plane bending (benzyl ring)
**SeV^{2+}-NHC-PtNPs**	2960, 1620, 1440	Main characteristic peaks of the organic ligands are retained but exhibit noticeable peak broadening

6. TGA analysis

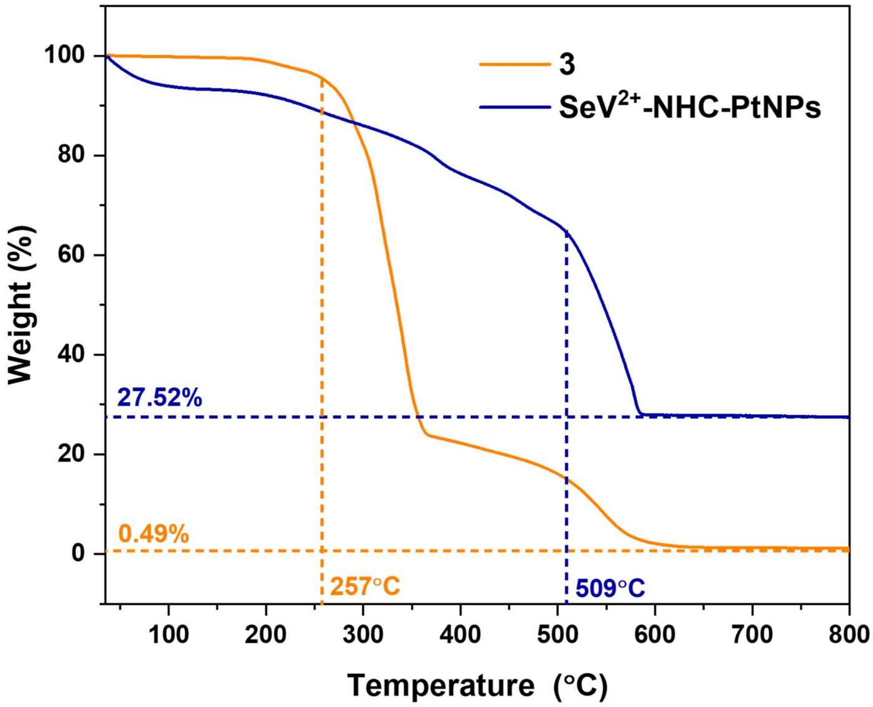

Figure S2: TGA of **3** and **SeV^{2+}-NHC-PtNPs** under air conditions.

7. The XPS spectra

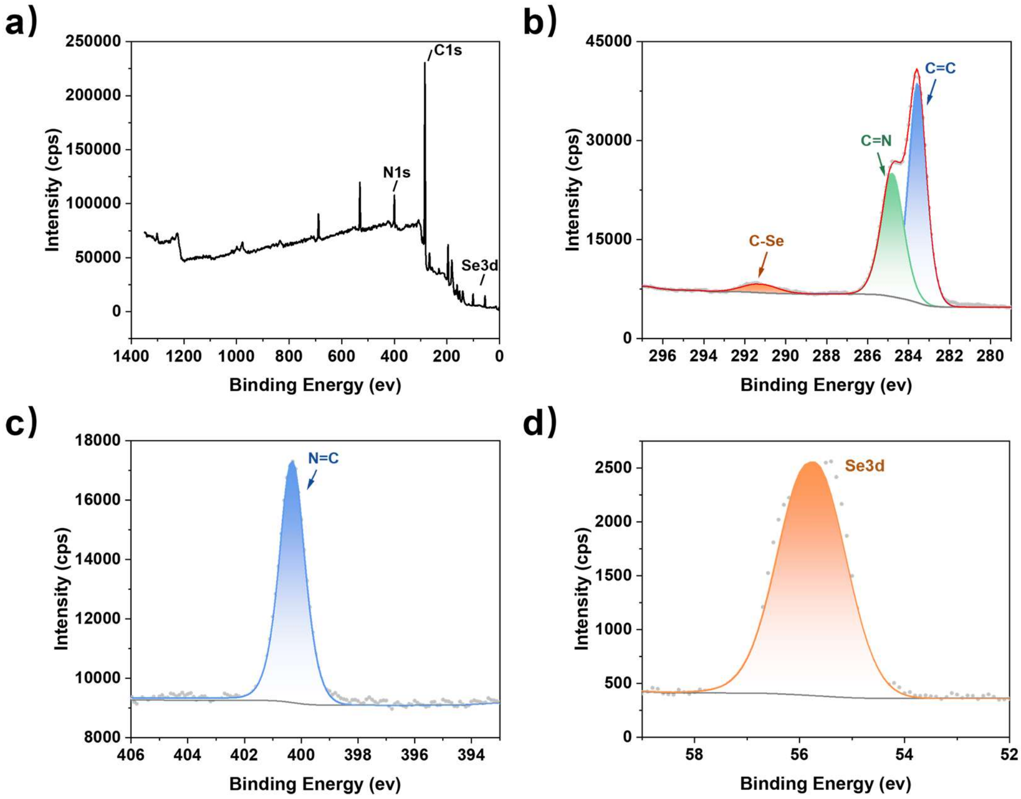

Figure S3: The XPS spectra of **3**. a, Full XPS spectrum of **3**. b, High-resolution XPS spectra of C 1s. c, High-resolution XPS spectra of N 1s. d, and (d) High-resolution XPS spectra of Se 3d.

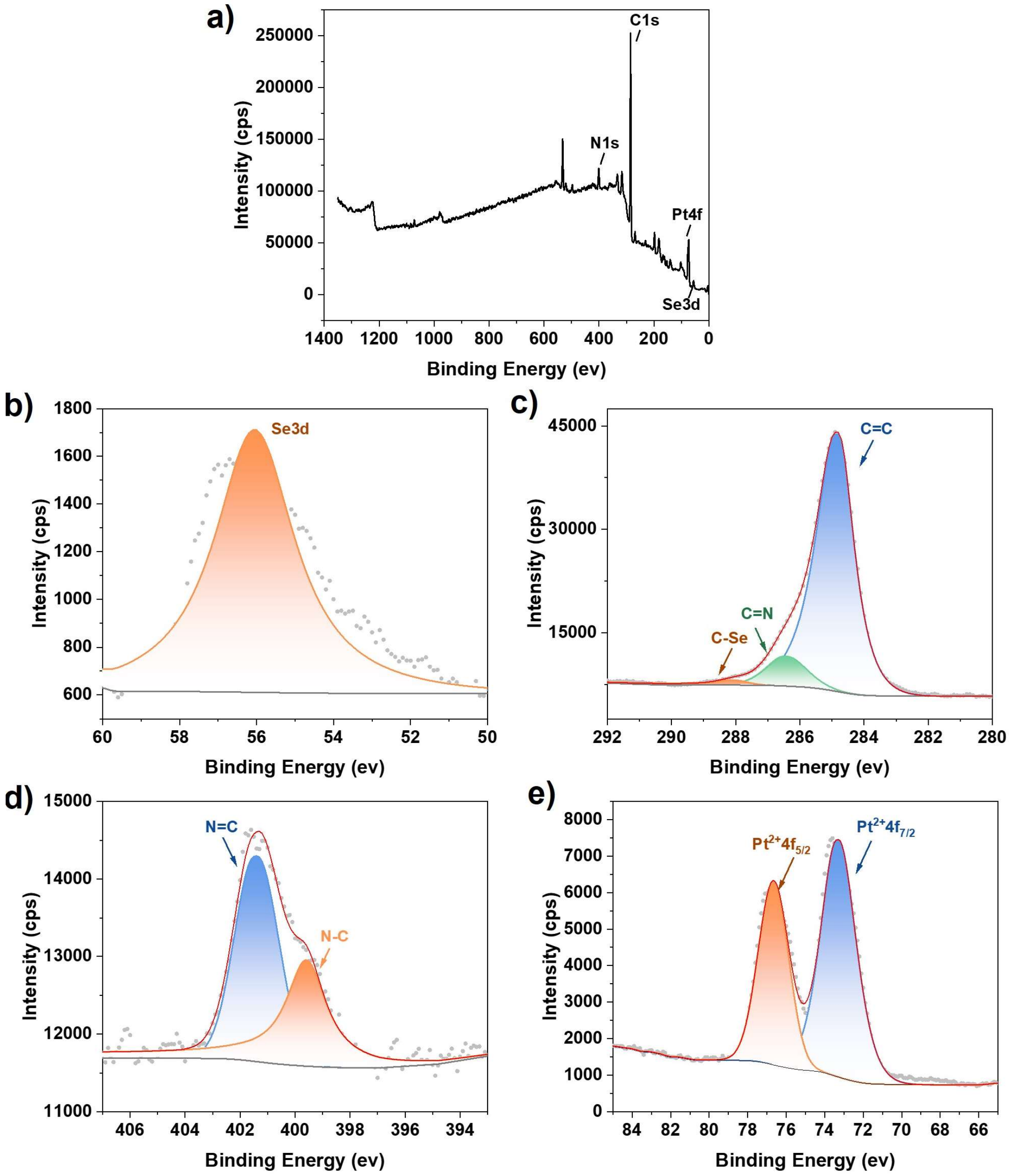

Figure S4: The XPS spectra of **[SeV^{2+}-NHC-Pt]**. a, Full XPS spectrum of **[SeV^{2+}-NHC-Pt]**. b, High-resolution XPS spectra of Se 3d. c, High-resolution XPS spectra of C 1s. d, High-resolution XPS spectra of N 1s. e, High-resolution XPS spectra of Pt 4f.

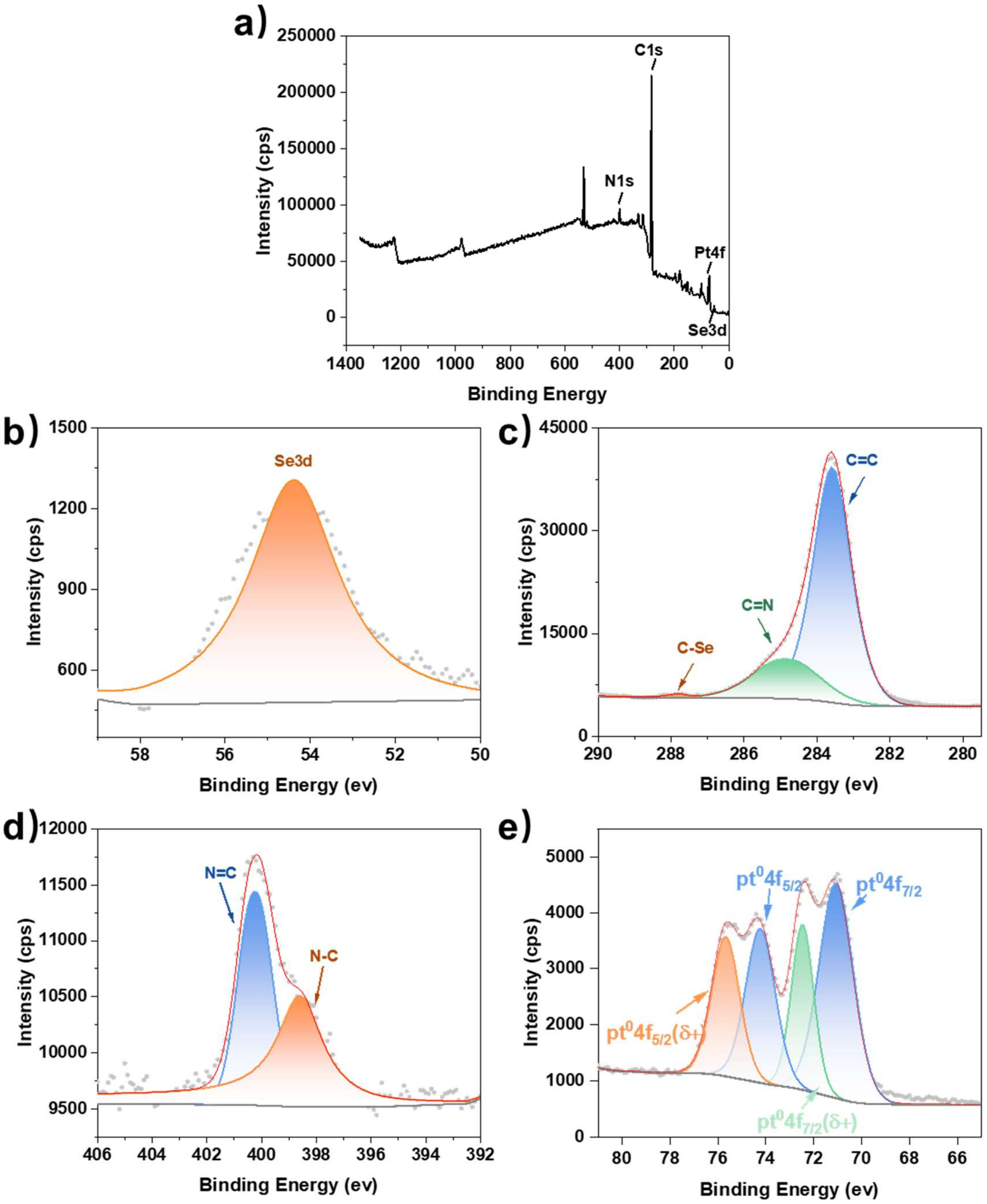

Figure S5: The XPS spectra of **SeV^{2+}-NHC-PtNPs**. a, Full XPS spectrum of **SeV^{2+}-NHC-PtNPs**. b, High-resolution XPS spectra of Se 3d. c, High-resolution XPS spectra of C 1s. d, High-resolution XPS spectra of N 1s. e, High-resolution XPS spectra of Pt 4f.

8. Optical characterization data

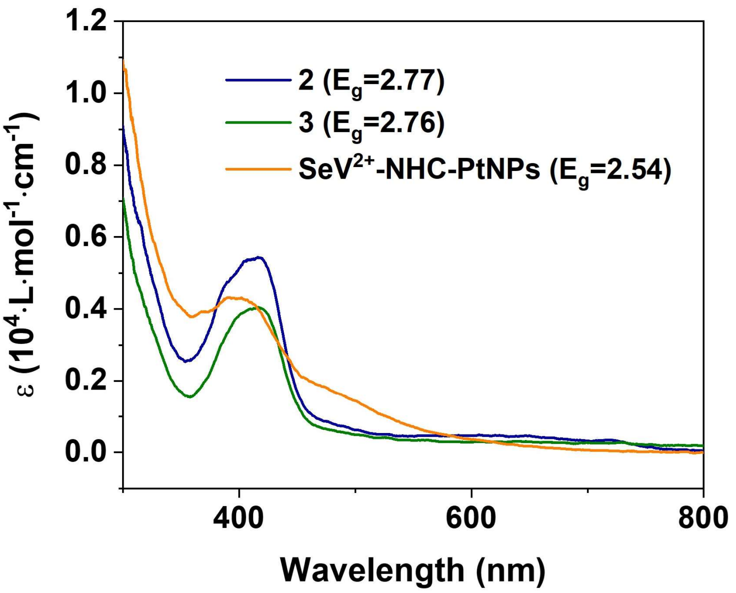

Figure S6: UV-vis spectra of **2** and **3** in DMF and **SeV^{2+}-NHC-PtNPs** in water, c~10^{-4} M; extrapolated optical band gaps (Eg) are shown as insets.

Table S2: Optical and Electronic Properties of Chalcogen-Bridged Viologens.

Compd	λ_{max} [nm] (ε[M^{-1}•cm^{-1}])	E_{red} [V]	E_g [eV][a] (calcd)[b]	E_{LUMO} [eV][c] (calcd)	E_{HOMO} [eV] (calcd)
**2**	416 (5400)	-0.42, -0.50, -0.87, -0.95	2.77	-4.38	-7.15
**3**	417 (4000)	-0.42, -0.49, -0.86, -0.97	2.76 (3.54)	-4.38 (-3.59)	-7.14 (-7.04)
**SeV^{2+}-NHC-PtNPs**	400 (4300)	-	2.64 (1.98)	(-3.31)	(-5.29)

[a] Energy gap values were calculated from the absorption spectra. [b] Theoretical calculations have been carried out by using the GAUSSIAN09 suite of programs. [c] Energy levels vs vacuum level were calculated from CV data and from the optically determined energy gap.

9. The cyclic voltammogram

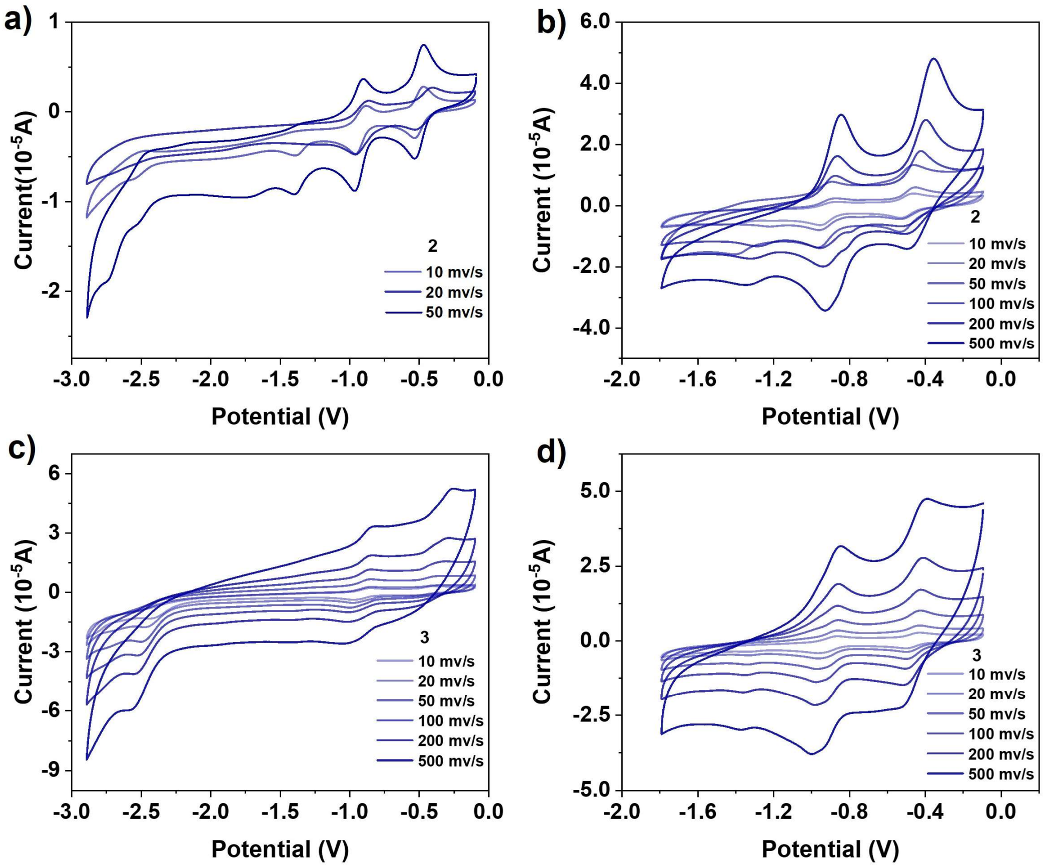

Figure S7: The cyclic voltammogram of (a) (b) **2** and (c) (d) **3** at different scan rates in DMF solution with tetrabutylammonium hexafluorophosphate (0.05 M) as supporting electrolyte, potential E referenced to Fc/Fc^{+}, c~10^{-3} M.

10.Evaluation of electron-transfer constant k_{ET}

The electron-transfer constants k_{ET} were determinated using the Nicholson method according to our previous work.[7]

$$i_p=2.69\times10^5 A D_0^{1/2} v^{1/2} c^*=Rv^{1/2} \quad (18)$$

where electrode radius r = 0.15 cm, electrode area $A = \pi r^2$ = 0.07065 cm^2, concentration $c^*=10^{-3}$ mol/cm^3.

When scan rate v = 0.1 V/s,

$$k_{ET}=\Psi(\pi D_0 Fv/RT)^{1/2}=184\Psi R \quad (19)$$

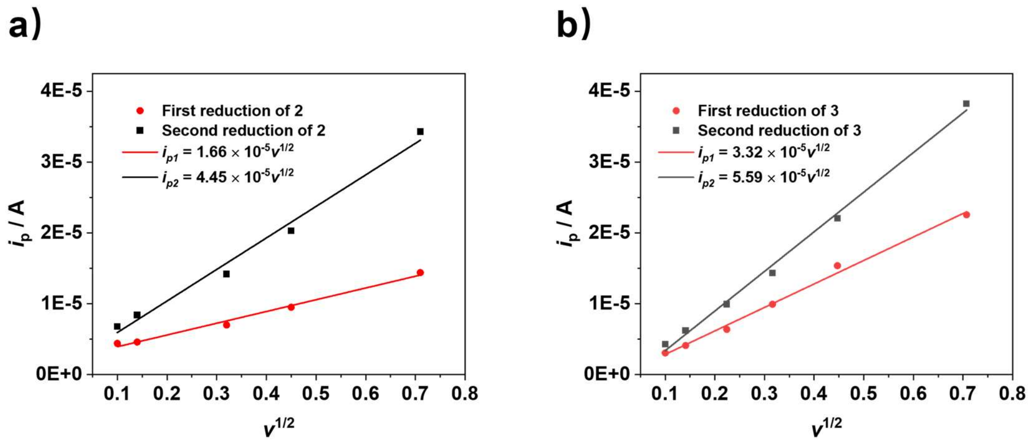

Figure S8: Peak current and scan rate diagrams of (a) **2** and (b) **3**.

Table S3: Electron-transfer constant k_{ET} of **2** and **3**.

Compd	R_1[a]	ΔE_{p1} [mV][b]	Ψ_1[c]	R_2[a]	ΔE_{p2} [mV][b]	Ψ_2[c]	k_{ET1}[d]	k_{ET2}[e]
2	4.45 $\times10^{-5}$	83	1.106	1.66 $\times10^{-5}$	76	1.6	9.05 $\times10^{-3}$	4.89 $\times10^{-3}$
3	3.32 $\times10^{-5}$	114	0.415	5.59 $\times10^{-5}$	84	1.057	2.53 $\times10^{-3}$	1.09 $\times10^{-2}$
BnSeV^{2+}	1.00 $\times10^{-5}$	100	0.630	-	-	-	1.16 $\times10^{-3}$	-

[a] Slope of ip~$v^{1/2}$ in Supplementary Fig. 7. [b] ΔEp was calculated from CV. [c] Ψ = (-0.6288+0.0021ΔEp)/(1-0.017ΔEp). [d] Electron-transfer constant k_{ET} was evaluated according to Nicholson's formula.

11. Computed UV/vis spectra

The simulated UV-vis spectra for optimized molecules were performed at the time dependent density functional theory (TD-DFT) at the ground-state equilibrium geometries, both low-lying singlet and triplet states were determined using the B3LYP in association with the GenECP basis set.

The calculated maximum absorption wavelength (λTD-DFT), oscillator strength (f) and molecular orbitals (MOs) involved in the main transitions of **3** in DMF and of **SeV^{2+}-NHC-PtNPs** in water are reported in Supplementary Tables 3-4,respectively. To make it well suitable for efficiently and reasonably accurate determination of the excited state properties, the structures and frequencies of compounds **3** in the DMF solution as well as compound **SeV^{2+}-NHC-PtNPs** in water were optimized at the B3LYP level with the GenECP basis set, the keyword “opt=tight” was used.

It should be pointed out that the structures of all stationary points in DMF solvent as well as in aqueous solution were fully optimized, and frequency calculations were performed at the same level. The frequency calculations confirmed the nature of all revealed equilibrium geometries: there were no imaginary frequencies.

Table S4: Calculated (λTD-DFT) wavelengths (nm) of **3**. Molecular orbitals (MOs) involved in the main electronic transition, f corresponds to the oscillator strength.

$\lambda_{TD\text{-}DFT}$	Oscillator Strength, f	MOs			
**411.15**	0.0784	130 → 131	HOMO → LUMO	0.69995	97.99%
**375.17**	0.0315	129 → 131	HOMO-1 → LUMO	0.70129	98.36%
**364.64**	0.0360	127 → 131	HOMO-3 → LUMO	0.70284	98.80%
**253.06**	1.0970	123 → 131	HOMO-7 → LUMO	0.27008	14.58%
		124 → 131	HOMO-6 → LUMO	-0.32319	20.89%
		130 → 132	HOMO →LUMO+1	0.35460	25.15%
		130 → 133	HOMO → LUMO+2	-0.33618	22.60%
		130 → 134	HOMO → LUMO+3	0.24826	12.33%

Table S5: Calculated (λTD-DFT) wavelengths (nm) of **SeV^{2+}-NHC-PtNPs**. Molecular orbitals (MOs) involved in the main electronic transition, f corresponds to the oscillator strength.

$\lambda_{TD\text{-}DFT}$	Oscillator Strength, f	MOs			
**421.14**	0.0641	134 → 140	HOMO-5 → LUMO	0.69961	97.90%
**396.59**	0.0857	137 → 144	HOMO-2 → LUMO+4	0.43583	37.99%
		137 → 145	HOMO-2 → LUMO+5	0.11106	2.47%
		139 → 144	HOMO → LUMO+4	0.49645	49.29%
		139 → 145	HOMO → LUMO+5	0.13113	3.44%
**387.73**	0.0371	133 → 140	HOMO-6 → LUMO	0.61044	74.53%
		136 → 144	HOMO-3 → LUMO+4	0.32079	20.58%

**283.03**	0.1205	127 → 140	HOMO-12 → LUMO	0.48666	47.37%
		134 → 142	HOMO-5 → LUMO+2	0.30136	18.16%
		134 → 143	HOMO-5 → LUMO+3	0.15881	5.04%
		136 → 145	HOMO-3 → LUMO+5	-0.13891	3.86%
		136 → 146	HOMO-3 → LUMO+6	-0.16028	5.14%
		136 → 148	HOMO-3 → LUMO+8	0.13210	3.49%
		137 → 148	HOMO-2 → LUMO+8	0.17643	6.23%
		137 → 150	HOMO-2 → LUMO+10	-0.14219	4.04%

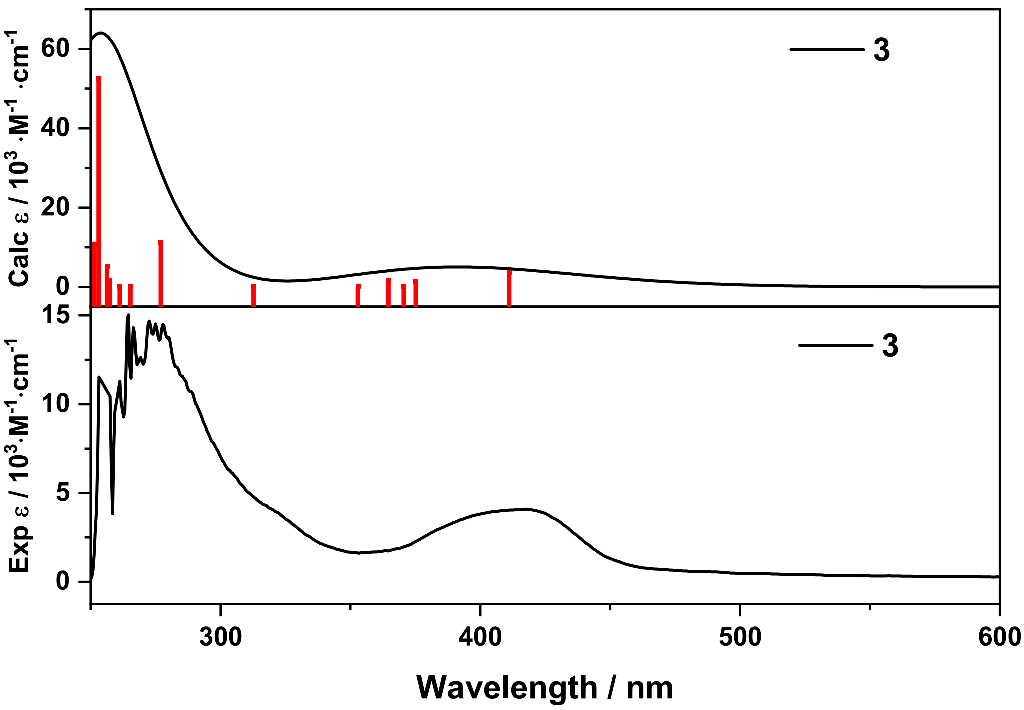

Figure S9: Computed, at the RB3LYP TD-FC/GenECP level of theory in the DMF, and experimental UV-vis spectra of **3**.

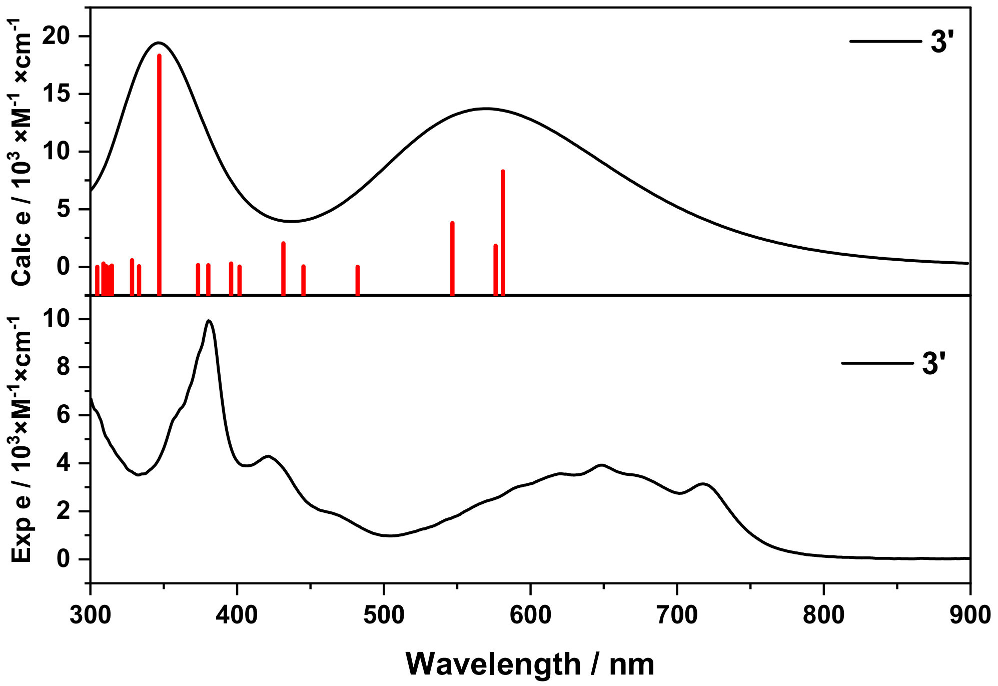

Figure S10: Computed, at the RB3LYP TD-FC/GenECP level of theory in the DMF, and experimental UV-vis spectra of **3'**.

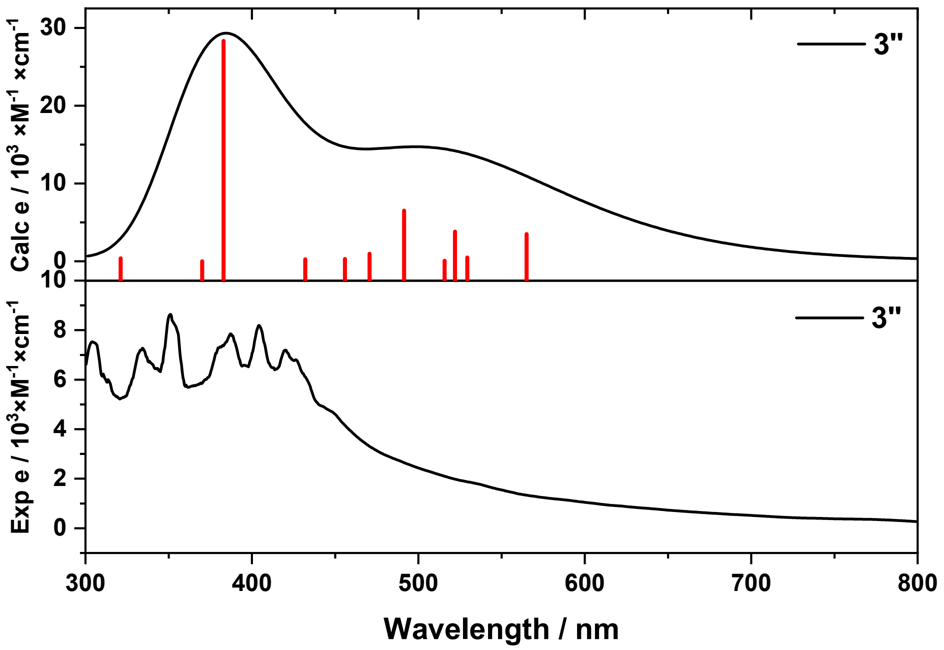

Figure S11: Computed, at the RB3LYP TD-FC/GenECP level of theory in the DMF, and experimental UV-vis spectra of **3''**.

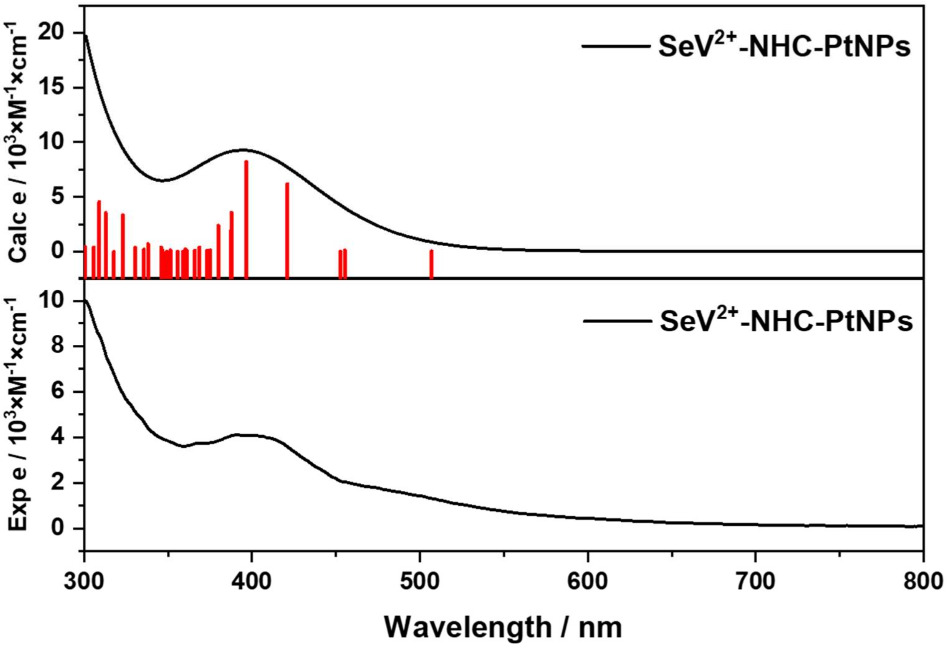

Figure S12: Computed, at the RB3LYP TD-FC/GenECP level of theory in the DMF, and experimental UV-vis spectra of **SeV^{2+}-NHC-PtNPs**.

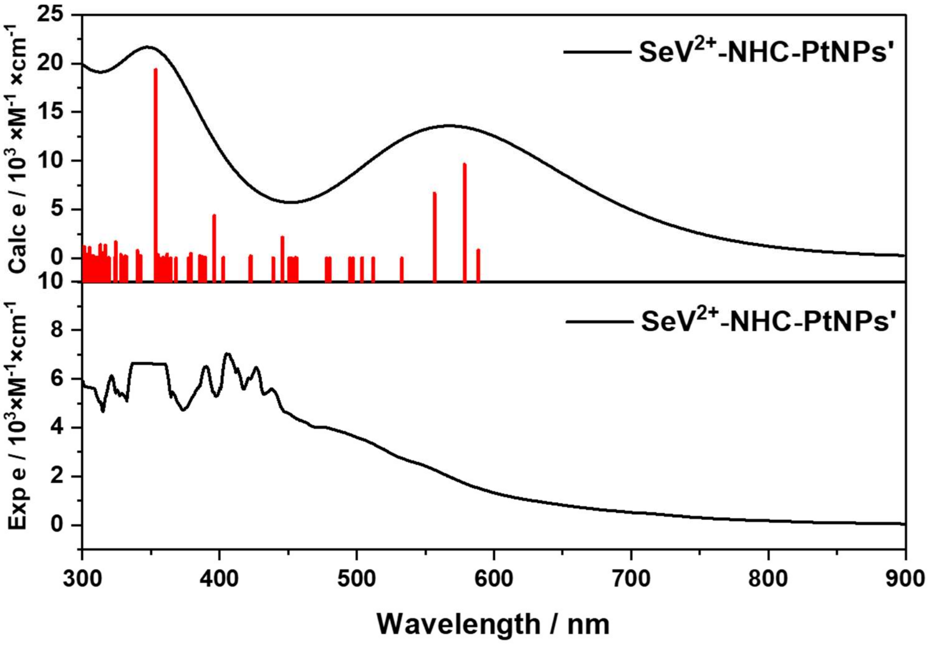

Figure S13: Computed, at the RB3LYP TD-FC/GenECP level of theory in the DMF, and experimental UV-vis spectra of **SeV^{2+}-NHC-PtNPs'**.

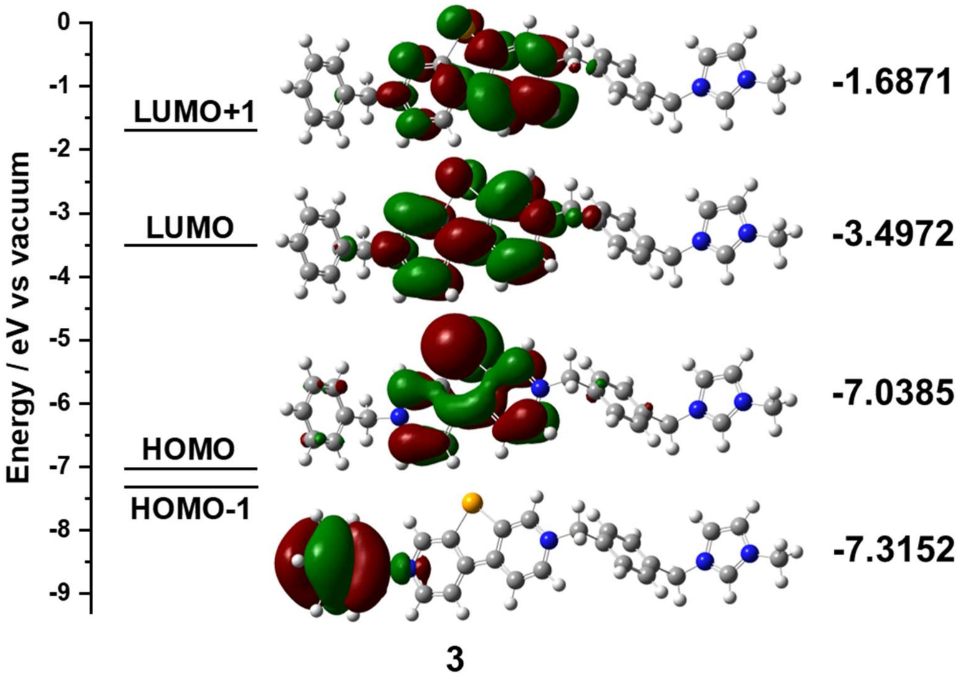

Figure S14: Computed frontier orbitals of **3** in DMF.

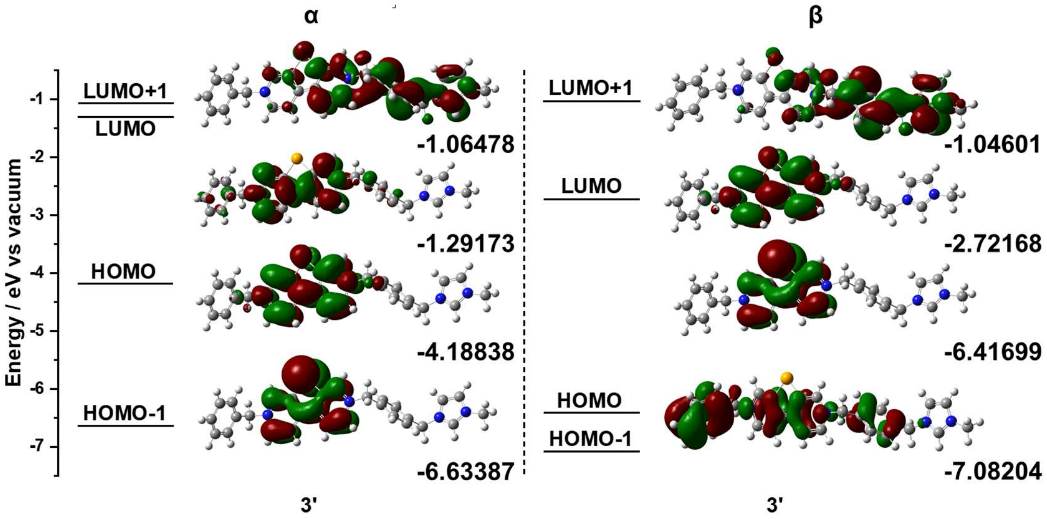

Figure S15: Computed frontier orbitals of **3’** in DMF.

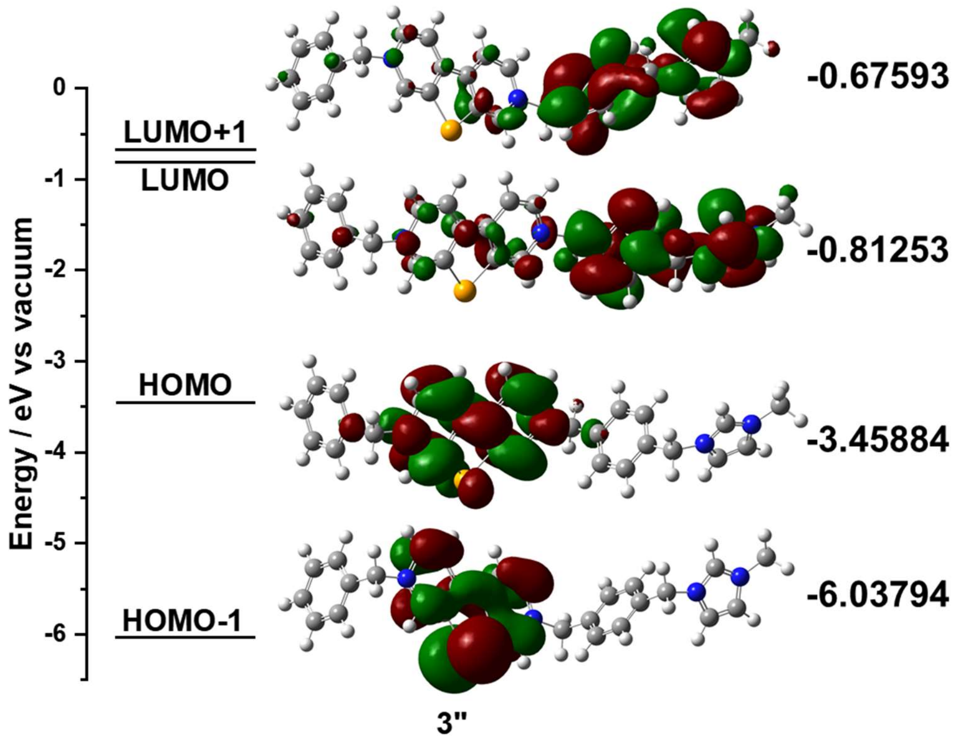

Figure S16: Computed frontier orbitals of **3''** in DMF.

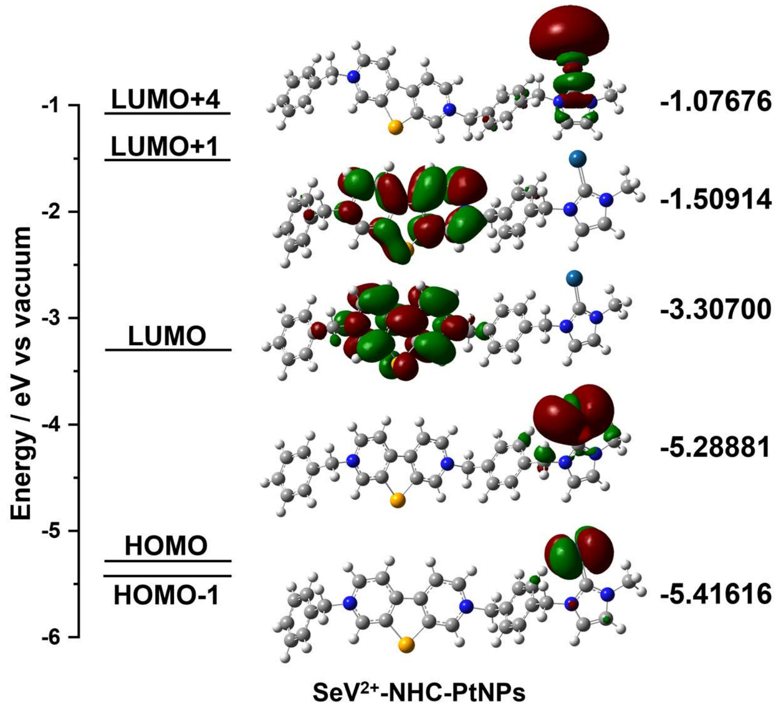

Figure S17: Computed frontier orbitals of **SeV^{2+}-NHC-PtNPs** in H_2O.

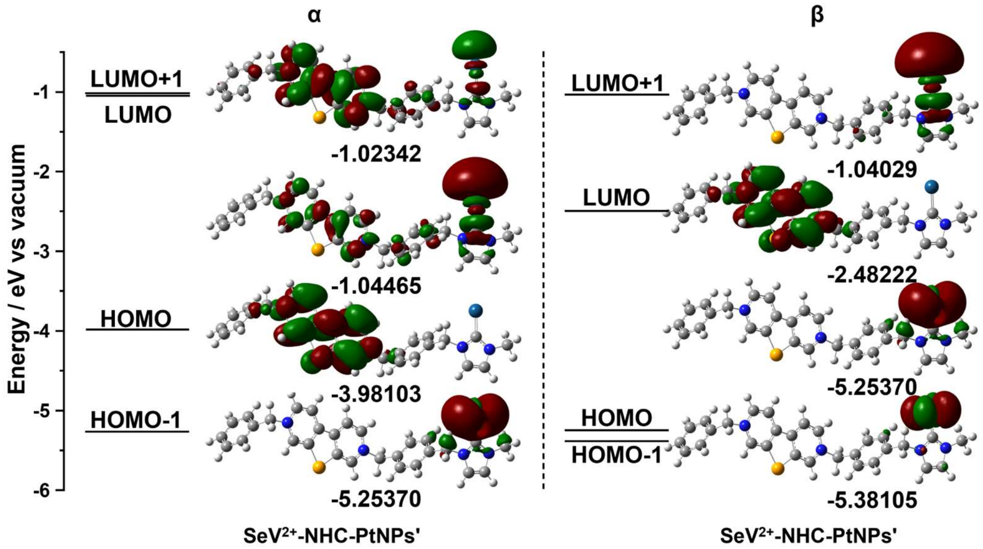

Figure S18: Computed frontier orbitals of **SeV^{2+}-NHC-PtNPs’** in H_2O.

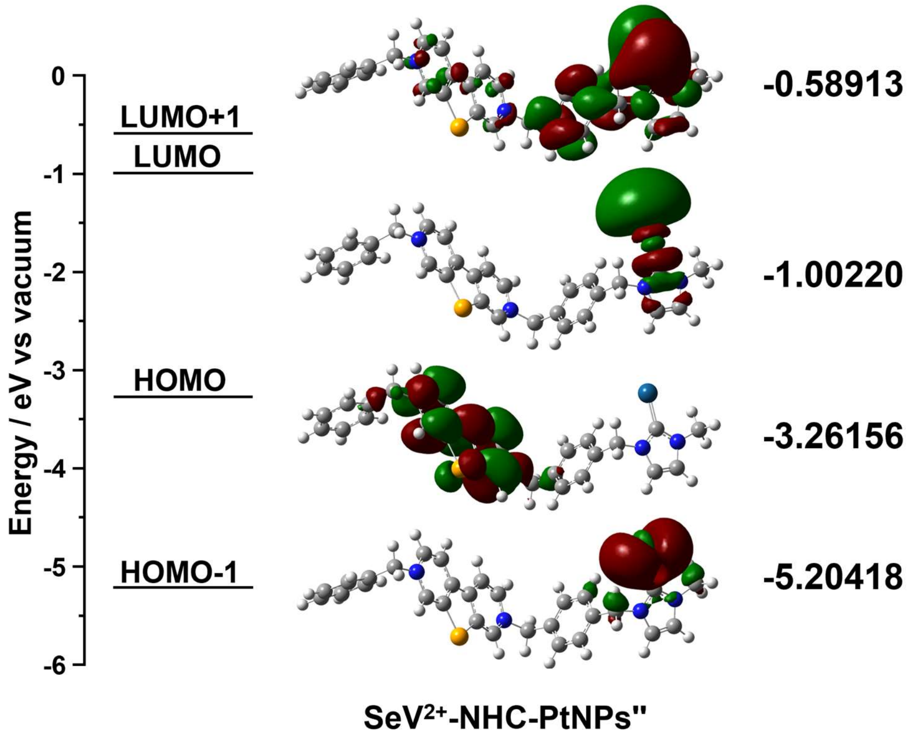

Figure S19: Computed frontier orbitals of **SeV^{2+}-NHC-PtNPs’’** in H_2O.

12.Calculated spin density plots for the radical species

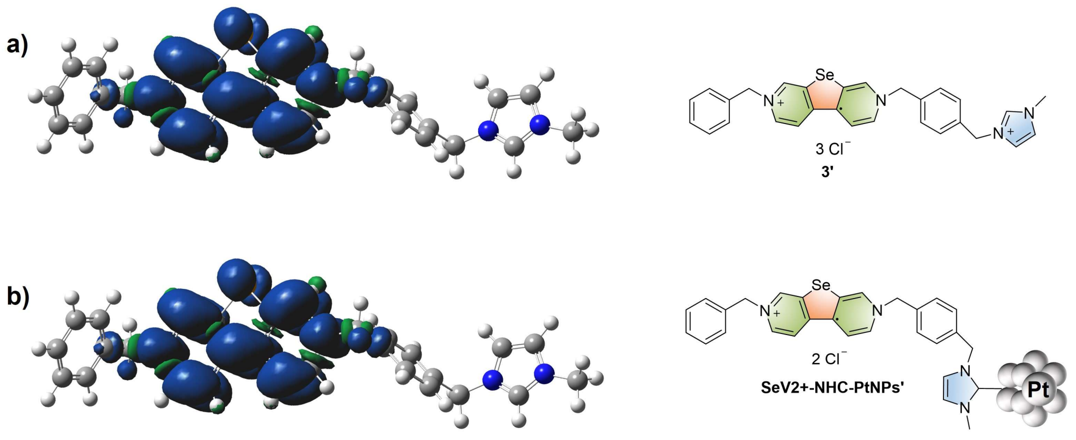

Figure S20: The calculated spin density plots of radical species (a) **3’** and (b) **SeV^{2+}-NHC-PtNPs’** in the triplet ground state.

13. Electrostatic potential surfaces

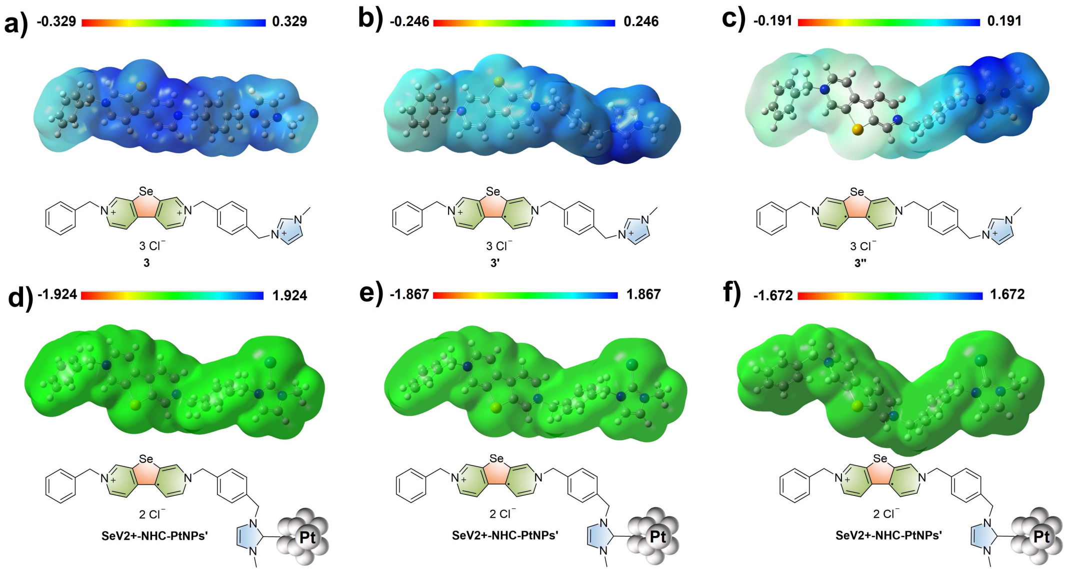

Figure S21: Electrostatic potential surfaces of (a) **3**, (b) **3**’, (c) **3**’’, (d) **SeV^{2+}-NHC-PtNPs**, (e) **SeV^{2+}-NHC-PtNPs’** and (f) **SeV^{2+}-NHC-PtNPs’’**.

14. Electrochromism of SeV2+-NHC-PtNPs

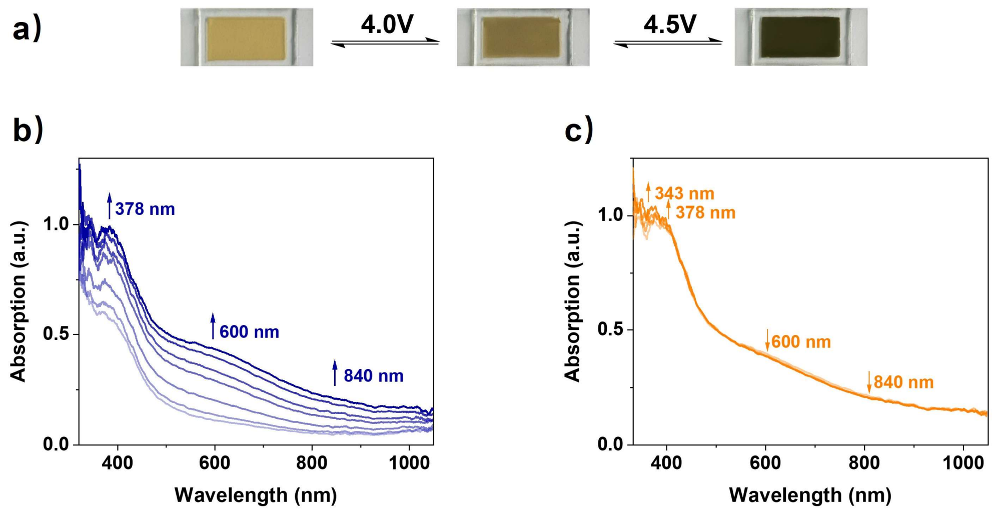

Figure S22: Electrochromism of **SeV^{2+}-NHC-PtNPs**. a, Solution-based electrochromic device with **SeV^{2+}-NHC-PtNPs** (no electrolyte). b, Spectroelectrochemistry of **SeV^{2+}-NHC-PtNPs** for first reduction; c, Spectroelectrochemistry of **SeV^{2+}-NHC-PtNPs** for second reduction.

15. UV-vis spectra of two redox states of SeV2+-NHC-PtNPs

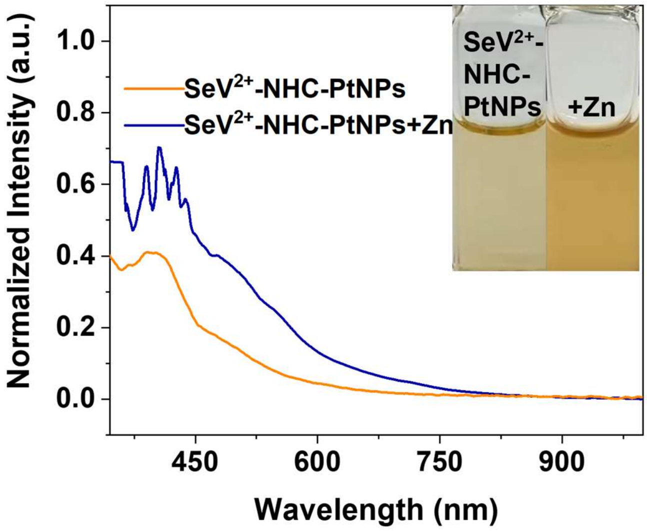

Figure S23: UV-vis spectra of two redox states of **SeV^{2+}-NHC-PtNPs** (c = 10^{-4} M). Inset picture: the photographs of **SeV^{2+}-NHC-PtNPs** and **SeV^{2+}-NHC-PtNPs'** in H_2O.

16. Femtosecond transient absorption measurements

Table S6: Time constants of multiple exponential fitting of femtosecond TA data of **3** and **SeV^{2+}-NHC-PtNPs**.

Compd	τ_1	τ_2	τ_3	τ_4
**3**	11.1	1.7326	1829	6379
**SeV^{2+}-NHC-PtNPs**	0.05	11.49	113.13	4111

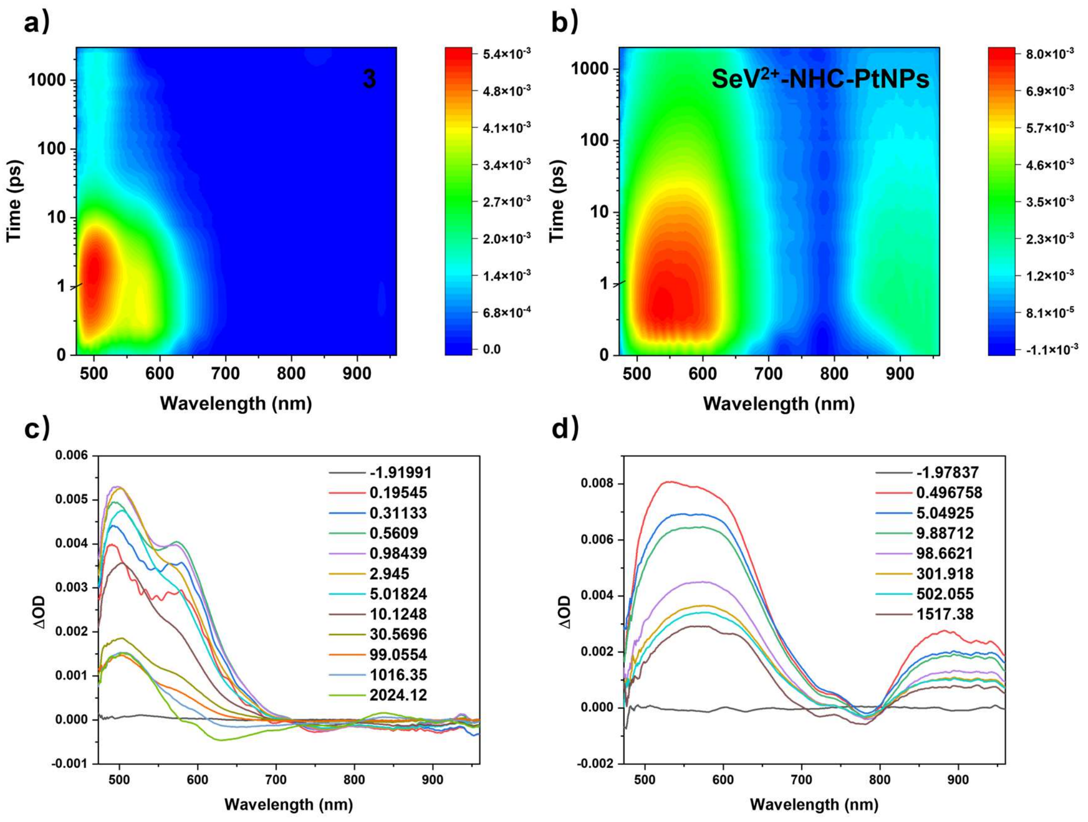

Figure S24: Transient absorption spectra of (a) (c) **3** in DMF and (b) (d) **SeV^{2+}-NHC-PtNPs** in DMSO.

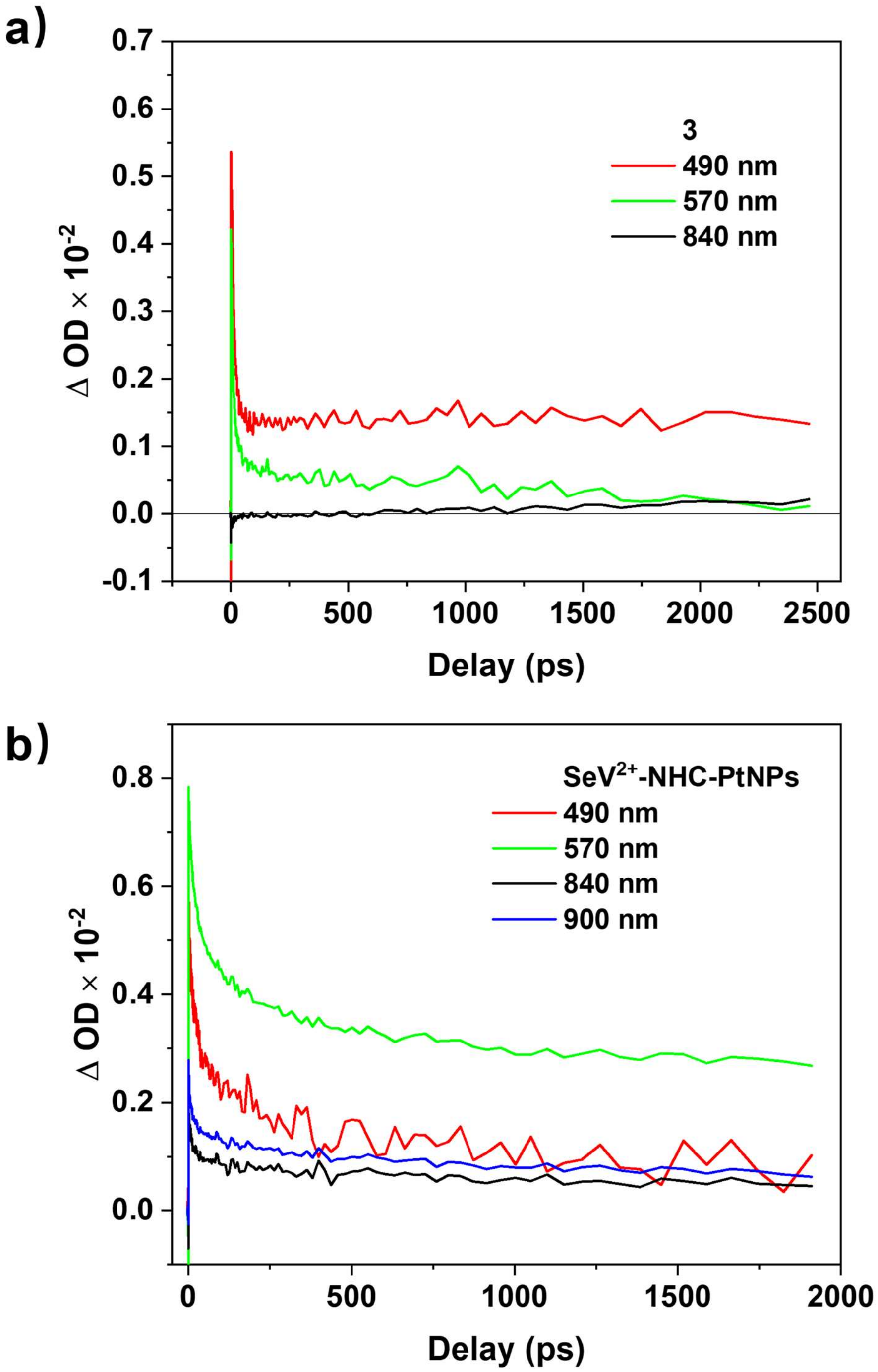

Figure S25: Decay curve in transient absorption. a, Decay curve in transient absorption of **3** at 490 nm, 570 nm and 840 nm. b, Decay curve in transient absorption of **SeV^{2+}-NHC-PtNPs** at 490 nm, 570 nm, 840 nm and 900 nm.

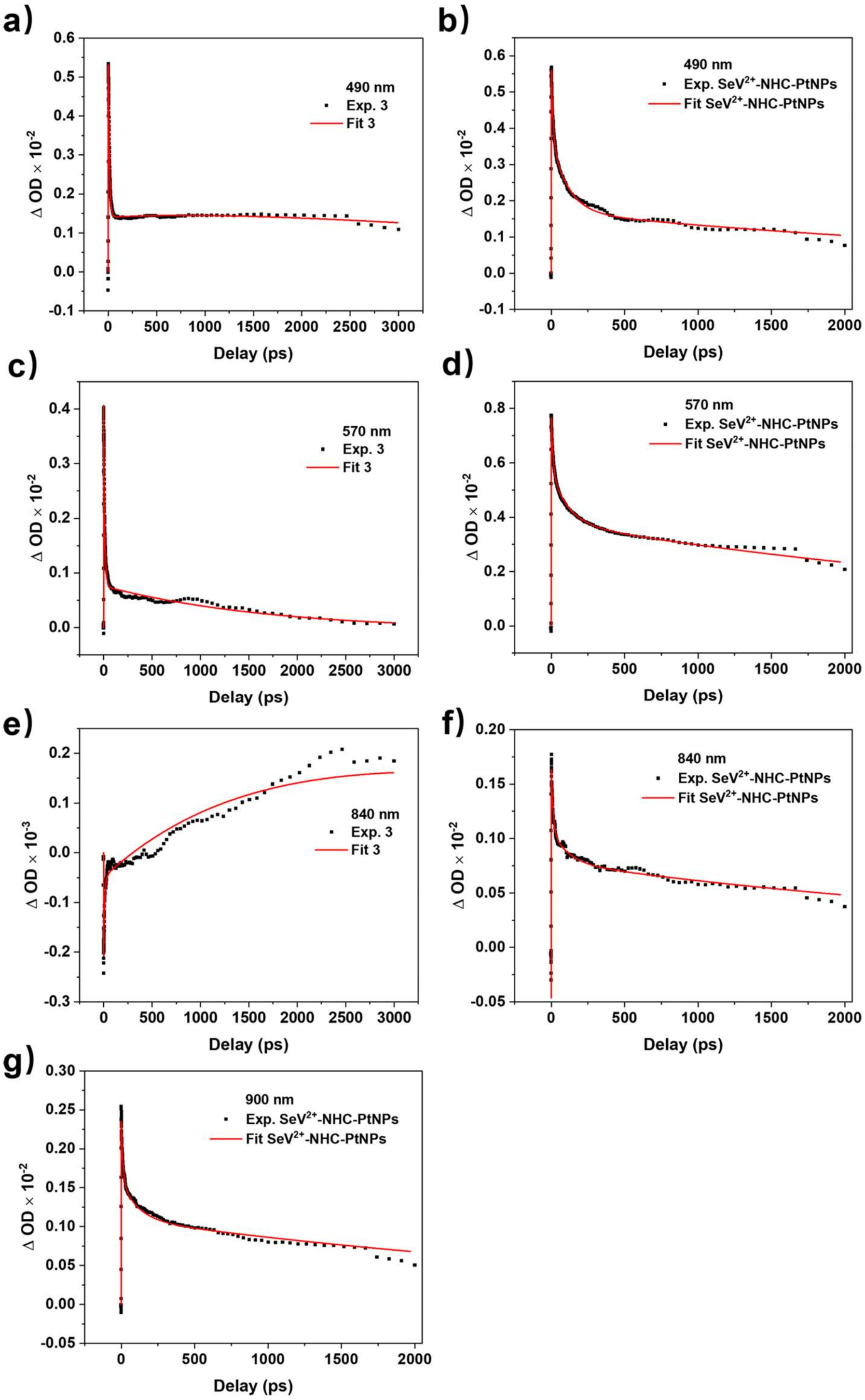

Figure S26: Kinetic traces. Kinetic traces of **3** probed at (a) 490 nm, (c) 570 nm and (e) 840 nm. Kinetic traces of **SeV^{2+}-NHC-PtNPs** probed at (b) 490 nm, (d) 570 nm, (f) 840 nm and (g) 900 nm.

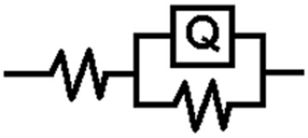

Figure S27: The conventional Randles circuit (R (QR)) procedure used for the measurement.

17. H_2 normalized curve

The system containing a mixture of **SeV^{2+}-NHC-PtNPs** (0.02 μmol), EDTP(0.15mmol), and 10 mL 0.1 M acetate buffer solution (0.03 M CH_3COOH and 0.07 M CH_3COONa, pH = 5.0) were sealed in a 20 mL Pyrex bottle. After bubbling with argon for 30 min away from light, 200 μL, 400 μL, 600 μL, 1000 μL, 6000 μL H_2 gas was injected to the different bottles respectively, followed by the injection of 200 μL CH_4 gas. Then 200 μL gas from these bottles was injected to GC respectively, every volume for three times. The curve figure was the peak area ratio (H_2/CH_4) verse the number of moles of H_2, and the equation was fitted by origin.

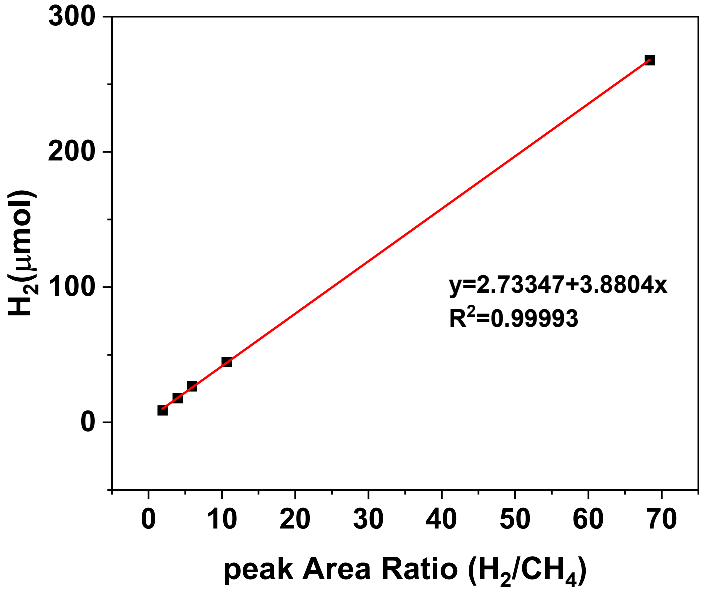

Figure S28: H_2 peak area versus the number of moles of hydrogen and polynomial fit of sample point to be H_2 normalized curve.

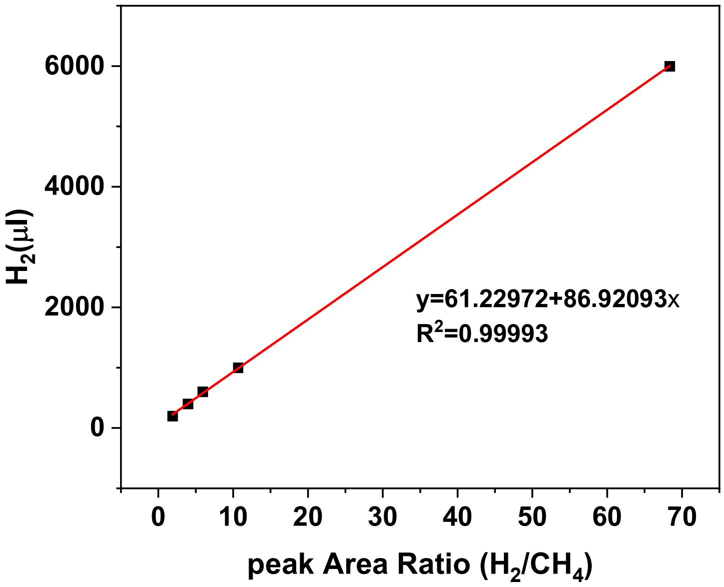

Figure S29. H_2 peak area versus the volume of hydrogen and polynomial fit of sample point to be H_2 normalized curve.

18. Hydrogen evolution under Xenon lamp

The solution was bubbled with argon for 30 min, after that it was irradiated under Xenon lamp (> 400 nm) with light power of 100 mW and 200 μL CH_4 was injected as the internal standard. Then 200 μL upper gas of the reactor was injected to gas chromatography per hour to measure H_2 evolution. The production of the H_2 was calculated according to the H_2 normalized curve.

Apparent quantum yield (AQY) calculation:

For the max of the absorption of **SeV^{2+}-NHC-PtNPs** is 397 nm, in the equation (3), λ = 397 nm. E is measured 100 mW. The number of incident photons (N_0) is calculated to be 1.99×10^{17} s^{-1} by equation (3). The H_2 molecules generated in 24 h under Xenon lamp was 128.98 μmol. The photons number of collected to be H_2 is 8.99×10^{14} s^{-1} as calculated by equation (21). The AQY is 0.9% calculated by equation (22):

$$N_0 = \lambda E / hc = 397 \times 10^{-9} \times 100 \times 10^{-3} / \left(6.63 \times 10^{-34} \times 3 \times 10^{8}\right) s^{-1} \quad (20)$$

$$N = nN_A / t = 128.98 \times 10^{-6} \times 6.02 \times 10^{23} / 24 \times 3600 s^{-1} = 8.99 \times 10^{14} s^{-1} \quad (21)$$

$$AQY_{SeV^{2+}\text{-}NHC\text{-}PtNPs} = 2N / N_0 \times 100\% = 0.9\% \quad (22)$$

Table S7: Hydrogen evolution activities of some organic photocatalytic system.

Classify	Catalysts	Hydrogen evolution rate (mmol•h^{-1} •g^{-1})	TON	Conditions	Reference
Three-component system	Thiocarbonyldye/SWCNT/C60; MV^{2+} (PT)[8]	0.098	7.6	Tris-HCl buffer	*J. Am. Chem.Soc.* **2018**, *140*, 3821-3824
	FS-COF/hexachloroplatinicm acid (5 μl, 8 wt. % aqueous solution)[9]	10.1		0.1 M ascorbic acid water solution	*Nat. Chem.* **2018**, *10*, 1180-1189

	OF and PTP/MV^{2+} /*Rhodopseudomonas palustris*[10]	1.63	0.84	phosphate-buffered saline solution	*J. Mater. Chem. A* ***2021***, *9*, 19788-19795
**Two-component system**	Tp-2C/BPy^{2+}-COF/3 wt.% Pt (405 µL 3.86 mM H_2PtCl_6 aqueous solution)[11]	34.6	415	0.1 M ascorbic acid water solution	*Angew. Chem. Int. Ed.* 2021, *60*, 9642-9649
	MPor-DETH-COFs/8 wt% H_2PtCl_6[12]	0.08-0.413		phosphate buffer solution	*Nat. Commun.* **2021**, *12*, 1354
	Py-Cb-SeV^{2+}/Pt-PVP[13]	1.91	44.5	0.1 M acetate buffer solution	*Adv. Sci.* **2022**, *9*, 2101652
	g-C_3N_4/*p*TA-*o*-TPV^{2+}/1 wt % Pt composite[14]	1.433		10vol% TEOA aqueous solution	*J. Am. Chem. Soc.* **2022**, *144*, 4422–4430
**One-component system**	*D. desulfuricans*-CdS[15]	0.036		0.02 M Tris-HCl buffer solution (pH=7.6)	*Angew. Chem.Int. Ed.* **2021**, *60*, 9055–9062
	PtL^{+}-SeV^{2+}[1]	0.312	58.9	0.1 M acetate buffer solution	*Angew. Chem.Int. Ed.* **2022**, *61*, e202115298
	TPCBP B-COF[16]	1.029		0.1 M sodium ascorbate buffer solution	*ACS Appl. Mater. Interfaces* **2023**, 15, 18836–18844
	PtSPs[17]	3.09		MeOH and H_2O	*Small* **2024**, 2400259
	CNT@Se-$CoTpy_{0.5}$[18]	2.47		10vol% TEA aqueous solution	*Carbon Energy*. **2025**, e70003
	Bimetallic Rh_2(II,II) complex[19]		170	0.03 M BNAH and 0.1 M TsOH	*Nat. Chem.* **2020**, 12, 180-185
	**SeV^{2+}-NHC-PtNPs**	2.71	169	0.1 M acetate buffer solution	*This work*

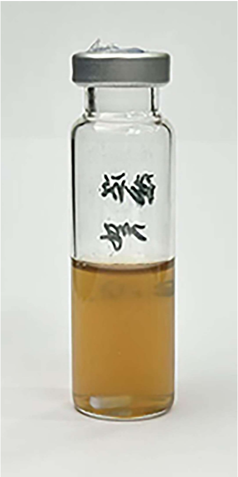

Figure S30. Photograph of the reaction solution after 5 successive cycles (30 hours) of continuous photocatalytic hydrogen evolution.

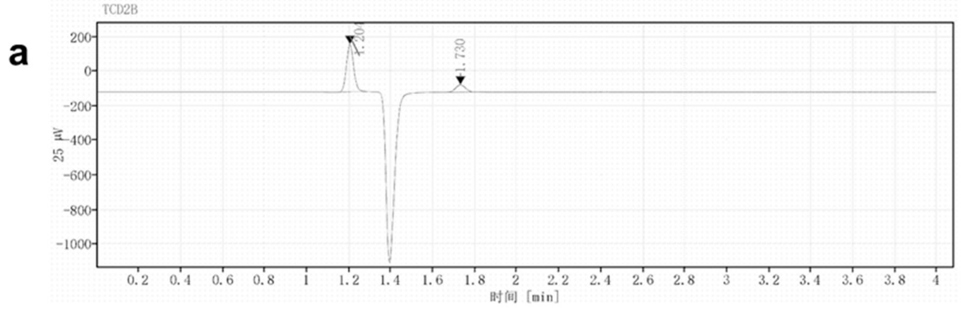

信号: TCD2B

保留时间 [min]	类型	峰宽 [min]	峰面积	高度	峰面积%	名称
1.204	MM m	0.04	658.80	275.32	82.97	peak@1.208min
1.730	MM m	0.05	135.26	41.57	17.03	
		总和	794.06			

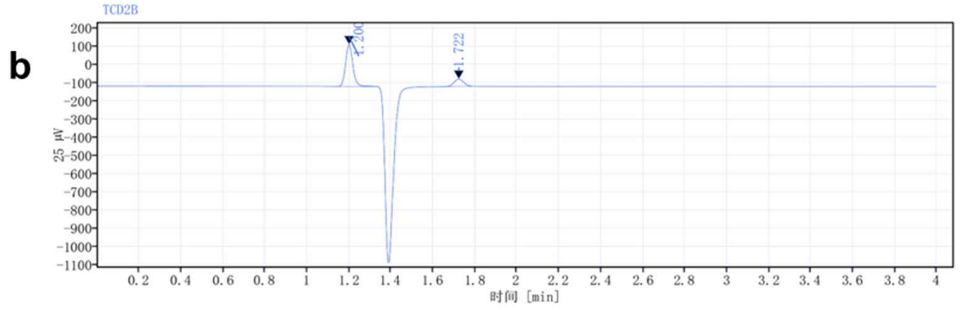

信号: TCD2B

保留时间 [min]	类型	峰宽 [min]	峰面积	高度	峰面积%	名称
1.200	MM m	0.04	559.16	230.77	81.11	peak@1.208min
1.722	MM m	0.05	130.25	40.63	18.89	
		总和	689.41			

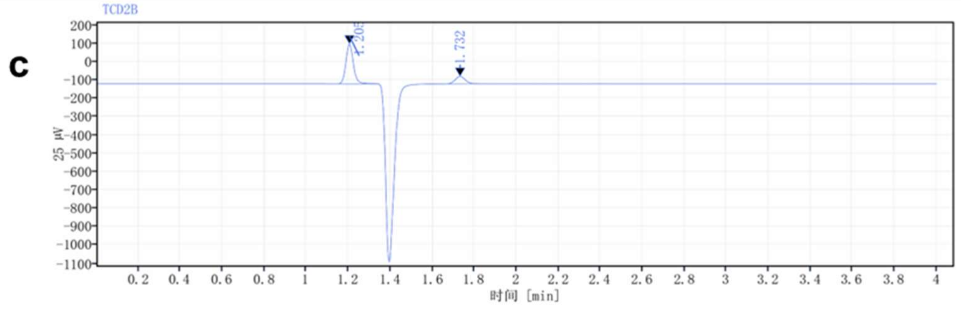

信号: TCD2B

保留时间 [min]	类型	峰宽 [min]	峰面积	高度	峰面积%	名称
1.205	MM m	0.04	536.85	216.82	80.42	peak@1.208min
1.732	MM m	0.05	130.74	39.87	19.58	
		总和	667.59			

Figure S31. Overlaid raw gas chromatography (GC) chromatograms of three parallel samples(a,b,c) after 2 hours of continuous irradiation.

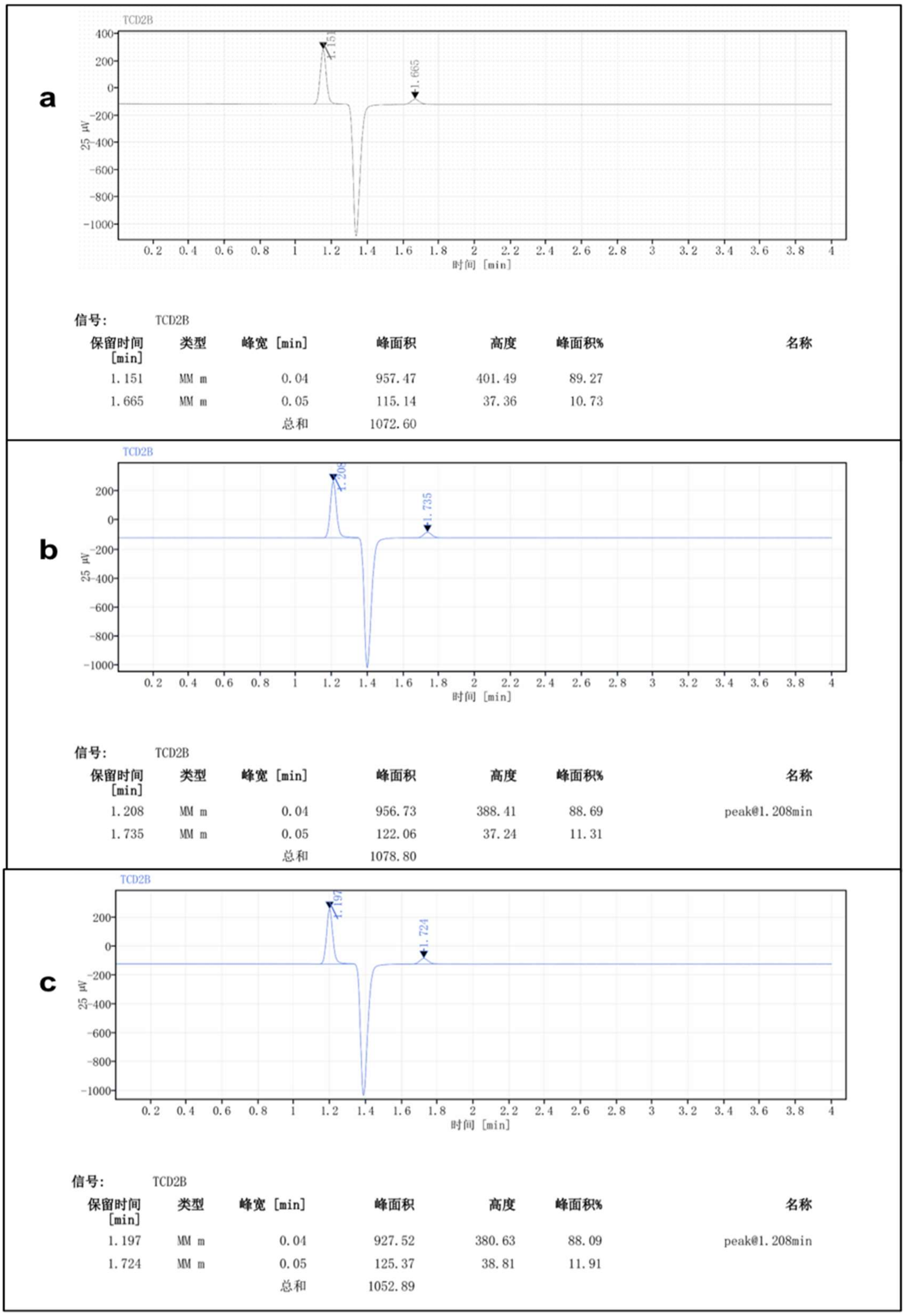

Figure S32. Overlaid raw gas chromatography (GC) chromatograms of three parallel samples(a,b,c) after 4 hours of continuous irradiation.

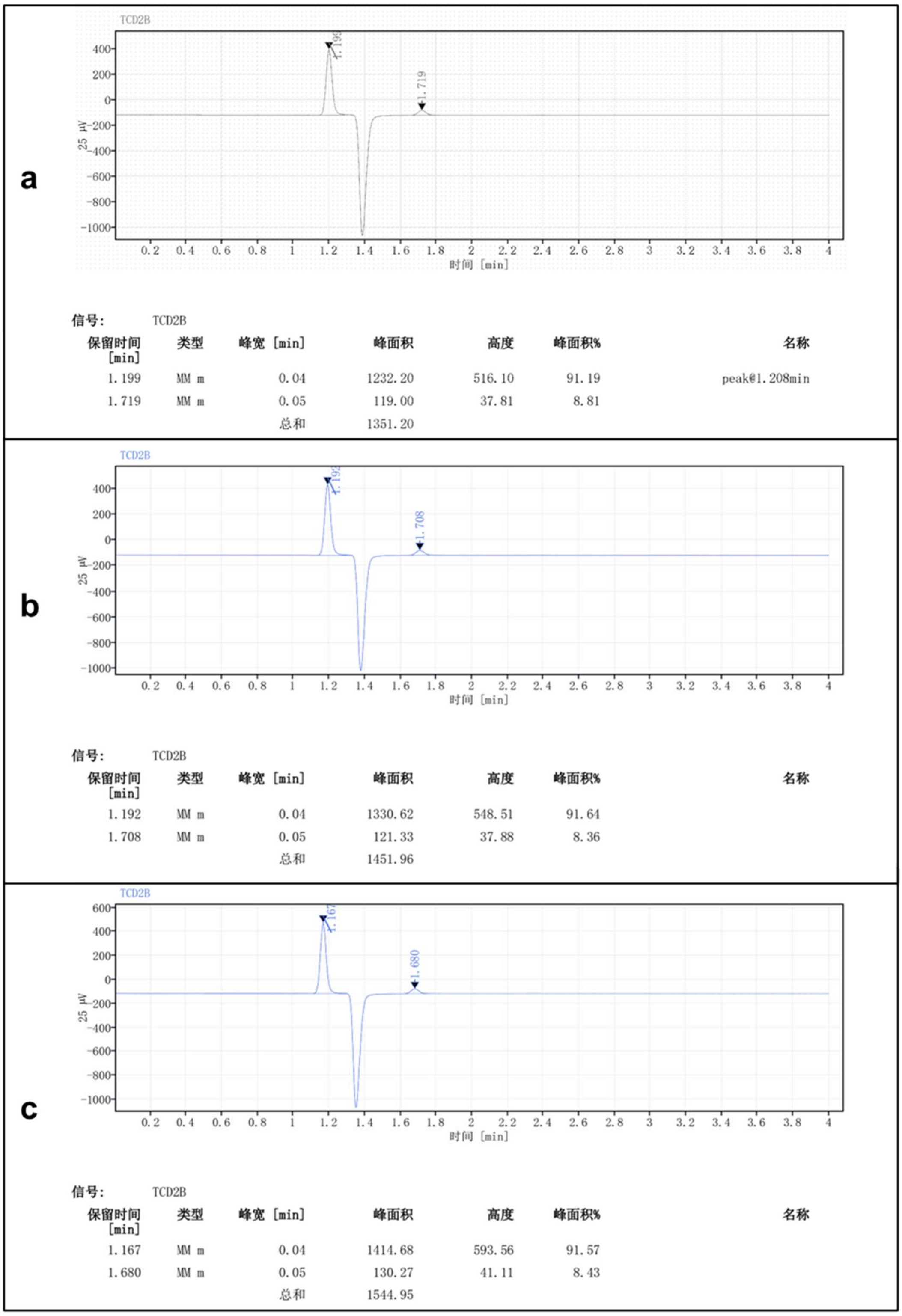

a

信号: TCD2B

保留时间 [min]	类型	峰宽 [min]	峰面积	高度	峰面积%	名称
1.199	MM m	0.04	1232.20	516.10	91.19	peak@1.208min
1.719	MM m	0.05	119.00	37.81	8.81	
		总和	1351.20			

b

信号: TCD2B

保留时间 [min]	类型	峰宽 [min]	峰面积	高度	峰面积%	名称
1.192	MM m	0.04	1330.62	548.51	91.64	
1.708	MM m	0.05	121.33	37.88	8.36	
		总和	1451.96			

c

信号: TCD2B

保留时间 [min]	类型	峰宽 [min]	峰面积	高度	峰面积%	名称
1.167	MM m	0.04	1414.68	593.56	91.57	
1.680	MM m	0.05	130.27	41.11	8.43	
		总和	1544.95			

Figure S33. Overlaid raw gas chromatography (GC) chromatograms of three parallel samples(a,b,c) after 6 hours of continuous irradiation.

a

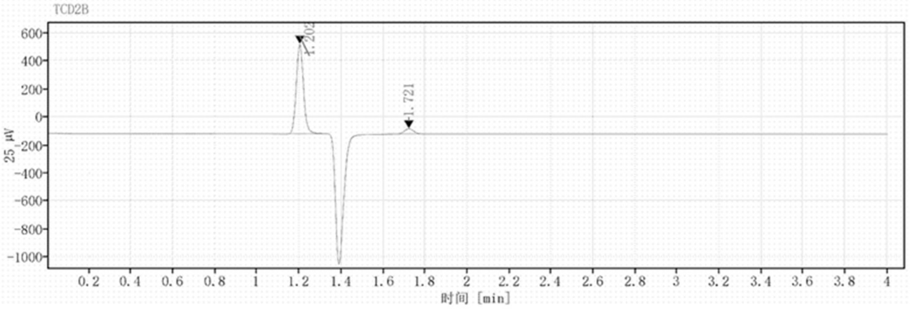

信号: TCD2B

保留时间 [min]	类型	峰宽 [min]	峰面积	高度	峰面积%	名称
1.202	MM m	0.04	1528.19	641.75	92.90	peak@1.208min
1.721	MM m	0.05	116.73	37.43	7.10	
		总和	1644.92			

b

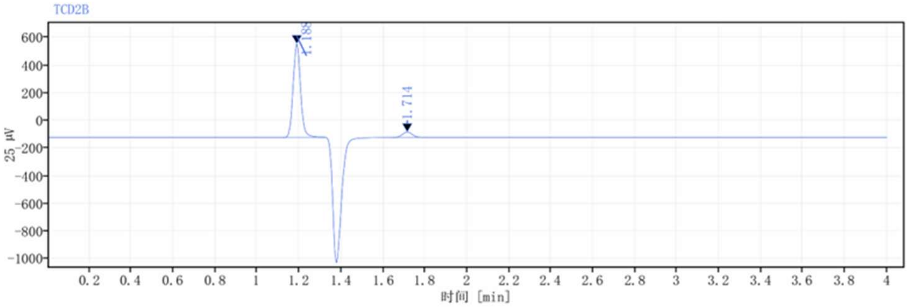

信号: TCD2B

保留时间 [min]	类型	峰宽 [min]	峰面积	高度	峰面积%	名称
1.188	MM m	0.04	1677.93	676.51	92.99	
1.714	MM m	0.05	126.55	38.82	7.01	
		总和	1804.49			

c

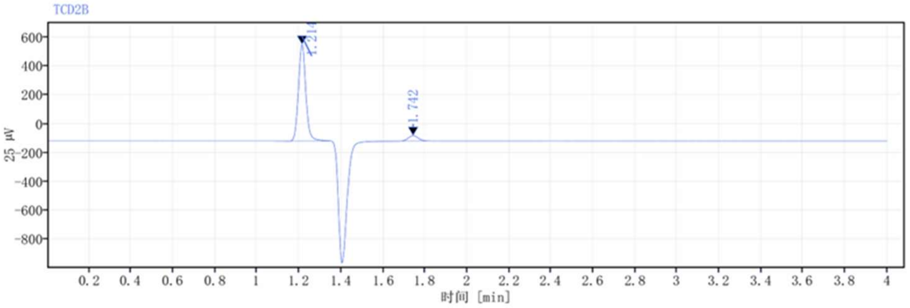

信号: TCD2B

保留时间 [min]	类型	峰宽 [min]	峰面积	高度	峰面积%	名称
1.214	MM m	0.04	1656.37	673.78	93.10	peak@1.208min
1.742	MM m	0.05	122.73	37.52	6.90	
		总和	1779.11			

Figure S34. Overlaid raw gas chromatography (GC) chromatograms of three parallel samples(a,b,c) after 8 hours of continuous irradiation.

a

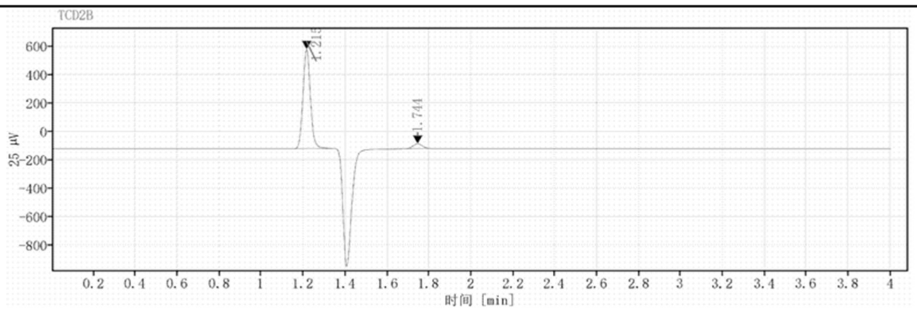

信号: TCD2B

保留时间 [min]	类型	峰宽 [min]	峰面积	高度	峰面积%	名称
1.215	MM m	0.04	1722.80	698.50	93.99	peak@1.208min
1.744	MM m	0.05	110.13	34.07	6.01	
		总和	1832.93			

b

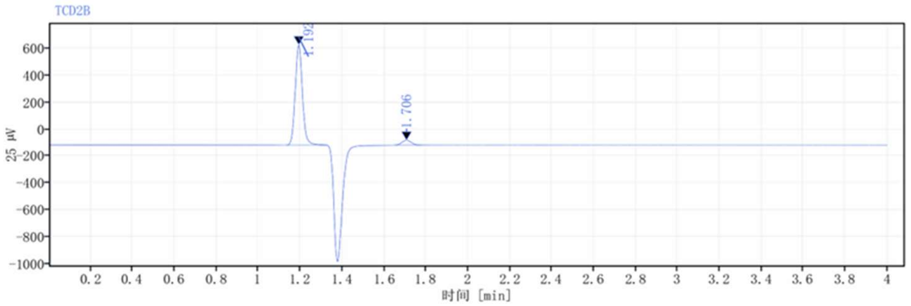

信号: TCD2B

保留时间 [min]	类型	峰宽 [min]	峰面积	高度	峰面积%	名称
1.192	MM m	0.04	1784.25	741.63	94.01	
1.706	MM m	0.05	113.68	36.44	5.99	
		总和	1897.93			

c

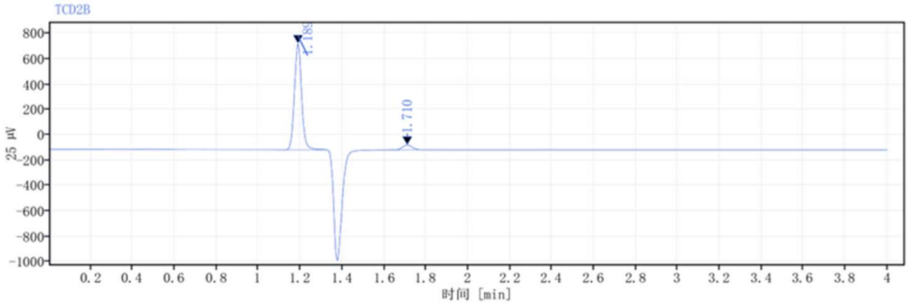

信号: TCD2B

保留时间 [min]	类型	峰宽 [min]	峰面积	高度	峰面积%	名称
1.189	MM m	0.04	2009.68	840.40	94.37	
1.710	MM m	0.05	120.01	38.06	5.63	
		总和	2129.68			

Figure S35. Overlaid raw gas chromatography (GC) chromatograms of three parallel samples(a,b,c) after 10 hours of continuous irradiation.

a

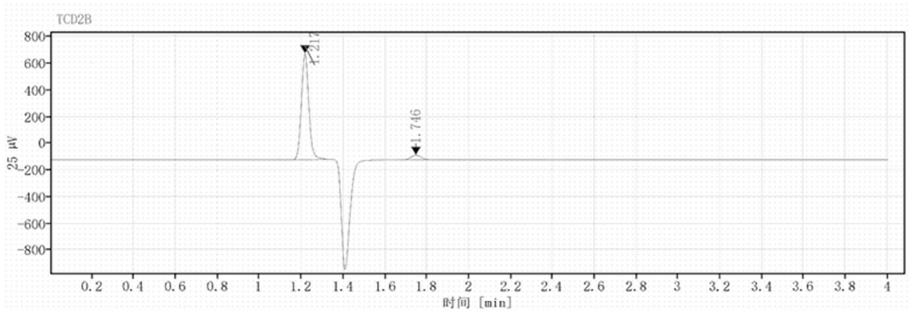

信号: TCD2B

保留时间 [min]	类型	峰宽 [min]	峰面积	高度	峰面积%	名称
1.217	MM m	0.04	1954.10	795.07	94.77	peak@1.208min
1.746	MM m	0.05	107.78	33.83	5.23	
		总和	2061.89			

b

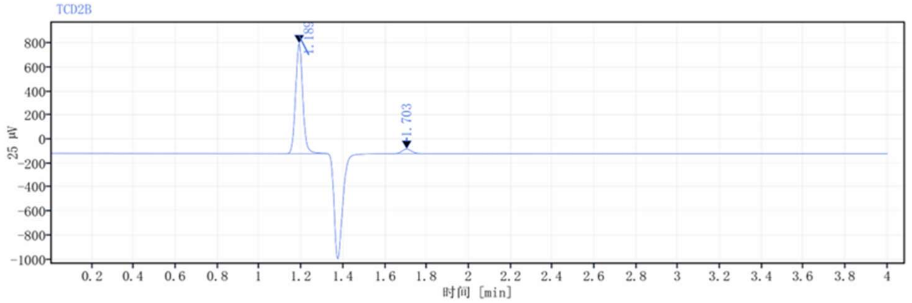

信号: TCD2B

保留时间 [min]	类型	峰宽 [min]	峰面积	高度	峰面积%	名称
1.189	MM m	0.04	2210.49	917.52	94.79	
1.703	MM m	0.05	121.61	37.88	5.21	
		总和	2332.10			

c

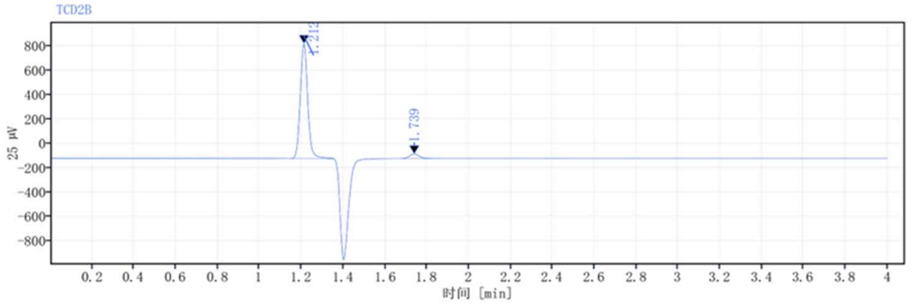

信号: TCD2B

保留时间 [min]	类型	峰宽 [min]	峰面积	高度	峰面积%	名称
1.212	MM m	0.04	2341.17	943.59	95.06	peak@1.208min
1.739	MM m	0.05	121.73	37.37	4.94	
		总和	2462.90			

Figure S36. Overlaid raw gas chromatography (GC) chromatograms of three parallel samples(a,b,c) after 12 hours of continuous irradiation.

a

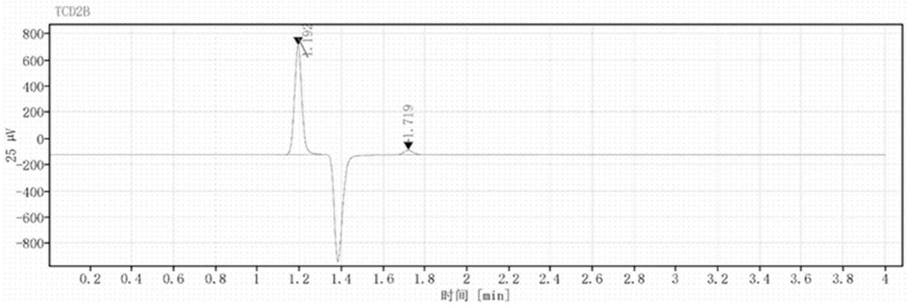

信号: TCD2B

保留时间 [min]	类型	峰宽 [min]	峰面积	高度	峰面积%	名称
1.192	MM m	0.04	2050.26	833.65	95.10	
1.719	MM m	0.05	105.67	33.41	4.90	
		总和	2155.92			

b

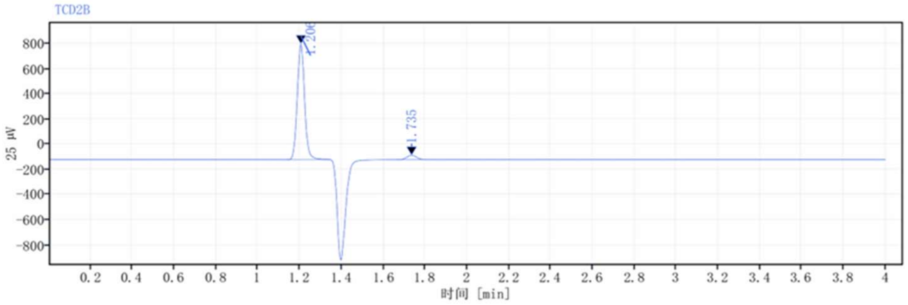

信号: TCD2B

保留时间 [min]	类型	峰宽 [min]	峰面积	高度	峰面积%	名称
1.206	MM m	0.04	2250.02	922.05	95.23	peak@1.208min
1.735	MM m	0.05	112.71	34.91	4.77	
		总和	2362.73			

c

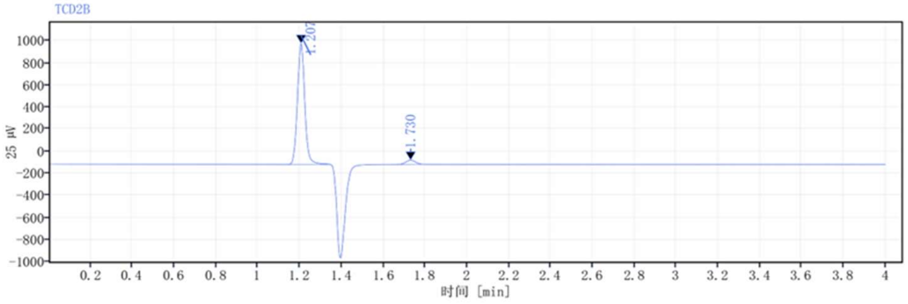

信号: TCD2B

保留时间 [min]	类型	峰宽 [min]	峰面积	高度	峰面积%	名称
1.207	MM m	0.04	2676.39	1097.19	95.69	peak@1.208min
1.730	MM m	0.05	120.50	38.00	4.31	
		总和	2796.89			

Figure S37. Overlaid raw gas chromatography (GC) chromatograms of three parallel samples(a,b,c) after 14 hours of continuous irradiation.

a

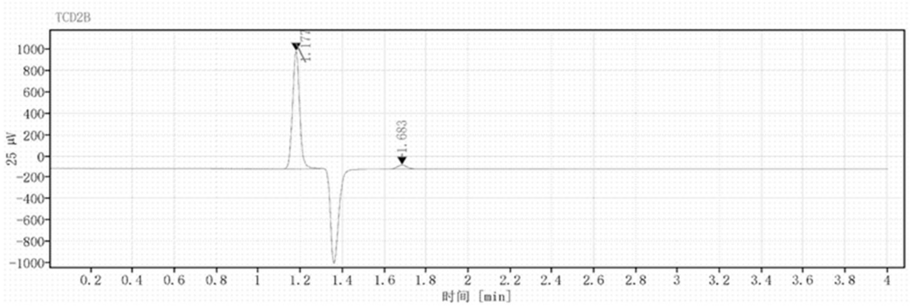

信号: TCD2B

保留时间 [min]	类型	峰宽 [min]	峰面积	高度	峰面积%	名称
1.177	MM m	0.04	2614.04	1106.31	95.87	
1.683	MM m	0.05	112.55	36.58	4.13	
		总和	2726.58			

b

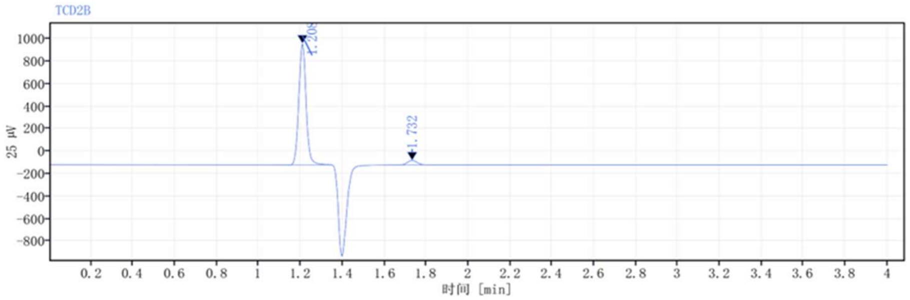

信号: TCD2B

保留时间 [min]	类型	峰宽 [min]	峰面积	高度	峰面积%	名称
1.208	MM m	0.04	2618.07	1080.62	95.50	peak@1.208min
1.732	MM m	0.05	123.50	39.63	4.50	
		总和	2741.57			

c

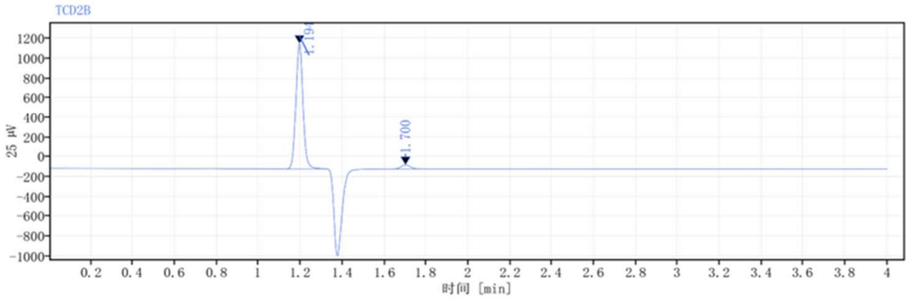

信号: TCD2B

保留时间 [min]	类型	峰宽 [min]	峰面积	高度	峰面积%	名称
1.194	MM m	0.04	2994.99	1268.79	96.12	
1.700	MM m	0.05	120.89	39.08	3.88	
		总和	3115.88			

Figure S38. Overlaid raw gas chromatography (GC) chromatograms of three parallel samples(a,b,c) after 16 hours of continuous irradiation.

a

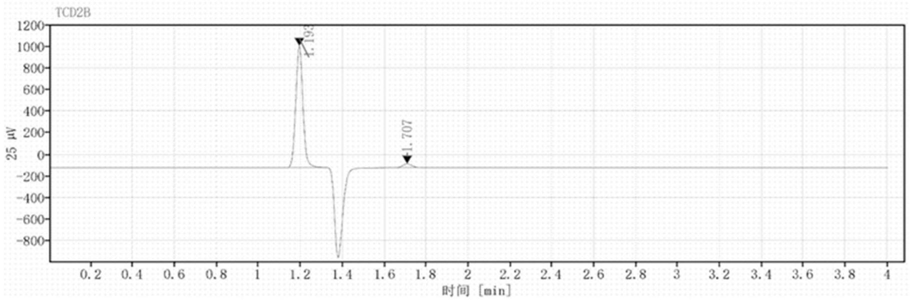

信号: TCD2B

保留时间 [min]	类型	峰宽 [min]	峰面积	高度	峰面积%	名称
1.193	MM m	0.04	2692.39	1123.79	96.08	
1.707	MM m	0.05	109.93	34.88	3.92	
		总和	2802.32			

b

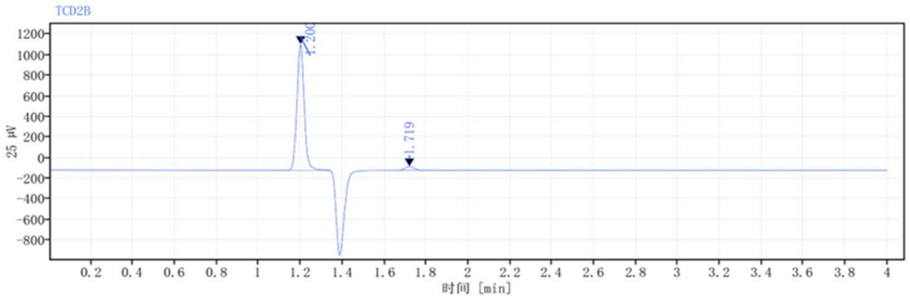

信号: TCD2B

保留时间 [min]	类型	峰宽 [min]	峰面积	高度	峰面积%	名称
1.200	MM m	0.04	2961.42	1223.71	96.40	peak@1.208min
1.719	MM m	0.05	110.65	35.64	3.60	
		总和	3072.06			

c

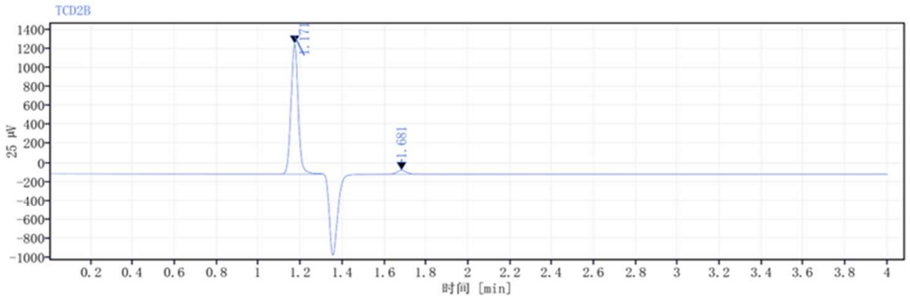

信号: TCD2B

保留时间 [min]	类型	峰宽 [min]	峰面积	高度	峰面积%	名称
1.171	MM m	0.04	3291.42	1373.86	96.46	
1.681	MM m	0.05	120.73	38.71	3.54	
		总和	3412.15			

Figure S39. Overlaid raw gas chromatography (GC) chromatograms of three parallel samples(a,b,c) after 18 hours of continuous irradiation.

a

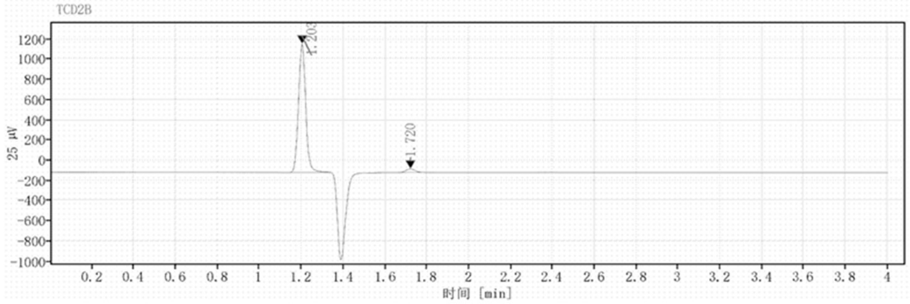

信号: TCD2B

保留时间 [min]	类型	峰宽 [min]	峰面积	高度	峰面积%	名称
1.203	MM m	0.04	3088.61	1275.01	96.52	peak@1.208min
1.720	MM m	0.05	111.43	35.76	3.48	
		总和	3200.04			

b

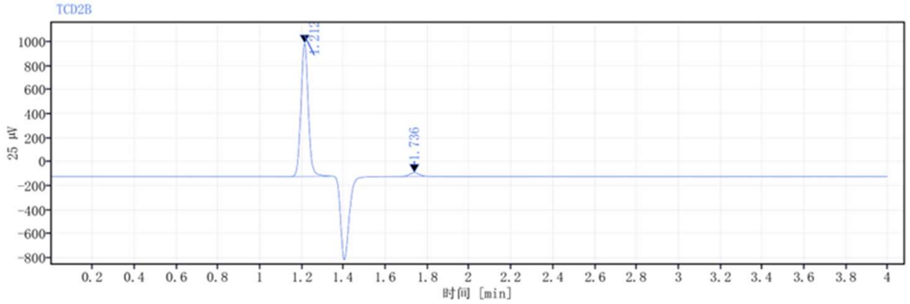

信号: TCD2B

保留时间 [min]	类型	峰宽 [min]	峰面积	高度	峰面积%	名称
1.212	MM m	0.04	2700.76	1107.44	96.59	peak@1.208min
1.736	MM m	0.05	95.42	30.54	3.41	
		总和	2796.18			

c

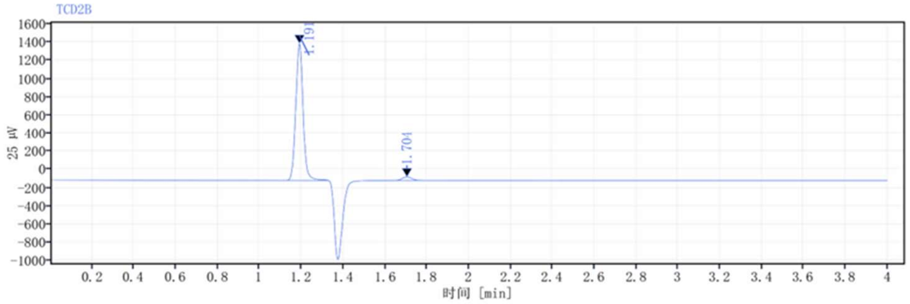

信号: TCD2B

保留时间 [min]	类型	峰宽 [min]	峰面积	高度	峰面积%	名称
1.191	MM m	0.04	3659.77	1506.23	96.70	
1.704	MM m	0.05	124.97	39.34	3.30	
		总和	3784.74			

Figure S40. Overlaid raw gas chromatography (GC) chromatograms of three parallel samples(a,b,c) after 20 hours of continuous irradiation.

a

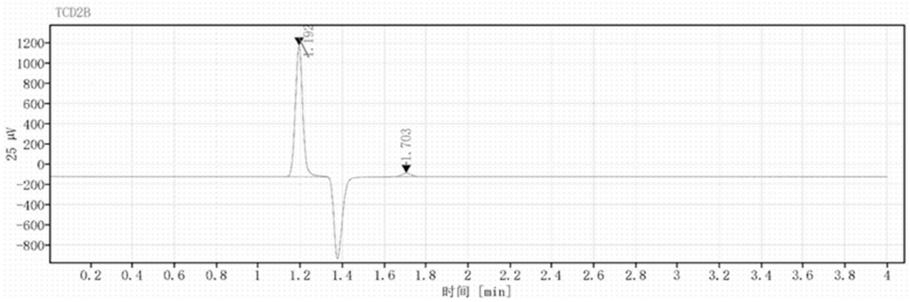

信号: TCD2B

保留时间 [min]	类型	峰宽 [min]	峰面积	高度	峰面积%	名称
1.192	MM m	0.04	3196.94	1296.90	96.68	
1.703	MM m	0.05	109.84	34.53	3.32	
		总和	3306.78			

b

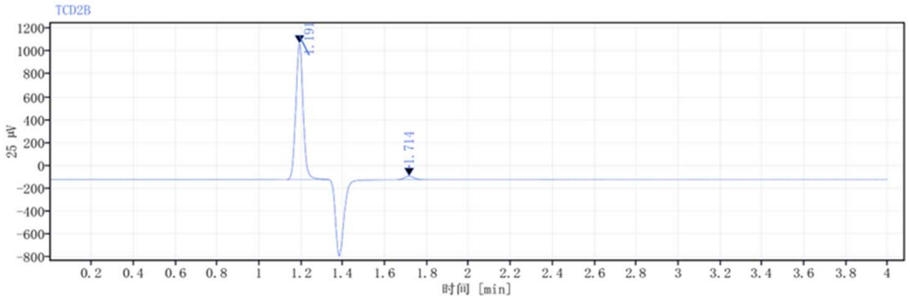

信号: TCD2B

保留时间 [min]	类型	峰宽 [min]	峰面积	高度	峰面积%	名称
1.191	MM m	0.04	2913.49	1189.49	96.99	
1.714	MM m	0.05	90.38	29.31	3.01	
		总和	3003.88			

c

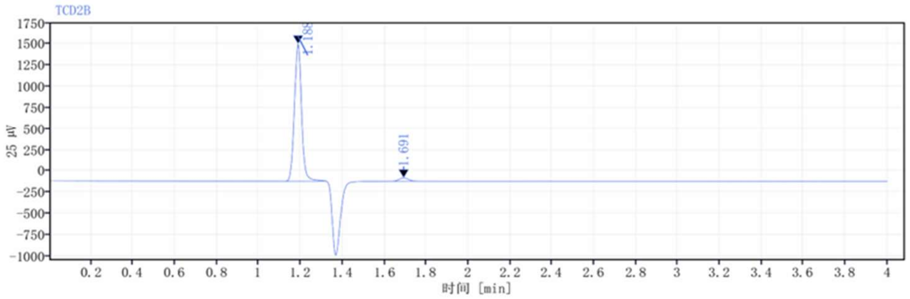

信号: TCD2B

保留时间 [min]	类型	峰宽 [min]	峰面积	高度	峰面积%	名称
1.188	MM m	0.04	3858.29	1623.21	97.00	
1.691	MM m	0.05	119.47	39.25	3.00	
		总和	3977.76			

Figure S41. Overlaid raw gas chromatography (GC) chromatograms of three parallel samples(a,b,c) after 22 hours of continuous irradiation.

a

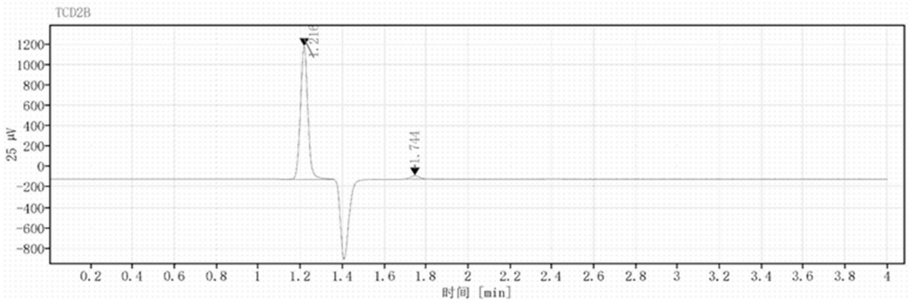

信号: TCD2B

保留时间 [min]	类型	峰宽 [min]	峰面积	高度	峰面积%	名称
1.216	MM m	0.04	3220.95	1302.55	96.89	peak@1.208min
1.744	MM m	0.05	103.51	32.89	3.11	
		总和	3324.46			

b

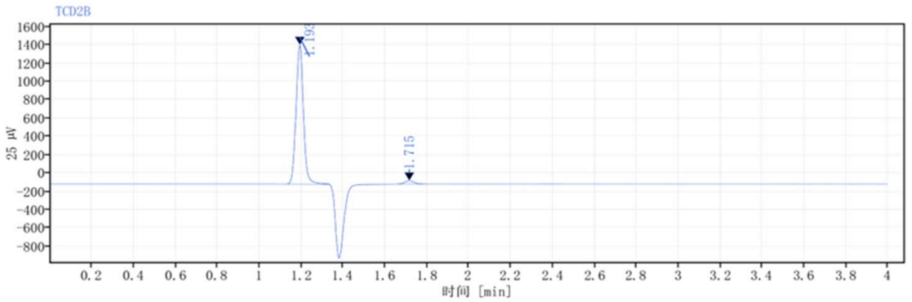

信号: TCD2B

保留时间 [min]	类型	峰宽 [min]	峰面积	高度	峰面积%	名称
1.193	MM m	0.04	3746.06	1520.09	97.03	
1.715	MM m	0.05	114.63	36.23	2.97	
		总和	3860.69			

c

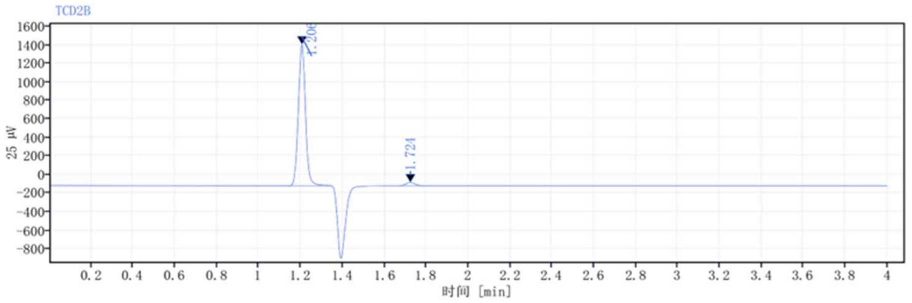

信号: TCD2B

保留时间 [min]	类型	峰宽 [min]	峰面积	高度	峰面积%	名称
1.206	MM m	0.04	3608.21	1522.89	97.13	peak@1.208min
1.724	MM m	0.05	106.74	34.86	2.87	
		总和	3714.95			

Figure S42. Overlaid raw gas chromatography (GC) chromatograms of three parallel samples(a,b,c) after 24 hours of continuous irradiation.

19. Cartesian coordinates of optimized structures studied in this work

3

Symbol	X	Y	Z
C	-0.915	0.262	-0.23
C	-2.178	0.058	-0.655
C	-3.033	1.093	-0.746
C	-2.52	2.286	-0.391
C	-1.253	2.424	0.023
C	-4.331	1.075	-1.137
C	-5.06	0.015	-1.528
C	-6.351	0.182	-1.875
N	-6.994	1.323	-1.868
C	-6.279	2.353	-1.492
C	-4.991	2.25	-1.135
Se	-3.839	3.692	-0.556
C	-8.456	1.431	-2.265
C	-9.317	0.938	-1.124
C	-9.896	-0.276	-1.163
C	-10.658	-0.709	-0.146
C	-10.851	0.072	0.928
C	-10.279	1.285	0.978
C	-9.519	1.711	-0.042
C	1.006	1.648	0.615
C	1.975	0.501	0.42
C	2.268	-0.343	1.425

**C**	3.162	-1.33	1.263
**C**	3.798	-1.505	0.091
**C**	3.499	-0.667	-0.917
**C**	2.604	0.32	-0.755
**C**	4.814	-2.614	-0.077
**C**	6.604	-2.566	1.702
**N**	6.129	-2.213	0.539
**C**	7.047	-1.439	0.019
**C**	8.081	-1.315	0.864
**N**	7.738	-2.023	1.855
**C**	8.556	-2.206	3.061
**H**	-0.249	-0.614	-0.16
**H**	-2.488	-0.966	-0.917
**H**	-0.884	3.422	0.31
**H**	-4.628	-0.998	-1.57
**H**	-6.928	-0.704	-2.187
**H**	-6.774	3.337	-1.467
**H**	-8.727	2.483	-2.515
**H**	-8.637	0.848	-3.197
**H**	-9.746	-0.932	-2.036
**H**	-11.125	-1.707	-0.191
**H**	-11.475	-0.282	1.766
**H**	-10.433	1.93	1.859
**H**	-9.053	2.708	0.013
**H**	1.431	2.544	0.104
**H**	0.955	1.915	1.697

**H**	1.776	-0.222	2.405
**H**	3.387	-2	2.11
**H**	4.003	-0.78	-1.892
**H**	2.391	0.999	-1.597
**H**	4.438	-3.548	0.4
**H**	4.969	-2.858	-1.153
**H**	6.13	-3.215	2.445
**H**	6.957	-0.969	-0.968
**H**	9.018	-0.755	0.778
**H**	9.504	-1.624	3.014
**H**	7.995	-1.868	3.963
**H**	8.822	-3.28	3.188

3'

Symbol	X	Y	Z
**C**	-0.915	0.262	-0.23
**C**	-2.178	0.058	-0.655
**C**	-3.033	1.093	-0.746
**C**	-2.52	2.286	-0.391
**C**	-1.253	2.424	0.023
**C**	-4.331	1.075	-1.137
**C**	-5.06	0.015	-1.528
**C**	-6.351	0.182	-1.875
**N**	-6.994	1.323	-1.868
**C**	-6.279	2.353	-1.492
**C**	-4.991	2.25	-1.135
**Se**	-3.839	3.692	-0.556

**C**	-8.456	1.431	-2.265
**C**	-9.317	0.938	-1.124
**C**	-9.896	-0.276	-1.163
**C**	-10.658	-0.709	-0.146
**C**	-10.851	0.072	0.928
**C**	-10.279	1.285	0.978
**C**	-9.519	1.711	-0.042
**C**	1.006	1.648	0.615
**C**	1.975	0.501	0.42
**C**	2.268	-0.343	1.425
**C**	3.162	-1.33	1.263
**C**	3.798	-1.505	0.091
**C**	3.499	-0.667	-0.917
**C**	2.604	0.32	-0.755
**C**	4.814	-2.614	-0.077
**C**	6.604	-2.566	1.702
**N**	6.129	-2.213	0.539
**C**	7.047	-1.439	0.019
**C**	8.081	-1.315	0.864
**N**	7.738	-2.023	1.855
**C**	8.556	-2.206	3.061
**H**	-0.249	-0.614	-0.16
**H**	-2.488	-0.966	-0.917
**H**	-0.884	3.422	0.31
**H**	-4.628	-0.998	-1.57
**H**	-6.928	-0.704	-2.187

**H**	-6.774	3.337	-1.467
**H**	-8.727	2.483	-2.515
**H**	-8.637	0.848	-3.197
**H**	-9.746	-0.932	-2.036
**H**	-11.125	-1.707	-0.191
**H**	-11.475	-0.282	1.766
**H**	-10.433	1.93	1.859
**H**	-9.053	2.708	0.013
**H**	1.431	2.544	0.104
**H**	0.955	1.915	1.697
**H**	1.776	-0.222	2.405
**H**	3.387	-2	2.11
**H**	4.003	-0.78	-1.892
**H**	2.391	0.999	-1.597
**H**	4.438	-3.548	0.4
**H**	4.969	-2.858	-1.153
**H**	6.13	-3.215	2.445
**H**	6.957	-0.969	-0.968
**H**	9.018	-0.755	0.778
**H**	9.504	-1.624	3.014
**H**	7.995	-1.868	3.963
**H**	8.822	-3.28	3.188

3’’

Symbol	X	Y	Z
**C**	-0.915	0.262	-0.23
**C**	-2.178	0.058	-0.655

C	-3.033	1.093	-0.746
C	-2.52	2.286	-0.391
C	-1.253	2.424	0.023
C	-4.331	1.075	-1.137
C	-5.06	0.015	-1.528
C	-6.351	0.182	-1.875
N	-6.994	1.323	-1.868
C	-6.279	2.353	-1.492
C	-4.991	2.25	-1.135
Se	-3.839	3.692	-0.556
C	-8.456	1.431	-2.265
C	-9.317	0.938	-1.124
C	-9.896	-0.276	-1.163
C	-10.658	-0.709	-0.146
C	-10.851	0.072	0.928
C	-10.279	1.285	0.978
C	-9.519	1.711	-0.042
C	1.006	1.648	0.615
C	1.975	0.501	0.42
C	2.268	-0.343	1.425
C	3.162	-1.33	1.263
C	3.798	-1.505	0.091
C	3.499	-0.667	-0.917
C	2.604	0.32	-0.755
C	4.814	-2.614	-0.077
C	6.604	-2.566	1.702

**N**	6.129	-2.213	0.539
**C**	7.047	-1.439	0.019
**C**	8.081	-1.315	0.864
**N**	7.738	-2.023	1.855
**C**	8.556	-2.206	3.061
**H**	-0.249	-0.614	-0.16
**H**	-2.488	-0.966	-0.917
**H**	-0.884	3.422	0.31
**H**	-4.628	-0.998	-1.57
**H**	-6.928	-0.704	-2.187
**H**	-6.774	3.337	-1.467
**H**	-8.727	2.483	-2.515
**H**	-8.637	0.848	-3.197
**H**	-9.746	-0.932	-2.036
**H**	-11.125	-1.707	-0.191
**H**	-11.475	-0.282	1.766
**H**	-10.433	1.93	1.859
**H**	-9.053	2.708	0.013
**H**	1.431	2.544	0.104
**H**	0.955	1.915	1.697
**H**	1.776	-0.222	2.405
**H**	3.387	-2	2.11
**H**	4.003	-0.78	-1.892
**H**	2.391	0.999	-1.597
**H**	4.438	-3.548	0.4
**H**	4.969	-2.858	-1.153

H	6.13	-3.215	2.445
H	6.957	-0.969	-0.968
H	9.018	-0.755	0.778
H	9.504	-1.624	3.014
H	7.995	-1.868	3.963
H	8.822	-3.28	3.188

SeV^{2+}-NHC-PtNPs

Symbol	X	Y	Z
N	0.20124	-0.71207	0
C	0.23323	-2.06976	0.09141
C	-0.86707	-2.82667	-0.24303
C	-2.02657	-2.18059	-0.6924
C	-2.02549	-0.76971	-0.78083
C	-0.89164	-0.05344	-0.4234
C	-3.27923	-2.77807	-1.10408
C	-3.61989	-4.13582	-1.14365
C	-4.87716	-4.49742	-1.57732
N	-5.78283	-3.55799	-1.96102
C	-5.49534	-2.24503	-1.93693
C	-4.24348	-1.8279	-1.5114
Se	-3.64868	-0.04052	-1.40718
C	-7.12639	-4.02476	-2.47463
C	-8.22624	-3.03818	-2.2043
C	-8.72661	-2.23861	-3.2385
C	-9.73888	-1.31188	-2.9817
C	-10.2505	-1.17928	-1.69001

**C**	-9.75299	-1.97575	-0.65373
**C**	-8.74551	-2.90349	-0.90912
**C**	1.441	0.05261	0.3658
**C**	2.58676	-0.31333	-0.5425
**C**	3.67998	-1.03332	-0.04643
**C**	4.73045	-1.38617	-0.89142
**C**	4.70032	-1.02781	-2.24408
**C**	3.60829	-0.30479	-2.73563
**C**	2.55792	0.05139	-1.89285
**C**	5.83706	-1.41167	-3.16768
**C**	8.06472	-1.4798	-2.01322
**N**	7.11323	-0.83139	-2.74591
**C**	7.46508	0.49867	-2.9351
**C**	8.66224	0.67846	-2.32339
**N**	9.01578	-0.539	-1.7574
**C**	10.26055	-0.7895	-1.03869
**H**	1.16429	-2.50373	0.42805
**H**	-0.81045	-3.90428	-0.15478
**H**	-0.82927	1.02548	-0.46617
**H**	-2.926	-4.90963	-0.83996
**H**	-5.20095	-5.52757	-1.63377
**H**	-6.27819	-1.57092	-2.25566
**H**	-6.99452	-4.20378	-3.54392
**H**	-7.3098	-4.97875	-1.97958
**H**	-8.32583	-2.34414	-4.24277
**H**	-10.1253	-0.69751	-3.7888

**H**	-11.0378	-0.45892	-1.48948
**H**	-10.1538	-1.87639	0.3502
**H**	-8.35965	-3.52534	-0.10611
**H**	1.18163	1.10928	0.29874
**H**	1.65837	-0.18228	1.40892
**H**	3.71163	-1.31571	1.00222
**H**	5.57921	-1.94055	-0.49721
**H**	3.58136	-0.01461	-3.78189
**H**	1.71938	0.61763	-2.2886
**H**	5.97027	-2.4967	-3.1967
**H**	5.63515	-1.07557	-4.18706
**H**	6.83545	1.18345	-3.47999
**H**	9.29282	1.54919	-2.23991
**H**	10.522	0.09676	-0.45822
**H**	10.10906	-1.63113	-0.36113
**H**	11.06772	-1.022	-1.73832
**Pt**	8.05842	-3.32209	-1.45003

SeV^{2+}-NHC-PtNPs'

Symbol	X	Y	Z
**N**	0.20124	-0.71207	0
**C**	0.23323	-2.06976	0.09141
**C**	-0.86707	-2.82667	-0.24303
**C**	-2.02657	-2.18059	-0.6924
**C**	-2.02549	-0.76971	-0.78083
**C**	-0.89164	-0.05344	-0.4234
**C**	-3.27923	-2.77807	-1.10408

**C**	-3.61989	-4.13582	-1.14365
**C**	-4.87716	-4.49742	-1.57732
**N**	-5.78283	-3.55799	-1.96102
**C**	-5.49534	-2.24503	-1.93693
**C**	-4.24348	-1.8279	-1.5114
**Se**	-3.64868	-0.04052	-1.40718
**C**	-7.12639	-4.02476	-2.47463
**C**	-8.22624	-3.03818	-2.2043
**C**	-8.72661	-2.23861	-3.2385
**C**	-9.73888	-1.31188	-2.9817
**C**	-10.2505	-1.17928	-1.69001
**C**	-9.75299	-1.97575	-0.65373
**C**	-8.74551	-2.90349	-0.90912
**C**	1.441	0.05261	0.3658
**C**	2.58676	-0.31333	-0.5425
**C**	3.67998	-1.03332	-0.04643
**C**	4.73045	-1.38617	-0.89142
**C**	4.70032	-1.02781	-2.24408
**C**	3.60829	-0.30479	-2.73563
**C**	2.55792	0.05139	-1.89285
**C**	5.83706	-1.41167	-3.16768
**C**	8.06472	-1.4798	-2.01322
**N**	7.11323	-0.83139	-2.74591
**C**	7.46508	0.49867	-2.9351
**C**	8.66224	0.67846	-2.32339
**N**	9.01578	-0.539	-1.7574

**C**	10.26055	-0.7895	-1.03869
**H**	1.16429	-2.50373	0.42805
**H**	-0.81045	-3.90428	-0.15478
**H**	-0.82927	1.02548	-0.46617
**H**	-2.926	-4.90963	-0.83996
**H**	-5.20095	-5.52757	-1.63377
**H**	-6.27819	-1.57092	-2.25566
**H**	-6.99452	-4.20378	-3.54392
**H**	-7.3098	-4.97875	-1.97958
**H**	-8.32583	-2.34414	-4.24277
**H**	-10.1253	-0.69751	-3.7888
**H**	-11.0378	-0.45892	-1.48948
**H**	-10.1538	-1.87639	0.3502
**H**	-8.35965	-3.52534	-0.10611
**H**	1.18163	1.10928	0.29874
**H**	1.65837	-0.18228	1.40892
**H**	3.71163	-1.31571	1.00222
**H**	5.57921	-1.94055	-0.49721
**H**	3.58136	-0.01461	-3.78189
**H**	1.71938	0.61763	-2.2886
**H**	5.97027	-2.4967	-3.1967
**H**	5.63515	-1.07557	-4.18706
**H**	6.83545	1.18345	-3.47999
**H**	9.29282	1.54919	-2.23991
**H**	10.522	0.09676	-0.45822
**H**	10.10906	-1.63113	-0.36113

**H**	11.06772	-1.022	-1.73832
**Pt**	8.05842	-3.32209	-1.45003

SeV^{2+}-NHC-PtNPs''

Symbol	X	Y	Z
**N**	0.20124	-0.71207	0
**C**	0.23323	-2.06976	0.09141
**C**	-0.86707	-2.82667	-0.24303
**C**	-2.02657	-2.18059	-0.6924
**C**	-2.02549	-0.76971	-0.78083
**C**	-0.89164	-0.05344	-0.4234
**C**	-3.27923	-2.77807	-1.10408
**C**	-3.61989	-4.13582	-1.14365
**C**	-4.87716	-4.49742	-1.57732
**N**	-5.78283	-3.55799	-1.96102
**C**	-5.49534	-2.24503	-1.93693
**C**	-4.24348	-1.8279	-1.5114
**Se**	-3.64868	-0.04052	-1.40718
**C**	-7.12639	-4.02476	-2.47463
**C**	-8.22624	-3.03818	-2.2043
**C**	-8.72661	-2.23861	-3.2385
**C**	-9.73888	-1.31188	-2.9817
**C**	-10.2505	-1.17928	-1.69001
**C**	-9.75299	-1.97575	-0.65373
**C**	-8.74551	-2.90349	-0.90912
**C**	1.441	0.05261	0.3658
**C**	2.58676	-0.31333	-0.5425

**C**	3.67998	-1.03332	-0.04643
**C**	4.73045	-1.38617	-0.89142
**C**	4.70032	-1.02781	-2.24408
**C**	3.60829	-0.30479	-2.73563
**C**	2.55792	0.05139	-1.89285
**C**	5.83706	-1.41167	-3.16768
**C**	8.06472	-1.4798	-2.01322
**N**	7.11323	-0.83139	-2.74591
**C**	7.46508	0.49867	-2.9351
**C**	8.66224	0.67846	-2.32339
**N**	9.01578	-0.539	-1.7574
**C**	10.26055	-0.7895	-1.03869
**H**	1.16429	-2.50373	0.42805
**H**	-0.81045	-3.90428	-0.15478
**H**	-0.82927	1.02548	-0.46617
**H**	-2.926	-4.90963	-0.83996
**H**	-5.20095	-5.52757	-1.63377
**H**	-6.27819	-1.57092	-2.25566
**H**	-6.99452	-4.20378	-3.54392
**H**	-7.3098	-4.97875	-1.97958
**H**	-8.32583	-2.34414	-4.24277
**H**	-10.1253	-0.69751	-3.7888
**H**	-11.0378	-0.45892	-1.48948
**H**	-10.1538	-1.87639	0.3502
**H**	-8.35965	-3.52534	-0.10611
**H**	1.18163	1.10928	0.29874

**H**	1.65837	-0.18228	1.40892
**H**	3.71163	-1.31571	1.00222
**H**	5.57921	-1.94055	-0.49721
**H**	3.58136	-0.01461	-3.78189
**H**	1.71938	0.61763	-2.2886
**H**	5.97027	-2.4967	-3.1967
**H**	5.63515	-1.07557	-4.18706
**H**	6.83545	1.18345	-3.47999
**H**	9.29282	1.54919	-2.23991
**H**	10.522	0.09676	-0.45822
**H**	10.10906	-1.63113	-0.36113
**H**	11.06772	-1.022	-1.73832
**Pt**	8.05842	-3.32209	-1.45003

20. ^{1}H, ^{13}C, and high-resolution mass spectrum (HRMS)

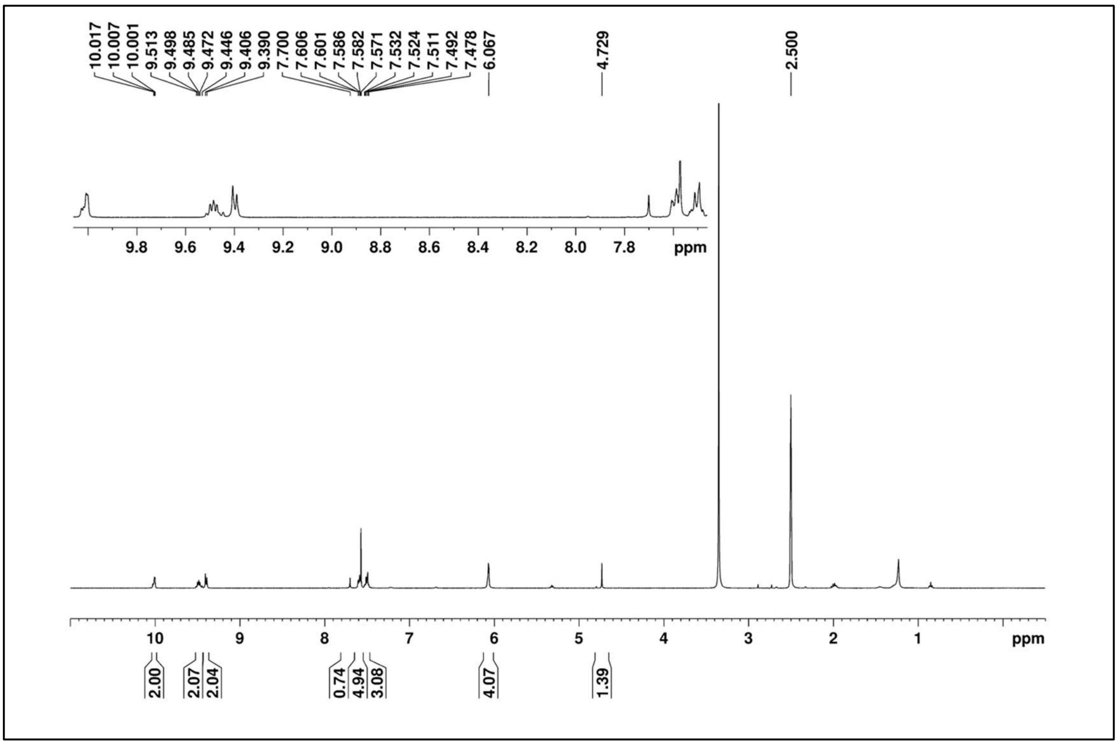

Figure S43: ^{1}H NMR (DMSO-d$_6$, 400 MHz) spectra of **2**

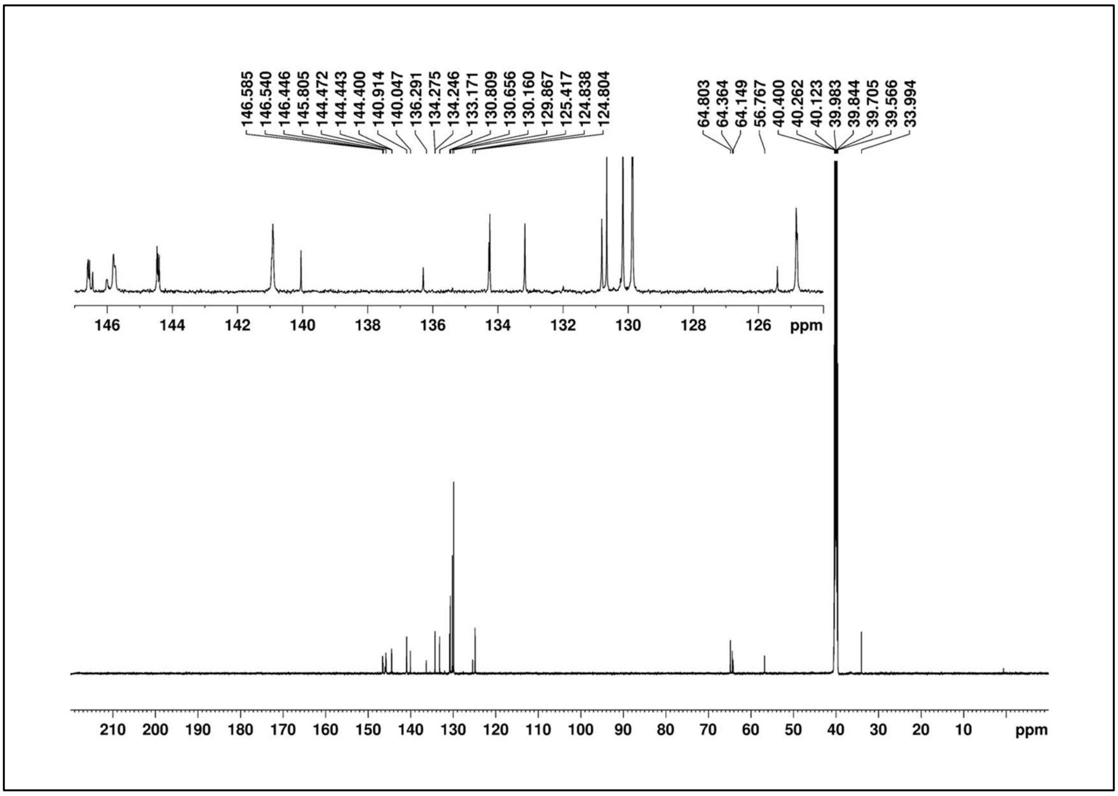

Figure S44: ^{13}C NMR (DMSO-d$_6$, 100 MHz) spectra of **2**

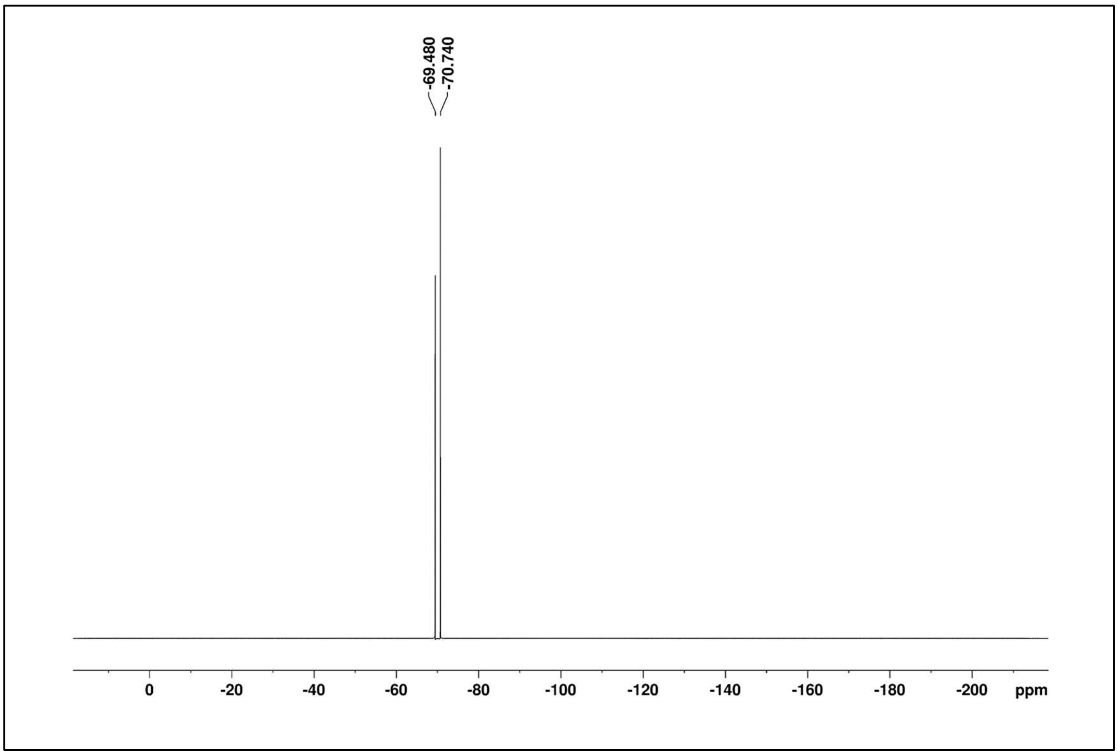

Figure S45: ^{19}F NMR (DMSO-d$_6$, 376 MHz) spectra of **2**

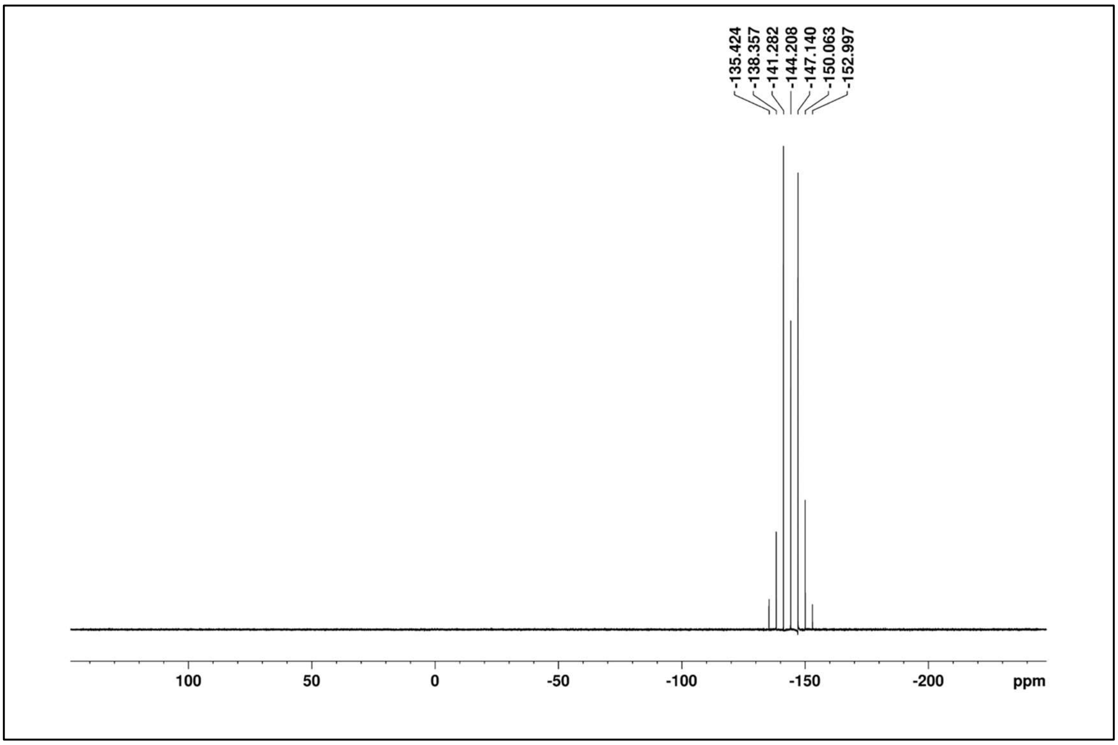

Figure S46: ^{31}P NMR (DMSO-d$_6$, 162 MHz) spectra of **2**

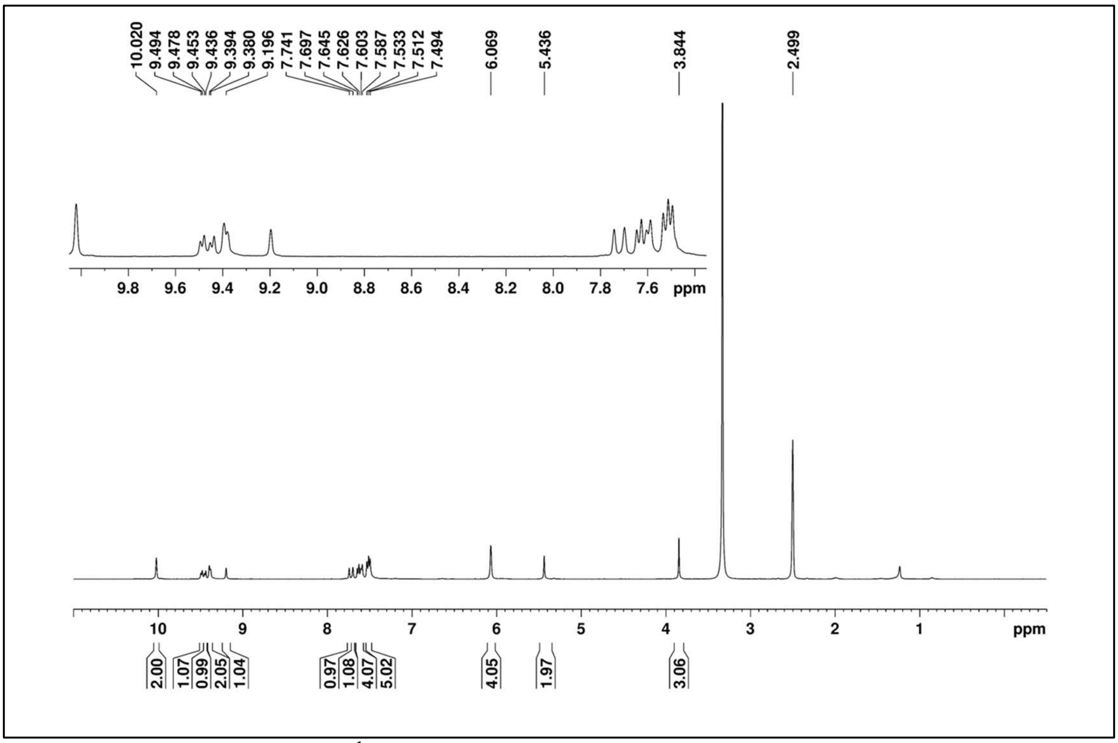

Figure S47: ^{1}H NMR (DMSO-d_6, 400 MHz) spectra of **3**

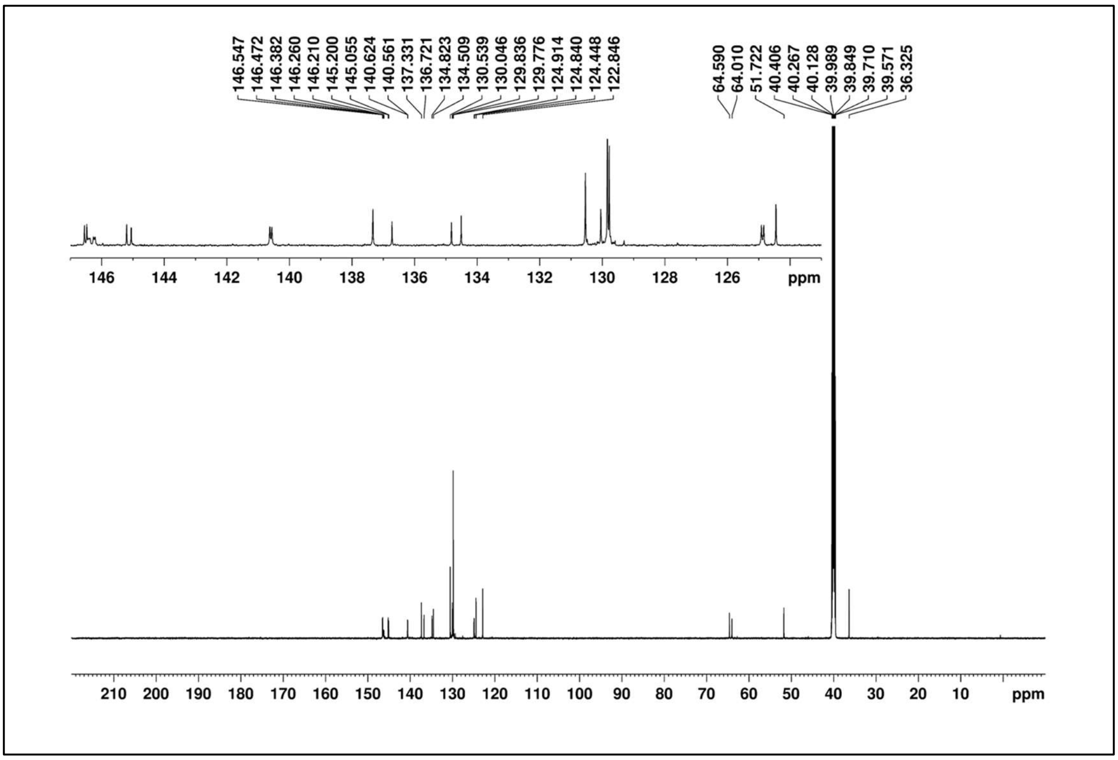

Figure S48: ^{13}C NMR (DMSO-d_6, 100 MHz) spectra of **3**

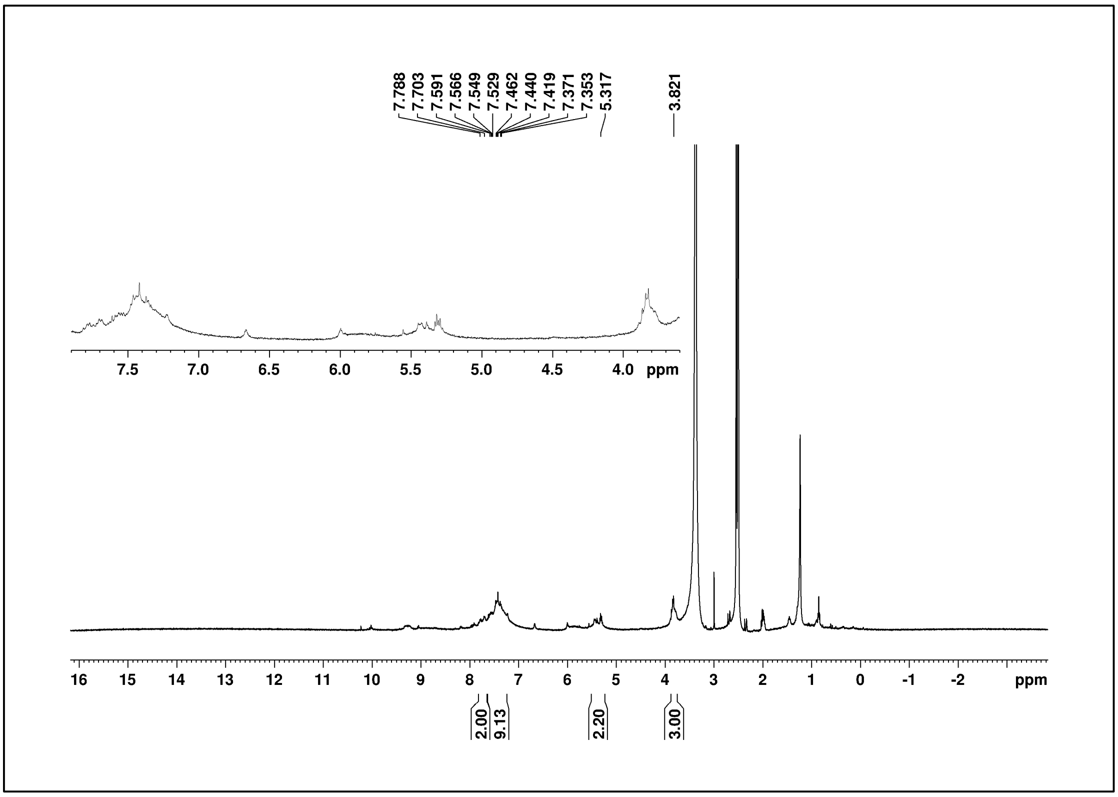

Figure S49: ^{1}H NMR (DMSO-d6, 400 MHz) spectra of **SeV^{2+}-NHC-PtNPs**

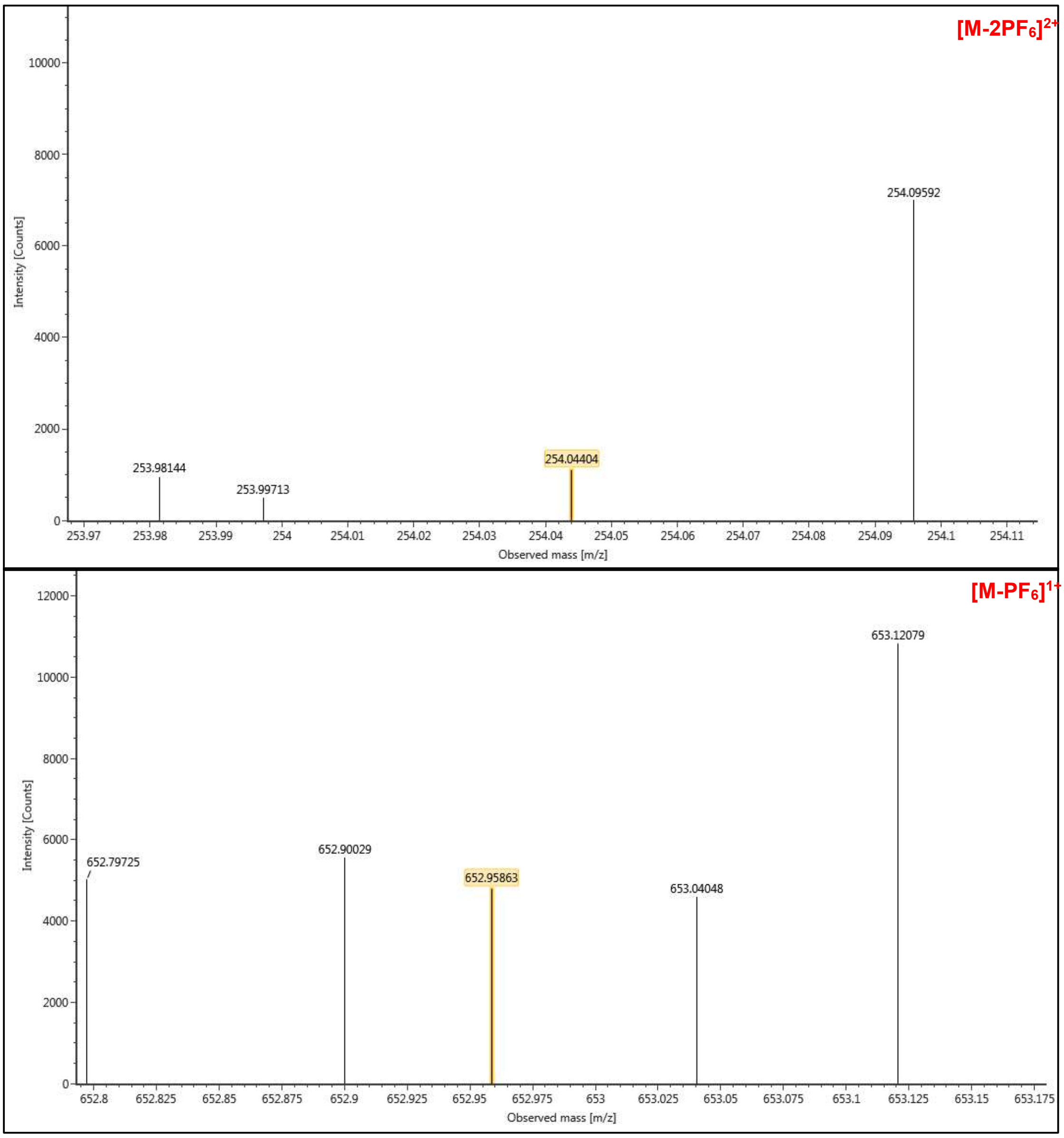

Figure S50: HRMS of **2**.

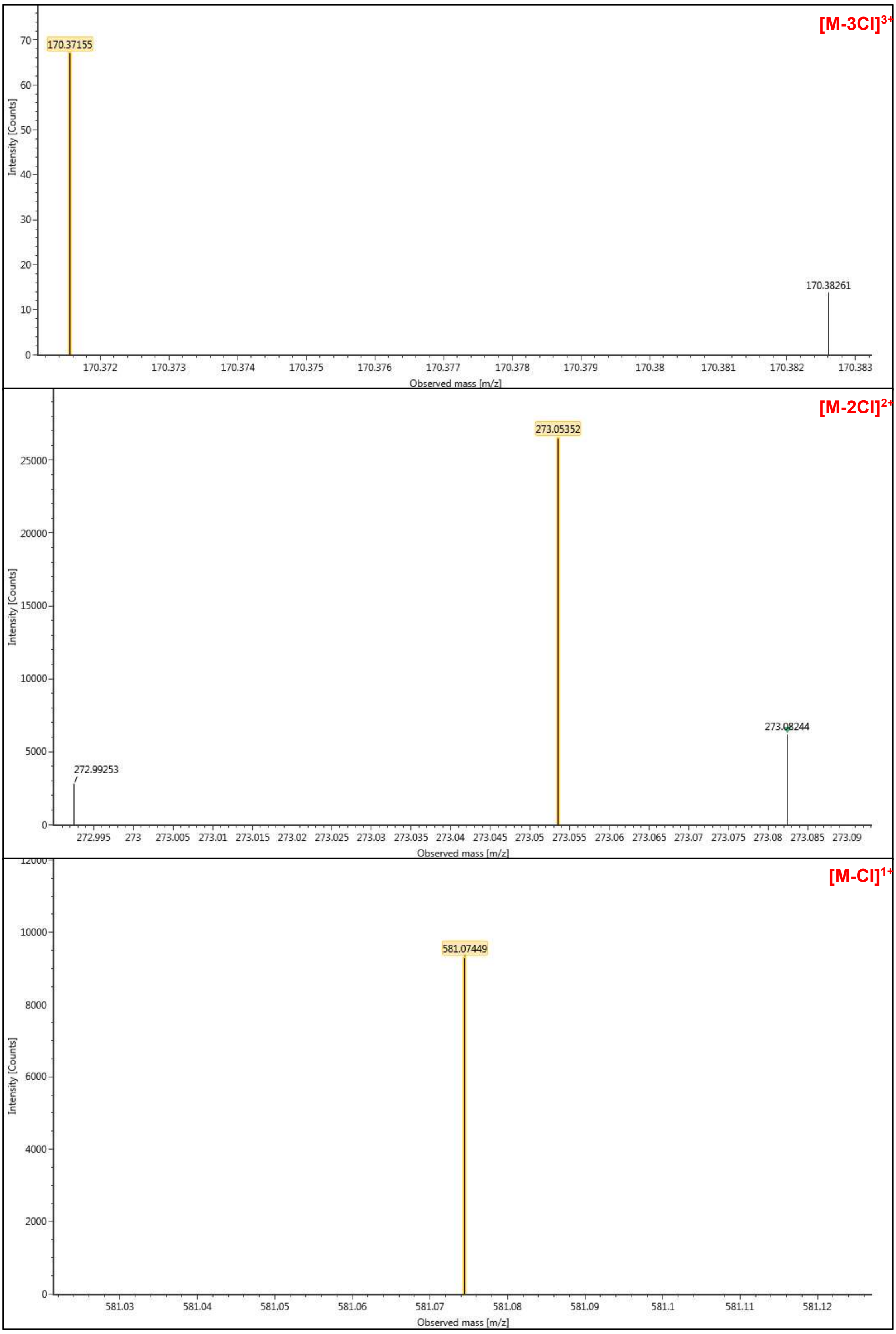

Figure S51: HRMS of **3**.

21. Reference

1. G. Li, K. Zhou, Q. Sun, et al., "Bacteria-Triggered Solar Hydrogen Production Via Platinum (II)-Tethered Chalcogenoviologens," *Angewandte Chemie International Edition* 61, no. 11 (2022): e202115298.
2. C. Eaborn, K. Kundu and A. Pidcock, "Synthesis of Platinum (II) Alkyl and Aryl Complexes from K_2 [$PtCl_4$] and Tetraorganotin Compounds in Dimethyl Sulphoxide," *Journal of the Chemical Society, Dalton Transactions*, no. 4 (1981): 933–938.
3. G. Li, L. Xu, W. Zhang, et al., "Narrow-Bandgap Chalcogenoviologens for Electrochromism and Visible-Light-Driven Hydrogen Evolution," *Angewandte Chemie International Edition* 57, no. 18 (2018): 4897–4901.
4. J. Tomasi, B. Mennucci and R. Cammi, "Quantum Mechanical Continuum Solvation Models," *Chemical Reviews* 105, no. 8 (2005): 2999–3094.
5. P. J. Stephens, F. J. Devlin, C. F. Chabalowski and M. J. Frisch, "Ab Initio Calculation of Vibrational Absorption and Circular Dichroism Spectra Using Density Functional Force Fields," *The Journal of physical chemistry* 98, no. 45 (1994): 11623–11627.
6. C. E. Check, T. O. Faust, J. M. Bailey, B. J. Wright, T. M. Gilbert and L. S. Sunderlin, "Addition of Polarization and Diffuse Functions to the LANL2DZ Basis Set for P-Block Elements," *The Journal of Physical Chemistry A* 105, no. 34 (2001): 8111–8116.
7. S. Zhang, L. Ma, W. Ma, et al., "Selenoviologen-Appendant Metallacycles with Highly Stable Radical Cations and Long-Lived Charge Separation States for Electrochromism and Photocatalysis," *Angewandte Chemie International Edition* 61, no. 42 (2022): e202209054.
8. N. Murakami, H. Miyake, T. Tajima, K. Nishikawa, R. Hirayama and Y. Takaguchi, "Enhanced Photosensitized Hydrogen Production by Encapsulation of Ferrocenyl Dyes into Single-Walled Carbon Nanotubes," *Journal of the American Chemical Society* 140, no. 11 (2018): 3821–3824.
9. X. Wang, L. Chen, S. Y. Chong, et al., "Sulfone-Containing Covalent Organic Frameworks for Photocatalytic Hydrogen Evolution from Water," *Nature chemistry* 10, no. 12 (2018): 1180–1189.
10. Z. Wang, D. Gao, H. Geng and C. Xing, "Enhancing Hydrogen Production by Photobiocatalysis through Rhodopseudomonas Palustris Coupled with Conjugated Polymers," *Journal of Materials Chemistry A* 9, no. 35 (2021): 19788–19795.

11. Z. Mi, T. Zhou, W. Weng, et al., "Covalent Organic Frameworks Enabling Site Isolation of Viologen-Derived Electron-Transfer Mediators for Stable Photocatalytic Hydrogen Evolution," *Angewandte Chemie International Edition* 60, no. 17 (2021): 9642–9649.
12. R. Chen, Y. Wang, Y. Ma, et al., "Rational Design of Isostructural 2D Porphyrin-Based Covalent Organic Frameworks for Tunable Photocatalytic Hydrogen Evolution," *Nature Communications* 12, no. 1 (2021): 1354.
13. X. Yang, B. Zhang, Y. Gao, et al., "Efficient Photoinduced Electron Transfer from Pyrene-O-Carborane Heterojunction to Selenoviologen for Enhanced Photocatalytic Hydrogen Evolution and Reduction of Alkynes," *Advanced Science* 9, no. 5 (2022): 2101652.
14. B. He, S. Zhang, Y. Zhang, et al., "Ortho-Terphenylene Viologens with through-Space Conjugation for Enhanced Photocatalytic Oxidative Coupling and Hydrogen Evolution," *Journal of the American Chemical Society* 144, no. 10 (2022): 4422–4430.
15. M. Martins, C. Toste and I. A. Pereira, "Enhanced Light-Driven Hydrogen Production by Self-Photosensitized Biohybrid Systems," *Angewandte Chemie International Edition* 60, no. 16 (2021): 9055–9062.
16. S. Altınışık, G. Yanalak, I. m. Hatay Patır and S. Koyuncu, "Viologen-Based Covalent Organic Frameworks toward Metal-Free Highly Efficient Photocatalytic Hydrogen Evolution," *ACS Applied Materials & Interfaces* 15, no. 15 (2023): 18836–18844.
17. Y. Qin, P. She, Y. Wang and W. Y. Wong, "An All-in-One Integrating Strategy for Designing Platinum (II)-Based Supramolecular Polymers for Photocatalytic Hydrogen Evolution," *Small* 20, no. 35 (2024): 2400259.
18. Y. Qin, C. Zhang, Y. Wang, P. She and W. Y. Wong, "Electrostatic Attraction-Driven Assembly of Non-Noble Metallo-Supramolecular Polymers with Single-Walled Carbon Nanotubes for Boosting Photocatalytic Hydrogen Evolution," *Carbon Energy*, no. (2025): e70003.
19. T. Whittemore, C. Xue, J. Huang, J. Gallucci and C. Turro, "Single-Chromophore Single-Molecule Photocatalyst for the Production of Dihydrogen Using Low-Energy Light," *Nature Chemistry* 12, no. 2 (2020): 180–185.
